# Three-Dimensionally Printed Ti2448 With Low Stiffness Enhanced Angiogenesis and Osteogenesis by Regulating Macrophage Polarization *via* Piezo1/YAP Signaling Axis

**DOI:** 10.3389/fcell.2021.750948

**Published:** 2021-11-15

**Authors:** Zhen Tang, Xinghui Wei, Tian Li, Hao Wu, Xin Xiao, Yulin Hao, Shujun Li, Wentao Hou, Lei Shi, Xiaokang Li, Zheng Guo

**Affiliations:** ^1^ Department of Orthopaedics, Xijing Hospital, Fourth Military Medical University, Xi’an, China; ^2^ School of Basic Medicine, Fourth Military Medical University, Xi’an, China; ^3^ Institute of Metal Research, Chinese Academy of Science, Shenyang, China; ^4^ Department of Orthopaedics, Tangdu Hospital, Fourth Military Medical University, Xi’an, China

**Keywords:** Ti2448, macrophage, polarization, angiogenesis, osteogenesis

## Abstract

Previous studies have found that the novel low-elastic-modulus Ti2448 alloy can significantly reduce stress shielding and contribute to better bone repair than the conventional Ti6Al4V alloy. In this study, the promotion of osteogenesis and angiogenesis by three-dimensionally printed Ti2448 were also observed *in vivo*. However, these were not significant in a series of *in vitro* tests. The stiffness of materials has been reported to greatly affect the response of macrophages, and the immunological regulation mediated by macrophages directly determines the fate of bone implants. Therefore, we designed more experiments to explore the role of three-dimensionally printed Ti2448 in macrophage activation and related osteogenesis and angiogenesis. As expected, we found a significant increase in the number of M2 macrophages around Ti2448 implants, as well as better osteogenesis and angiogenesis *in vivo*. *In vitro* studies also showed that macrophages pre-treated with Ti2448 alloy significantly promoted angiogenesis and osteogenic differentiation through increased PDGF-BB and BMP-2 secretion, and the polarization of M2 macrophages was enhanced. We deduced that Ti2448 promotes angiogenesis and osteogenesis through Piezo1/YAP signaling axis-mediated macrophage polarization and related cytokine secretion. This research might provide insight into the biological properties of Ti2448 and provide a powerful theoretical supplement for the future application of three-dimensionally printed Ti2448 implants in orthopaedic surgery.

## Introduction

Titanium (Ti) and its alloys have been widely applied in bone repair due to their excellent mechanical and chemical properties and biocompatibility (Sidhu et al., 2021). For example, the representative Ti6Al4V, an α + β-Ti alloy, has the advantages of excellent biocompatibility, corrosion resistance, non-magnetism and radiopacity (Sidhu et al., 2021). However, Ti6Al4V has poor osteogenic performance due to its high elastic modulus and potential biological toxicity (Kaur and Singh, 2019). To address these limitations, many methods have been used to optimize the performance of titanium alloys, including 3D printing porous structures, performing surface modifications, loading drugs, and developing new alloys (Liu et al., 2020).

**GRAPHICAL ABSTRACT:**
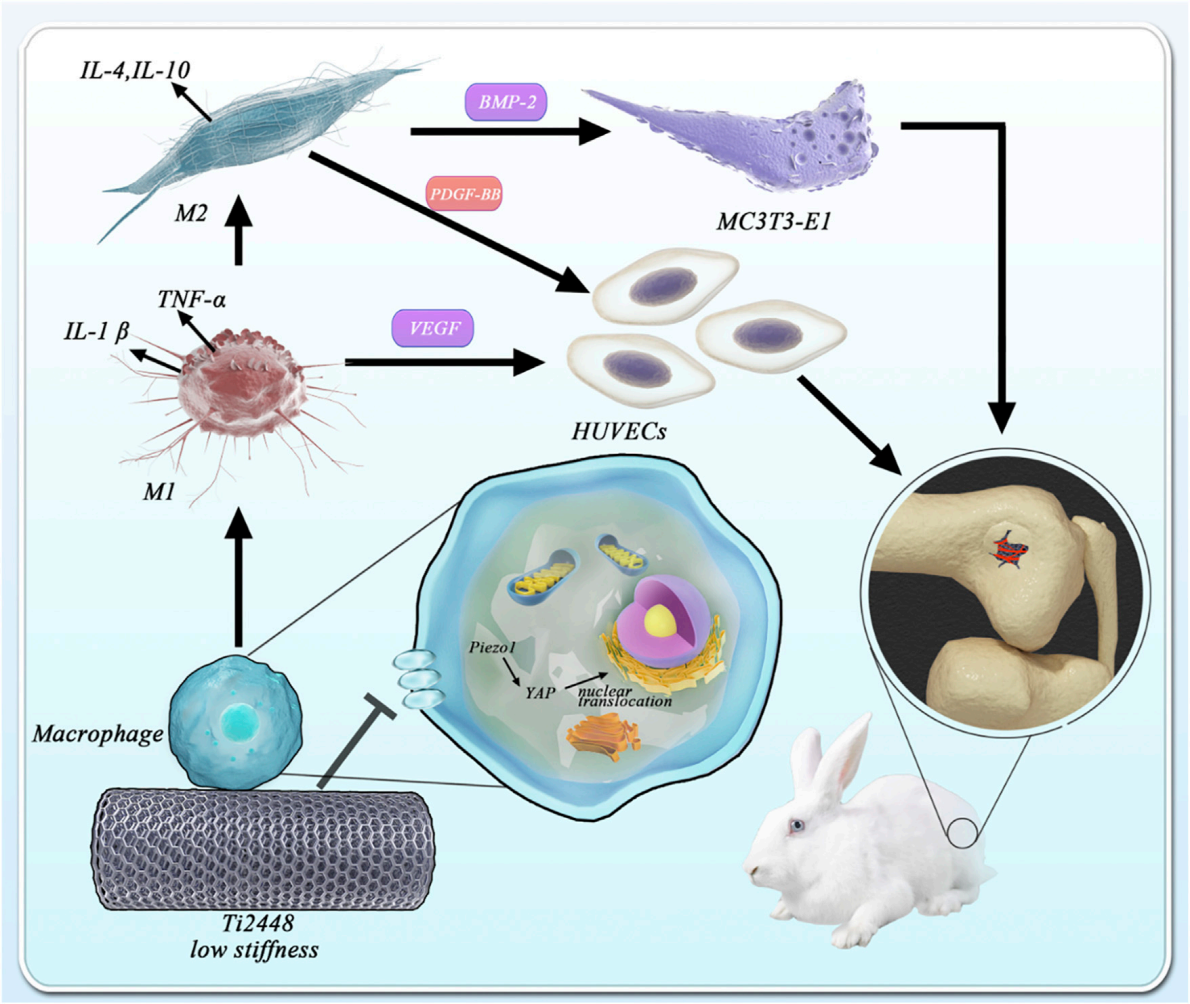


The novel Ti-24Nb-4Zr-7.9Sn (Ti2448) alloy is considered a better titanium-based biomaterial than Ti6Al4V. Y.L. Hao et al. showed that Ti2448 can achieve a better balance of high strength and low elastic modulus and is intended for biomedical applications (Hao et al., 2007). Yongquan Zhang et al. previously found that the bone histomorphometric parameters in the Ti2448 group were significantly better than those in the Ti6Al4V group, and the pullout force and new bone formation in the Ti2448 group were also significantly improved in full-thickness chondral defects in the bilateral knees of dogs (Zhang et al., 2013). Yang Qu et al. discovered that the stress-shielding effects of Ti2448 fixation were less than those of Ti6Al4V fixation (Qu et al., 2018). Previous studies have attributed the excellent osseointegration of Ti2448 to its biomechanical properties reducing the stress-shielding effect (Guo et al., 2020). Since then, due to its excellent biocompatibility, low elastic modulus and non-toxicity, Ti2448 has aroused great interest for application in bone repair (Zhan et al., 2020). However, the specific biological mechanism and involved effector cells are still unclear. Angiogenesis, the formation of new blood vessels from existing blood vessels, is a complex and highly regulated process that plays a role in a wide variety of physiological and pathological processes (Li T. et al., 2019; Gubbiotti et al., 2020; Wang L. et al., 2021). The osteogenic differentiation of osteoblasts and the angiogenesis of vascular endothelial cells are the two most important factors in the bone healing process. Some studies have shown that the behaviour of osteoblasts and vascular endothelial cells is modulated by Ti2448. The unique chemistry and 3D structure fabricated via EBM of the Ti2448 alloy was conducive to osteoblast adhesion, proliferation, differentiation, mineralization, and synthesis of proteins (Nune et al., 2017). In our studies, we seeded osteoblasts and vascular endothelial cells onto 3D-printed Ti2448 scaffolds, and the results revealed that osteogenesis-related gene expression, cell migration and tubule formation were not greatly changed in the Ti2448 group. These *in vitro* results might not reasonably account for satisfactory bone repair *in vivo*. Therefore, we speculate that there may be intermediate factors in the process of bone repair *in vivo*.

Macrophages play an important role in a wide variety of physiologic and pathologic processes (Cocks et al., 2021). Previous studies have found that osteogenic and angiogenic effects may be indirectly regulated through the immune response, in which macrophage polarization and cytokine secretion highly affect the fate of bone implants (Chen et al., 2020; Wang Y. et al., 2021; Li et al., 2021). Biomaterial implantation immediately stimulates the innate immune system and triggers a response that usually occurs in sequential stages, ranging from inflammation to tissue proliferation and maturation (Thorson et al., 2019; Zhao et al., 2020). The early and transient activation (1–5 days) of M1 macrophages is critical for cell infiltration and early vascularization, while the subsequent activation of M2 macrophages is beneficial for new bone formation and improving bone-implant integration in the late stage of bone repair (O’Brien et al., 2019; Zhu et al., 2020). Shuo Guo et al. demonstrated that the direction of macrophage polarization was regulated to promote angiogenesis *in vivo* and *in vitro* by a metal element-loaded nanorod array, thus promoting bone formation (Guo et al., 2020). Man Luo et al. successfully prepared IFN-γ/SrBG scaffolds that could promote mature bone formation in bone defects to a greater extent by polarizing macrophages towards the pro-inflammatory M1 type at the early stage of implantation by releasing IFN-γ and then polarizing macrophages towards the anti-inflammatory M2 type at a later stage by releasing Sr ions from SrBG (Luo et al., 2021). Stiffness of materials has been reported to greatly affect the response to immunological regulation. Hard matrix induced polarization of macrophages towards M1, while soft matrix induced formation of M2 macrophages (Jain et al., 2019). Anti-inflammatory phenotype (i.e., more M2 than M1) of macrophages can ultimately mitigate the stem cell survival conditions against inflammation (Kim et al., 2017). Based on the above studies, we studied the functional changes and polarization of macrophages *in vivo* and *in vitro* and the changes in the factor secretion profile *in vitro* to explore the potential biological mechanism of the osteogenic effect of Ti2448.

## Materials and Methods

### Scaffold Fabrication and Characterization

#### Scaffold Fabrication

As described above, porous and solid titanium alloy scaffolds were prepared by 3D printing. In brief, Ti6Al4V and Ti2448 scaffolds were designed using computer-aided design (CAD) software for *in vitro* and *in vivo* studies. The materials used for *in vitro* experiments were discs 14 mm in diameter (D) and 3 mm in height (H), and the scaffolds used for *in vivo* experiments were porous titanium alloy cylinders 6 mm in diameter (D) and 8 mm in height (H). The porous titanium alloy scaffolds had an aperture of 1,000 microns and porosity of 82.63%. In the process of preparing porous materials using an electron beam melting (EBM) system (Arcam A1, Arcam AB, Mölndal, Sweden). The particle size range of the raw material powder was consistent. Metal particles 45–106 µm in size were used for Ti6Al4V and Ti2448, and other particles of size were screened out. In the process of printing Ti6Al4V and Ti2448 by EBM, the parameters used for printing pores were consistent. When both powders were sintered at a suitable temperature (700°C for Ti6Al4V and 500°C for Ti2448), the porous materials were sintered at a uniform scanning current (3.4 mA) and speed (300 mm/s). Based on the above factors, porous materials with a uniform pore size and porosity could be obtained using the same granularity range of powder and the same sintering process. To better detect the expression level of related proteins by immunofluorescence, we used solid titanium alloy scaffolds instead of porous scaffolds.

#### Surface Characteristics

Energy-dispersive spectroscopy (EDS) and scanning electron microscopy (SEM, FE-SEM, S-4800, Hitachi, Japan) were used to detect the surface morphology and elemental composition of the scaffold, respectively. For both the Ti6Al4V and Ti2448 scaffolds, the scaffold was fixed on the tray with a conductive adhesive, and platinum was sprayed on the surface of the scaffolds by an E-1010 ion sputter-coating machine (SUPRO instrument) to improve the electrical conductivity of the material.

Surface roughness parameters of the Ti6Al4V and Ti2448 samples were analysed using an atomic force microscopy (AFM, SPM-9500J3, Japan). ACTA cantilever (Olympus) was used to scan the samples in non-contact mode. Size of images was recorded at 2 × 2 µm. Images were processed using SPIP software (Image Metrology A/S, Denmark) and roughness parameters were obtained from the scan size.

Water contact angle measurements were performed to analyse the hydrophilicity of the different surfaces. The surface water contact angle of Ti6Al4V and Ti2448 was detected with a contact angle goniometer (DataPhysics, Germany). Briefly, a distilled water dropt contacted the surface, and SCA20 software was used to record the shape of the droplet and calculate the water contact angle. To better detect the water contact angle and roughness on the surface of the material, we used solid scaffolds instead of porous scaffolds.

#### Structural Characterization

The scaffold structure was characterized by qualitative micro-computed tomography (micro-CT) (Y. Cheetah, YXLON, Germany). For both the Ti6Al4V and Ti2448 scaffolds, after the scaffold was placed in the micro-CT device and fixed with tape, the scanning parameters were set as follows: resolution, 13 μm; angle of rotation, 360°; voltage, 80 kV; and current, 500 μA. The obtained data in 2D and 3D were analysed using FGUI 3.0 software and VG Studio 2.1 software, and the macropore size and porosity of the scaffolds were analysed using VG Studio 2.1 software.

#### Mechanical Properties

A mechanical testing instrument (Instron 8872, Instron, United States) was applied to test the mechanical properties of the scaffolds. After each scaffold was fixed to the testing instrument with the longitudinal axis in the direction of the force, the probe of the hydraulic mechanical instrument gradually pressurized the scaffold at a rate of 1 mm/min until breakage. The elastic modulus and compressive strength of the scaffold were obtained during the test.

### Cell Culture

Human umbilical vein endothelial cells (HUVECs), murine-derived RAW 264.7 macrophages (RAW) and mouse osteogenic precursor MC3T3-E1 cells were purchased from the American Type Culture Collection (ATCC). HUVECs were cultured with a mixture of endothelial cell medium (ECM, ScienCell, United States), 5% foetal bovine serum (FBS), 1% endothelial cell growth supplement/heparin (ECGS/H, Promocell) and 1% penicillin/streptomycin. RAW cells and MC3T3-E1 cells were cultured with Dulbecco’s modified Eagle’s medium (DMEM, HyClone, United States) containing 5% FBS and 1% penicillin/streptomycin. The cells were cultured at 37°C in an atmosphere of 5% CO_2_.

### Assessment of Angiogenic Effect on HUVECs

Endothelial cell migration ability was estimated by Transwell invasion assays. HUVECs were seeded onto the surface of Ti6Al4V and Ti2448 samples and into 24-well plates at a density of 2 × 10^4^/ml with ECM complete medium and cultured for 2 days. Then, the cells from the surface of the Ti6Al4V and Ti2448 samples and 24-well plates were seeded into the top chamber, containing Matrigel (BD Biosciences, United States), in serum-free medium, while the bottom chamber contained medium with 10% FBS. After cultivation for 48 h, a cotton swab was used to scrub the remaining cells from the top side of the membrane. The membrane was fixed with 4% paraformaldehyde for 20 min. After staining with crystal violet solution (Beyotime), the number of cells on the bottom side of the membrane was observed and counted under an Olympus light microscope, and differences in the cell number among the groups were determined.

After thawing at 4°C overnight, 50 µL of Matrigel (BD Biosciences, United States), free of growth factors, was applied to cover the bottom of each well of 96-well plates; then, resuspended HUVECs from the surface of the Ti6Al4V and Ti2448 samples and 24-well plates were seeded into each well. The density and volume of each well was 2 × 10^4^/ml and 200 μL, respectively. The 96-well plates were incubated for 12 h, and then an inverted microscope (Leica) was used to observe and evaluate vascularization. The tube length and the number of branch points were measured using Image-Pro Plus 6 software.

Immunofluorescence staining was used to detect the angiogenic marker CD31 and the cytoskeleton of HUVECs. HUVECs were seeded onto the surface of Ti6Al4V and Ti2448 samples and into 24-well plates at a density of 2 × 10^4^/ml. Samples were fixed with 4% paraformaldehyde at room temperature for 30 min and then permeabilized with 0.5% Triton. The cells were incubated with mouse anti-human CD31 primary antibody (1:100 dilution; Abcam; ab24590) overnight at 4°C and then incubated with donkey anti-mouse IgG antibody-labelled FITC for 1 h. FITC-phalloidin treatments were applied to assess the cell morphology. DAPI was applied to stain the nuclei. Immunofluorescence microscopy was used to capture images.

### Evaluation of Osteogenic Effect *in vitro*

MC3T3-E1 cells were seeded onto the surface of Ti6Al4V and Ti2448 samples and into 24-well plates at a density of 2 × 10^4^/ml with DMEM. Transmission electron microscopy and immunofluorescence staining of the cytoskeleton were used to observe the morphology of MC3T3-E1 cells in different groups. The morphology of MC3T3-E1 cells on the surface of the titanium alloys was observed by field emission (FE)-SEM (JEOL JSM-6700F, Japan). After 2 days of culture, the samples were fixed with 4% glutaraldehyde. The cells were then dehydrated by a fractional ethanol series (10–100%) and sputter-coated with platinum. Immunofluorescence staining of the cytoskeleton was performed as described in section *Assessment of Angiogenic Effect on HUVECs*.

#### Alizarin Red Staining of MC3T3-E1 Cells

MC3T3-E1 cells were cultured in osteogenic medium containing dexamethasone (10 mg/ml), sodium β-glycerophosphate (10 μg/ml), and ascorbic acid (50 μg/ml). MC3T3-E1 cells were stained with 40 mM alizarin red solution and observed under a stereoscope after induction for 21 days. The mineralized nodules were dissolved, and the absorbance at 630 nm was measured for semiquantitative analysis.

The expression of osteopontin (OPN) and runt-related transcription factor (Runx2) in MC3T3-E1 cells in different groups was detected by western blot. The cells were lysed with RIPA buffer (Beyotime) at 4°C for 30 min. SDS-PAGE was used to separate proteins, which were then transferred onto PVDF membranes. After blocking with 5% non-fat dried milk, the membranes were incubated with primary antibodies against OPN (1:1000; Abcam; ab63856), Runx2 (1:1000; AntiProtech; PA0631) and β-actin (1:2000, Abcam, ab213262) overnight at 4°C. The membranes were washed with Tris-buffered saline supplemented with 0.05% Tween 20 (TBST). Then, the membranes were incubated with horseradish peroxidase (HRP)-labelled goat anti-rabbit secondary antibody at a 1:3000 dilution. An Amersham Imager 600 was used to image the protein bands.

### Detection of Polarization Regulation in Macrophages

RAW cells were seeded onto the surface of Ti6Al4V and Ti2448 samples and into 24-well plates at a density of 1 × 10^5^/ml with DMEM. Enzyme-linked immunosorbent assay (ELISA) was used to detect proteins secreted by macrophages, including TNF-α, IL-1β, IL-4, IL-10, VEGF, PDGF-BB and BMP-2. After 3 and 8 days, the medium was collected, centrifuged and filtered. ELISA kits (MultiSciences Biotech Co., Ltd., Hangzhou, Zhejiang, China) were used to detect the secretion of proteins associated with inflammation, angiogenesis, and osteogenesis according to the manufacturer’s instructions.

The morphology of macrophages on the surface of the titanium alloys was observed by FE-SEM (JEOL JSM-6700F, Japan). After 3 days of culture, the samples were fixed with 4% glutaraldehyde. The cells were then dehydrated by a fractional ethanol series (10–100%) and sputter-coated with platinum.

As described above, the expression of the inflammation-related proteins inducible nitric oxide synthase (iNOS, marker for M1 macrophages) and arginase-1 (Arg-1, marker for M2 macrophages) was detected by immunofluorescence with primary antibodies against iNOS (1:100; Abcam; ab178945) and Arg-1 (1:100; Abcam; ab239731). The expression of Piezo1, YAP, P-paxillin and vinculin in macrophages were detected by immunofluorescence with primary antibodies against Piezo1 (1:200; Invitrogen; MA5-32876), YAP (1:200; Affinity; DF3182), P-paxillin (1:200; Cell Signaling Technology; #69363) and vinculin (1:20; Proteintech; 2B5A7). Western blotting was used to analyse the expression of Piezo1 and YAP with primary antibodies against Piezo1 (1:1000; Invitrogen; MA5-32876) and YAP (1:1000; Affinity; DF3182).

To exclude the influence of the different compositions of the Ti6Al4V and Ti2448 scaffolds on the regulation of macrophage polarization, Ti6Al4V and Ti2448 scaffolds were immersed in DMEM for 3 days to prepare extracts, which were saved as conditioned medium D (CMD, denoted as CMD-Ti6Al4V and CMD-Ti2448). RAW cells were seeded onto the samples at a density of 1 × 10^5^/ml with CMD and cultured for 3 days. The expression of iNOS and Arg-1 was detected by immunofluorescence with primary antibodies against iNOS (1:100; Abcam; ab178945) and Arg-1 (1:100; Abcam; ab239731). Western blotting was used to analyse the expression of iNOS and Arg-1 with primary antibodies against iNOS (1:1000; Abcam; ab178945) and Arg-1 (1:1000; Abcam; ab239731). ELISA kits (MultiSciences Biotech Co., Ltd., Hangzhou, Zhejiang, China) were used to detect the secretion of the abovementioned proteins.

### Angiogenic and Osteogenic Effects Regulated by Macrophage Polarization

On the basis of previous research methods, we constructed an indirect coculture system consisting of RAW cells and HUVECs (Supplementary Figure S1). After 3 days of culture, extracts collected from macrophages were saved as conditioned medium A (CMA). CMA was mixed with ECM or DMEM at a ratio of 1:1, and the mixture was saved as conditioned medium B (CMB) or conditioned medium C (CMC), respectively. HUVECs were seeded into 24-well plates, and the medium was replaced with CMB. Then, we continued to prepare the sample as described above. Immunofluorescence detection of the angiogenic marker CD31 and cytoskeleton was performed as previously described. As described above, a cell invasion assay was performed to evaluate the invasion of HUVECs, and we detected angiogenesis by tubule formation assay.

To test the migration of HUVECs cultivated with all kinds of CMB, 5 × 10^5^ cells were seeded into each well of 6-well plates to perform the wound healing assay. When the cells reached 90–95% confluence, a straight line was made in the middle of each well by scraping. At 0, 6 and 12 h after scraping, all cells were observed under an inverted microscope. The distance between the two edges of the scratch (wound width) was calculated using Image-Pro Plus 6 software. The two edges of the scratch (wound width) were indicated by black lines.

To detect the mineralization of MC3T3-E1 cells, the medium was replaced with CMC, and the cells were induced for 21 days. Alizarin red staining was then performed. MC3T3-E1 cells were cultured in CMC for 2 days, staining of the cell cytoskeleton was observed by immunofluorescence, and the status of cells on the material surface was visualized by SEM. The expression of OPN and Runx2 was analysed by western blot.

### Animal Experiments *in vivo*

#### Establishment of Femoral Condylar Implant Model

Adult male New Zealand white rabbits were provided by the Animal Laboratory Centre of the Fourth Military Medical University. All procedures were approved by the Fourth Military Medical University Ethics Committee in accordance with guidelines and regulations. The femoral condylar implant model was applied in this study. The femoral epiphysis was exposed after an intramuscular injection of sodium pentobarbital (30 mg/kg body weight). A canal with a diameter of 6 mm and a depth of 8 mm was created with an electric drill in the direction of the metaphysis perpendicular to the long axis of the femur. The canal was then rinsed with normal saline to remove bone particles, and a Ti2448 or Ti6Al4V scaffold was gently implanted.

#### Establishment of Subcutaneous Implantation Model

Each scaffold was sterilized before implantation. The hair on the rabbit’s back was shaved, followed by disinfection of the skin by wiping with 70% ethanol. The scaffolds were inserted into a subcutaneous pouch made using curved artery forceps through the incision in the back, with each implantation site 4 cm apart. The animals were euthanized with an overdose of anaesthetic at 3 days, 1 and 2 weeks after implantation, and the implants were surgically removed for histological analysis.

#### Angiography and Visualization Analysis

After 2, 4 and 8 weeks, rabbits were anaesthetized for microangiography. The abdominal aorta and postcava were exposed through a median abdominal incision. The postcava and abdominal aorta were incised and ligated proximally. After a tube was inserted into the abdominal aorta and the lower limb vessels were flushed with approximately 1 L of normal saline containing heparin sodium (50 IU/ml), the lower limb vessels were fixed with 500 ml of 4% formaldehyde through the abdominal aorta. Subsequently, 50 ml of Microfil® silicone rubber injection compound (Flow Tech, Inc., Carver, MA) was injected through the abdominal aorta to infuse the lower limb vessels. All samples were harvested and fixed for 2 weeks with 4% formaldehyde. Then, all samples were treated with 10% EDTA as a decalcifying solution for 2 months. The blood vessels around the scaffolds were evaluated by micro-CT, and the blood vessels inside the scaffolds were observed by serial transverse sectioning.

#### Micro-CT Assessment

New bone tissue and blood vessels around and inside the scaffolds were analysed by micro-CT (Y.Cheetah, YXLON, Germany). The scanning parameters were as follows: resolution, 13 μm; angle of rotation, 360°; voltage, 80 kV; and current, 500 μA. To detect bone ingrowth, we chose the total scaffold within a cylinder with a diameter of 6 mm and a height of 8 mm as the region of interest (ROI). To detect angiogenesis, a 2-mm range around the scaffolds was chosen as the ROI. VG Studio 2.1 software was used to reconstruct 3D images. The percentage of bone volume/total volume (BV/TV) and the volume of blood vessels in the ROI were used to evaluate bone ingrowth and angiogenesis, respectively.

#### Histological and Histomorphometric Analyses

After micro-CT analysis, the samples were prepared for serial transverse sectioning at 70 μm, and the sections were then subjected to van Gieson (VG) staining according to the protocol. Acquired images were analysed using Image-Pro Plus 6.0 software. The new bone tissue content (BV/TV) and degree of bone-implant contact were calculated using the following formulas:

$$\text{BV}/\text{TV}=\frac{\text{volume of new bone tissue}}{\text{volume of interest}}\times \;100\text{\%}$$

$$\text{Bone}–\text{implant contact}=\frac{\text{perimeter of the material}-\text{bone tissue contact surface}}{\text{inner and outer perimeter of the material}}\times \;100\text{\%}$$

The samples were prepared into 300-μm-thick sections to observe blood vessels inside the scaffolds with an Olympus microscope under a fluorescent background (wavelength, 430–460 nm). Image-Pro Plus 6.0 software was used to quantitatively analyse the number of blood vessels, the total length of the blood vessels, the longest blood vessel length, and the maximum blood vessel diameter in the different groups.

After 2 months of decalcification, samples of the femoral condyle and subcutaneous tissues were processed for haematoxylin and eosin (HE) staining and histological immunofluorescence analysis using rabbit anti-rabbit CD31 (1:100, Affinity, AF6191), rabbit anti-rabbit CD68 (1:100, Affinity, AF7518), rabbit anti-rabbit CCR7 (1:100, Affinity, AF5293) and guinea pig anti-rabbit Arg-1 (1:100, Cloud-Clone, PAB120Rb51) antibodies. Stained sections were observed by laser scanning confocal microscopy (LSCM, FV1000, Olympus, Japan). The white areas in the HE images and the black areas in the immunofluorescence images represent the titanium alloy scaffolds.

#### EDS Analysis

The samples were prepared into 1000-μm-thick slices that were sputter-coated with a thin layer of platinum to enhance their conductivity. The chemical composition and content of the samples were detected, and elemental EDS analysis was performed using an EMAX ENERGY system (S-4800, HITACHI, Japan). We considered the overlapping area of elemental Ti, Al and V to be the Ti6Al4V scaffold, the overlapping area of elemental Ti, Nb and Sn to be the Ti2448 scaffold, and the overlapping area of elemental Ca and P to be new bone.

### Statistical Analysis

All statistical tests were performed using GraphPad Software, and the data are presented as the mean ± standard deviation. Significant differences between groups were determined by one-way analysis of variance (ANOVA). These differences were considered statistically significant when the *p* < 0.05.

## Results

### Scaffold Characterization

**FIGURE 1:**
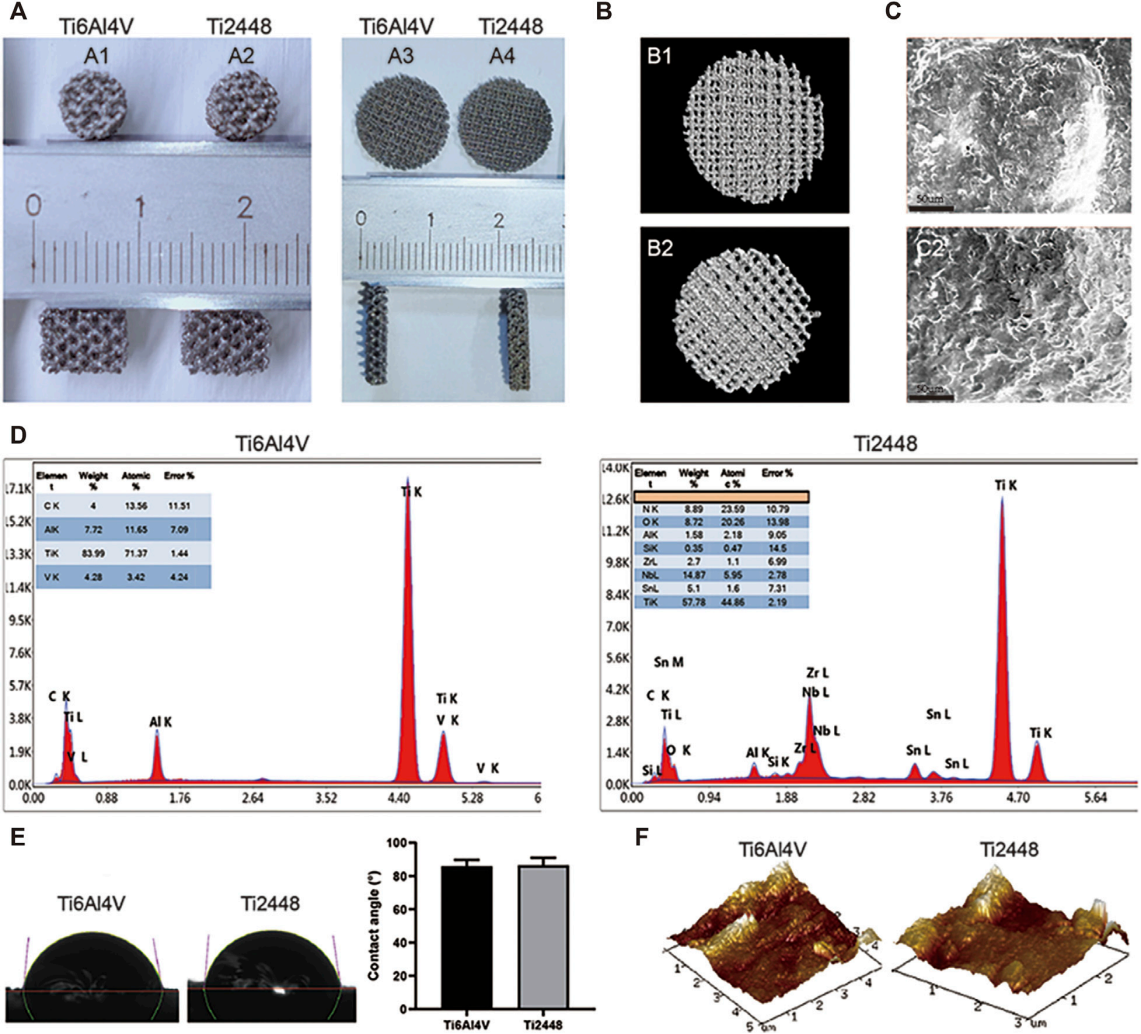
Characterization of Ti6Al4V and Ti2448 scaffolds. Ti6Al4V scaffold in animal experiments **(A1)** and cellular experiments **(A3)**; Ti2448 scaffold in animal experiments **(A2)** and cellular experiments **(A4)**. 3D reconstruction results of Ti6Al4V (B1) and Ti2448 (B2) scaffolds by micro-CT **(B)**. Surface topography of the Ti6Al4V (C1) and Ti2448 (C2) scaffolds, Scale bar = 50 µm **(C)**. EDS results of Ti6Al4V and Ti2448 showing their respective elemental compositions and surface morphology **(D)**. Water contact angle of the Ti6Al4V and Ti2448 scaffolds **(E)**. AFM images of Ti6Al4V and Ti2448 surfaces.

Both porous and solid Ti6Al4V and Ti2448 scaffolds were manufactured by EBM (Arcam A1, Arcam AB, Mölndal, Sweden), resulting in a uniform structure and no difference in overall structure (Figures 1A,B). There was no significant difference in porosity or pore size between the Ti6Al4V and Ti2448 scaffolds (Table 1). The pore diameter of the Ti6Al4V and Ti2448 scaffolds was 1,000 µm, and the porosity was 82.63%. SEM showed that the surface morphology of the two scaffolds was similar (Figure 1C). Ti6Al4V was composed of Ti, Al and V, and Ti2448 was composed of Ti, Nb, Zr and Sn (Figure 1D). The mechanical and structural parameters of the two scaffolds are shown in Table 1. The elastic modulus of the Ti6Al4V and Ti2448 scaffolds was 16.1 and 1.92 GPa, and the compressive strength was 11.64 ± 0.26 and 20.21 ± 0.11 MPa, respectively. The average water contact angle of the Ti6Al4V scaffolds was 85.84 ± 2.01°, while that of the Ti2448 scaffolds was 86.56 ± 2.26°. There is no significant difference in these values (Figure 1E, P > 0.05), indicating that the hydrophilicity of Ti2448 is similar to that of Ti6Al4V. The AFM images of the Ti6Al4V and Ti2448 sample surfaces are shown in Figure 1F. The Ti6Al4V and Ti2448 surface exhibited similar morphology.

**TABLE 1:** The mechanical and structural parameters of the scaffolds.

Group	Pore diameter (um)	Porosity (%)	Elastic modulus (Gpa)	Compressive strength (MPa)
Ti6Al4V	1,000	82.63%	16.1	11.64 ± 0.26
Ti2448	1,000	82.63%	1.92	20.21 ± 0.11

### Evaluation of Osteogenic Effect *in vivo*

**FIGURE 2:**
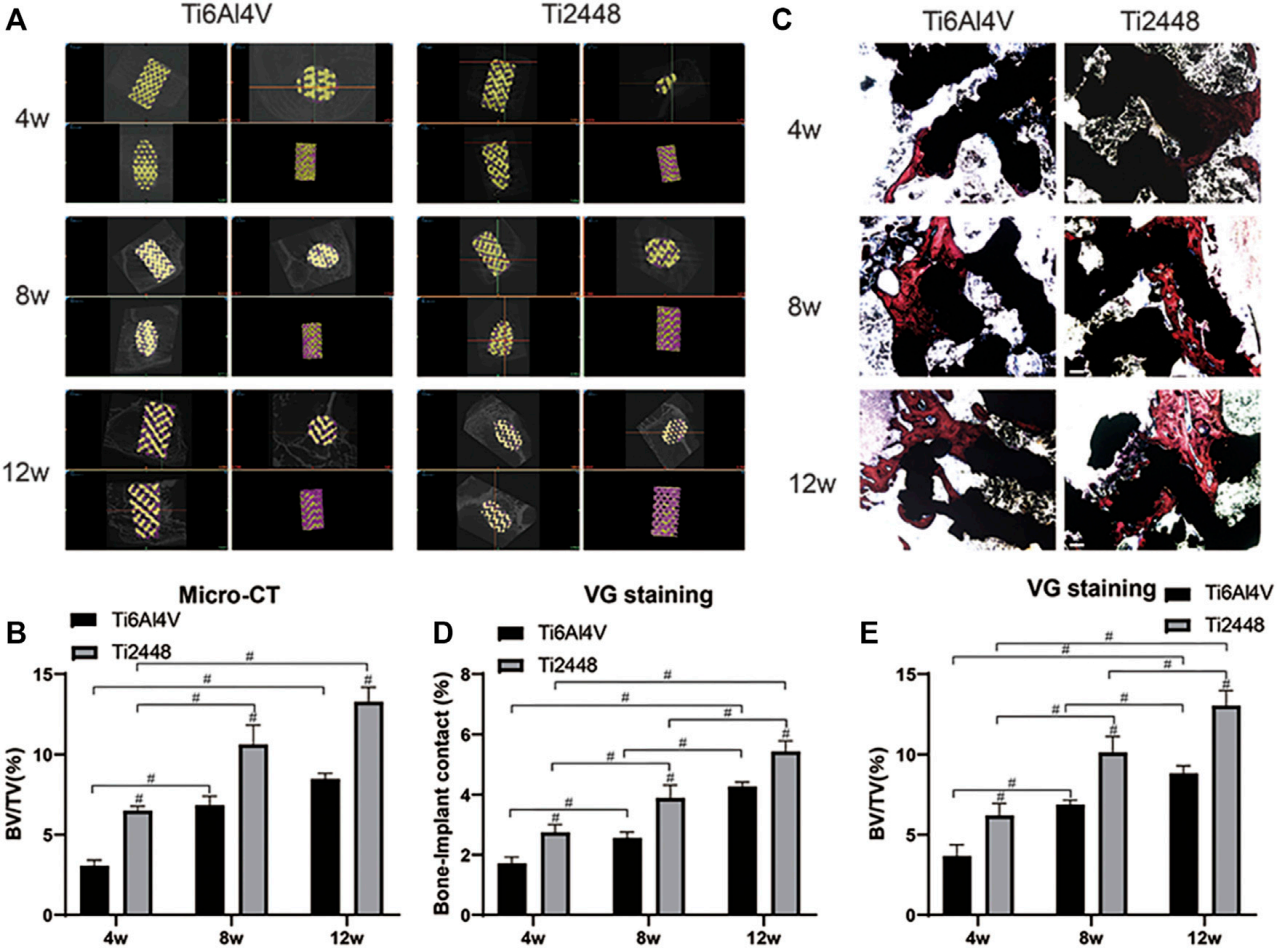
Micro-CT and histomorphometric analysis *in vivo*. New bone tissue around each scaffold reconstructed by micro-CT **(A)**. Quantitative analysis of the BV/TV for the different scaffolds at 4, 8 and 12 weeks **(B)**. Histomorphological analysis of inward growth and integration of bone tissue at corresponding time points. Scale bar = 200 µm **(C)**. Quantitative analysis of the bone−implant contact and BV/TV for different scaffolds at 4, 8 and 12 weeks **(D,E)**. Note: #*p* < 0.05 compared to Ti2448.

**FIGURE 3:**
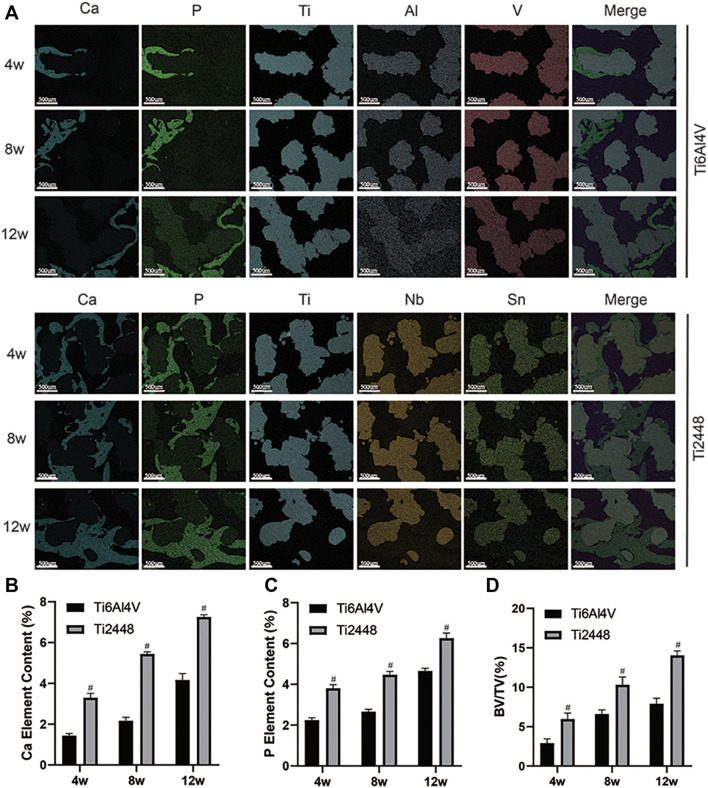
Assessment of osteogenesis by EDS. Elemental composition of different groups of scaffolds at 4, 8 and 12 weeks detected by SEM-EDS. Scale bar = 500 µm **(A)**. Quantitative analysis of the elemental Ca content of different scaffolds at 4, 8 and 12 weeks **(B)**. Quantitative analysis of the elemental P content of different scaffolds at 4, 8 and 12 weeks **(C)**. Quantitative analysis of the BV/TV of different scaffolds at 4, 8 and 12 weeks **(D)**. Note: #*p* < 0.05 compared to Ti2448.

Figure 2A shows representative 2D and 3D micro-CT images of the scaffolds in different groups at 4, 8, and 12 weeks. We observed the growth of new bone (purple) into the scaffolds (yellow) at the corresponding time points and the amount of bone tissue in each scaffold. The Ti2448 group showed better bone formation, while there was less bone formation in the Ti6Al4V group (Figure 2A). The Ti6Al4V group showed little new bone formation at 4 weeks (2.90% ± 0.65), which was much less than that in the Ti2448 group (6.35% ± 1.31) (*p* < 0.05). At 8 weeks, the BV/TV was significantly higher in the Ti2448 group (11.2% ± 3.04) than in the Ti6Al4V group (6.57% ± 2.51) (*p* < 0.05). At 8 weeks, much new bone tissue was observed in the centre of the Ti2448 scaffolds, while large areas of newly formed bone were rarely observed in the Ti6Al4V group, and new bone tissue failed to traverse the entire longitudinal axis of the implants. The amount of new bone tissue in the Ti2448 group (13.75% ± 3.62) was significantly higher than that in the Ti6Al4V group (8.32% ± 2.35) at 12 weeks (*p* < 0.05) (Figure 2B). In addition, histological evaluation of the bone formation in the two groups of scaffolds also showed that the bone in the Ti6Al4V group was incomplete or discontinuous. Endogenous bone was usually adjacent to the implants but not in close contact with the implants. Most of the bone tissue was concentrated in the peripheral area of the Ti6Al4V implants. However, in the Ti2448 group, bone tissue was deposited in the central area of the implants at 4 weeks. At 8 and 12 weeks after implantation, previously unconnected bone tissue in the Ti2448 scaffolds showed continuous trabeculae and intact and connected bone tissue, and the endogenously grown bone was in close contact with the implant (Figures 2C–E) (*p* < 0.05). This trend is similar to that shown by the micro-CT results. SEM-EDS was used to observe the surface elements of tissue sections. The overlapping area of elemental Ti, Al and V was considered to indicate the Ti6Al4V scaffold, the overlapping area of elemental Ti, Nb and Sn was considered to indicate the Ti2448 scaffold, and the overlapping area of elemental Ca and P was considered to indicate the new internal bone tissue (Figure 3A). At 4 weeks, the Ca and P levels in the Ti6Al4V group (1.44 ± 0.23%, 2.25 ± 0.32%) were lower than those in the Ti2448 group (3.30 ± 0.52%, 3.81 ± 0.38%), respectively (*p* < 0.05). At 8 weeks, the ratios of Ca and P were increased in both groups, and the proportions in the Ti2448 group (5.44 ± 0.26%, 4.47 ± 0.44%) were higher than those in the Ti6Al4V group (2.17 ± 0.23%, 2.67 ± 0.28%) (*p* < 0.05). The Ca and P levels in the Ti2448 group (7.26 ± 0.43%, 6.26 ± 0.35%) were significantly higher than those in the Ti6Al4V group (4.17 ± 0.63%, 4.67 ± 0.36%) at 12 weeks (*p* < 0.05). The amount of new bone tissue inside the Ti2448 scaffolds (5.97 ± 0.53%, 10.30 ± 1.82%, 14.03 ± 2.01%) was significantly higher than that inside the Ti6Al4V scaffolds (2.92 ± 0.33%, 6.60 ± 1.53%, 7.90 ± 2.15%) at 4, 8, and 12 weeks, respectively (*p* < 0.05) (Figures 3B–D). The trend shown by the SEM-EDS results is similar to that shown by the micro-CT and VG staining results.

### Evaluation of Angiogenic Effect *in vivo*

**FIGURE 4:**
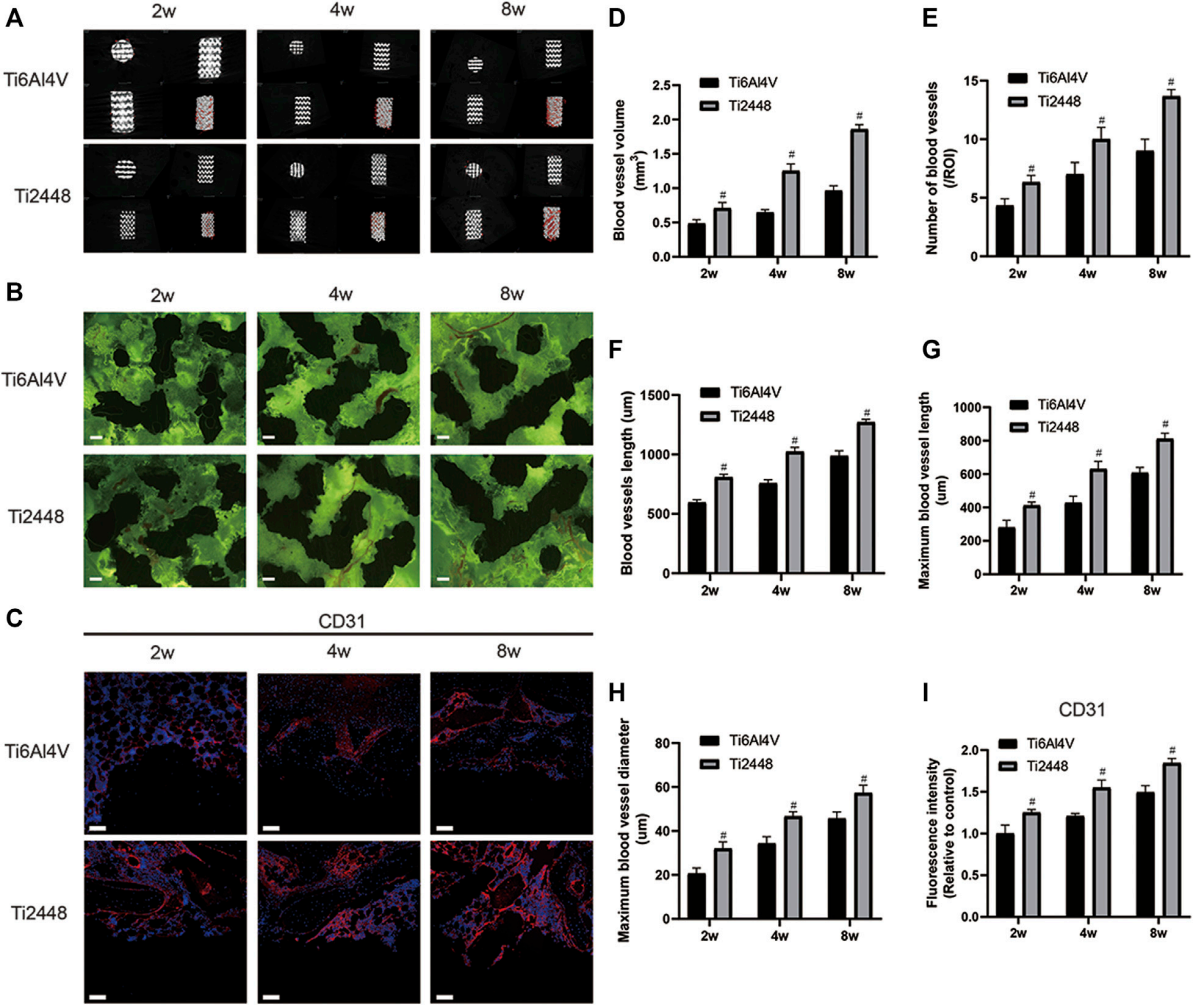
Analysis of vascularization around and inside the scaffolds. 3D reconstruction of blood vessels around different scaffolds at 2, 4, and 8 weeks **(A)**. Vascularization inside the scaffolds. Scale bar = 200 µm **(B)**. Immunofluorescence staining of CD31 in bone tissue. Scale bar = 100 µm **(C)**. Quantitative analysis of vascular volume around the scaffold **(D)**. Quantitative analysis of the number of blood vessels inside the scaffold in a single field of view **(E)**. Quantitative analysis of the total length of blood vessels inside the scaffold **(F)**. Quantitative analysis of the maximum single vessel length inside the scaffold **(G)**. Quantitative analysis of the maximum blood vessel diameter inside the scaffold **(H)**. Quantitative analysis of the fluorescence intensity of CD31 around the scaffold **(I)**. Note: #*p* < 0.05 compared to Ti2448.

Vessels around the scaffolds in both groups were examined by micro-CT (Figure 4A). At 2 weeks, the blood vessels around the materials were rare and thin, and the blood vessels in the Ti2448 group were denser and thicker than those in the Ti6Al4V group. At 4 weeks, mature and thick blood vessels formed around the scaffolds in both groups, and the interface between the scaffold material and bone showed better blood vessel formation in the Ti2448 group than in the Ti6Al4V group. At 8 weeks, the vessels around the scaffolds were more mature, and the vessels in the Ti2448 group were thicker than those in the Ti6Al4V group (Figure 4D). Fluorescence imaging was used to clearly observe the distribution and morphology of microvessels within the implants in hard tissue sections (Figure 4B). At 2 weeks, the vessels in both groups were immature, and only short and thin vessels could be seen inside the Ti6Al4V scaffold, while the vessels in the Ti2448 group were thicker and longer than those in the Ti6Al4V group. At 4 weeks, the vessels inside the scaffolds in the two groups tended to be more mature, as indicated by a significant increase in vessel diameter and length, and the vessels in the Ti2448 group were more mature than those in the Ti6Al4V group. At 8 weeks, the vessels inside the scaffolds in the two groups were more mature, and the diameter and length of the vessels in the Ti2448 group were greater than those in the Ti6Al4V group. We further quantitatively analysed the number of vessels, the total vessel length, the longest vessel length and the maximum vessel diameter inside the scaffolds. At weeks 2, 4, and 8, the Ti2448 group had the highest number of vessels, the longest total length of vessels, the longest vessel length, and the maximum vessel diameter inside the scaffolds (*p* < 0.05). At weeks 2, 4 and 8, the number of blood vessels in the Ti2448 group was higher than that in the Ti6Al4V group (*p* < 0.05), and the total vessel length in the Ti2448 group was 775.43 ± 47.73 µm, 1,094 ± 83.47 µm and 1,328 ± 63.73 µm, respectively (*p* < 0.05). At weeks 2, 4 and 8, the maximum vessel length in the Ti2448 group (406 ± 26.64 µm, 627 ± 27.93 µm, 826 ± 36.65 µm) was significantly longer than that in the Ti6Al4V group (*p* < 0.05). At weeks 2, 4 and 8, the maximum vessel diameter in the Ti2448 group was higher than that in the Ti6Al4V group (*p* < 0.05) (Figures 4E–H). The length and maximum diameter of a single vessel can also reflect the maturity of the vessel, indicating that the Ti2448 scaffold is more conducive to the maturation of vessels. Immunofluorescence staining for CD31, an angiogenic marker, showed stronger expression of CD31 around the Ti2448 scaffold than around the Ti6Al4V scaffold at 2, 4, and 8 weeks, and the fluorescence intensity gradually increased over time (Figures 4C,I).

### Evaluation of Inflammatory Response *in vivo*

**FIGURE 5:**
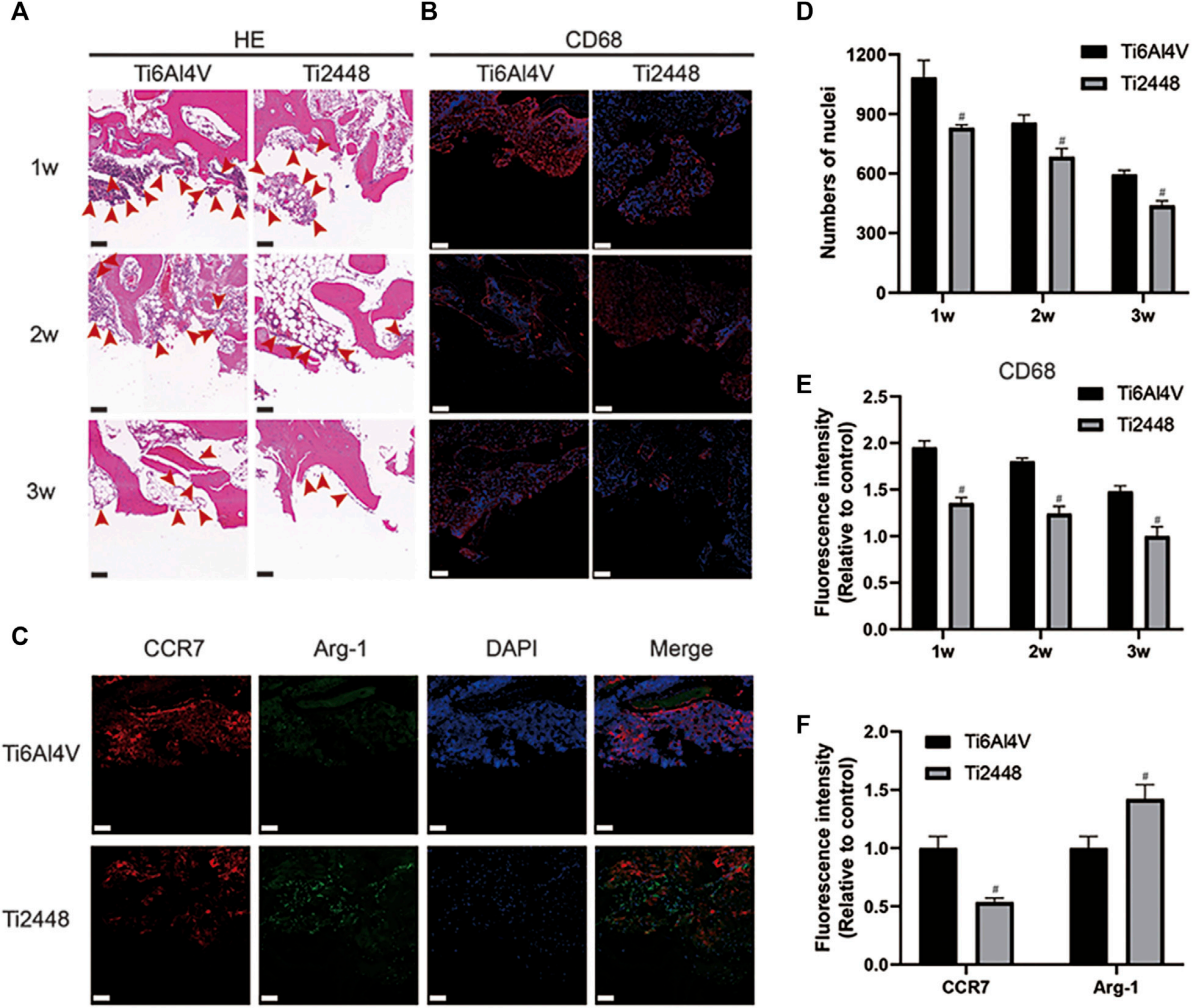
Inflammatory response to scaffolds evaluated and analysed in bone tissue. HE staining of bone tissue at 1, 2, and 3 weeks after implantation. Scale bar = 100 µm **(A)**. Evaluation of inflammation by immunofluorescence staining for the macrophage marker CD68 in bone. Scale bar = 200 µm **(B)**. Immunofluorescence staining for CCR7 (marker for M1 macrophages) and Arg-1 (marker for M2 macrophages) in the bone at 2 weeks. Scale bar = 100 µm **(C)**. Quantitative analysis of the number of nuclei around the scaffolds **(D)**. Quantitative analysis of the fluorescence intensity of CD68 around the scaffolds **(E)**. Quantitative analysis of the fluorescence intensity of CCR7 and Arg-1 around the scaffolds **(F)**. Note: #*p* < 0.05 compared to Ti2448.

The infiltration of inflammatory cells around the implant was observed by HE staining and immunofluorescence staining. The results showed that compared with the Ti6Al4V implants, there were fewer inflammatory cells around the Ti2448 implants at 1, 2 and 3 weeks (Figure 5A). Analysis of the number of nuclei showed fewer nuclei around the Ti2448 implants (*p* < 0.05) (Figure 5D). Staining for the macrophage marker CD68 was performed to demonstrate macrophage infiltration (Figure 5B). These results also showed that the number of inflammation-related macrophages around the Ti2448 implants was lower than that around the Ti6Al4V implants (Figure 5E). At 2 weeks, the immunofluorescence results for the M1 macrophage marker CCR7 (red) and M2 macrophage marker Arg-1 (green) indicated that the Ti2448 implants could effectively reduce the polarization of M1 macrophages and promote the polarization of macrophages towards the M2 phenotype (Figures 5C,F).

**FIGURE 6:**
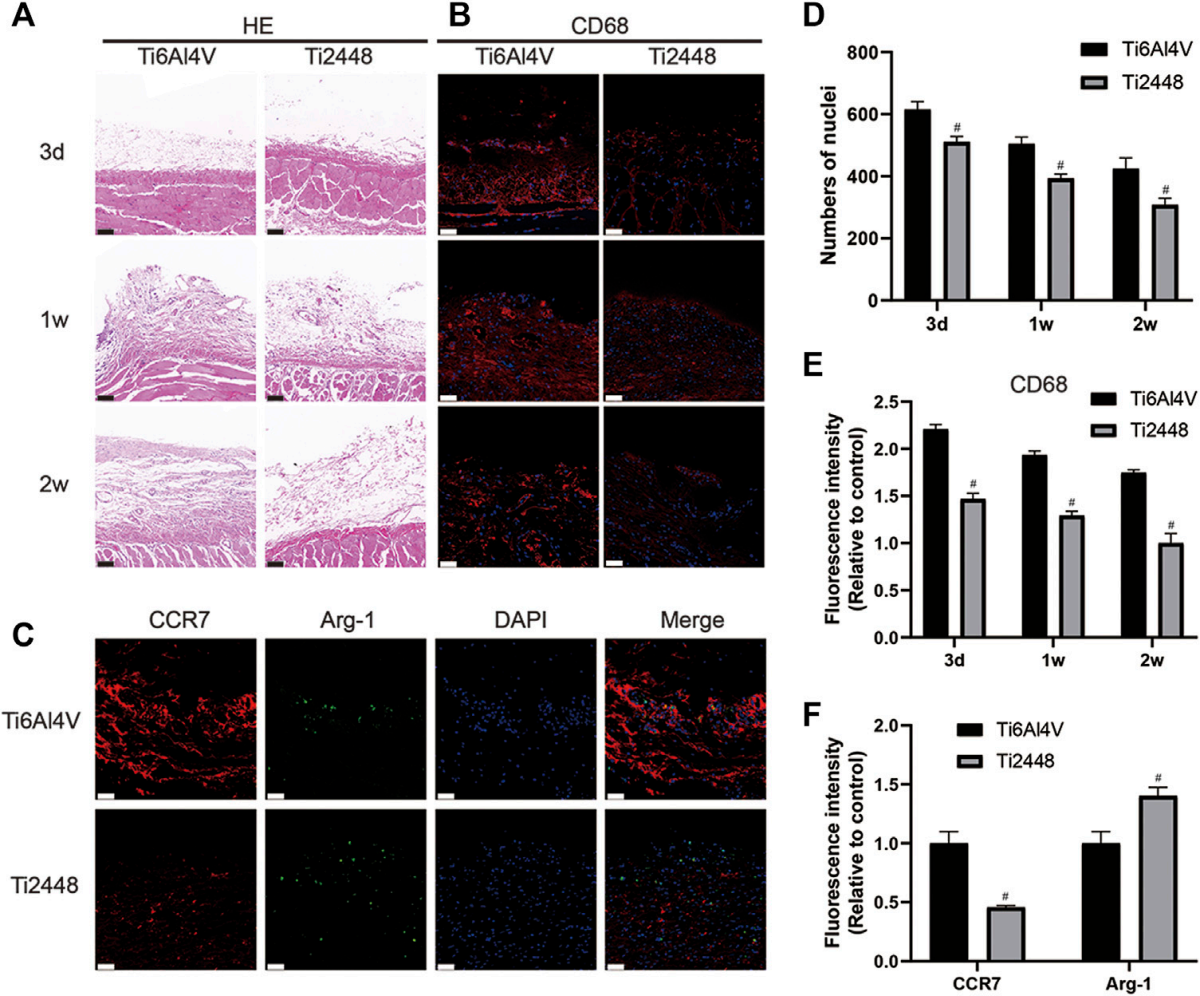
Inflammatory response to scaffolds evaluated and analysed in subcutaneous tissue. HE staining of skin specimens at 3 days, 1 and 2 weeks after implantation. Scale bar = 100 µm **(A)**. Evaluation of inflammation by immunofluorescence staining for CD68 in the skin. Scale bar = 50 µm **(B)**. Immunofluorescence staining for CCR7 (marker for M1 macrophages) and Arg-1 (marker for M2 macrophages) in the skin at 1 week. Scale bar = 50 µm **(C)**. Quantitative analysis of the number of nuclei around the scaffolds **(D)**. Quantitative analysis of the fluorescence intensity of CD68 around the scaffolds **(E)**. Quantitative analysis of the fluorescence intensity of CCR7 and Arg-1 around the scaffolds **(F)**. Note: #*p* < 0.05 compared to Ti2448.

The subcutaneous implant model also showed reduced inflammation around the Ti2448 implants. As shown in Figures 6A,D, the surface of the Ti2448 implants had less inflammatory cell infiltration and more soft tissue growth. Additionally, there were fewer CD68^+^ macrophages around the Ti2448 implants than around the Ti6Al4V implants (Figures 6B,E). The high expression of Arg-1 in the subcutaneous tissue around the Ti2448 implants indicates that this material has the potential to inhibit inflammation at 1 week (Figures 6C,F).

### Angiogenic Effect on HUVECs and Osteogenic Effect on MC3T3-E1 Osteoblasts

**FIGURE 7:**
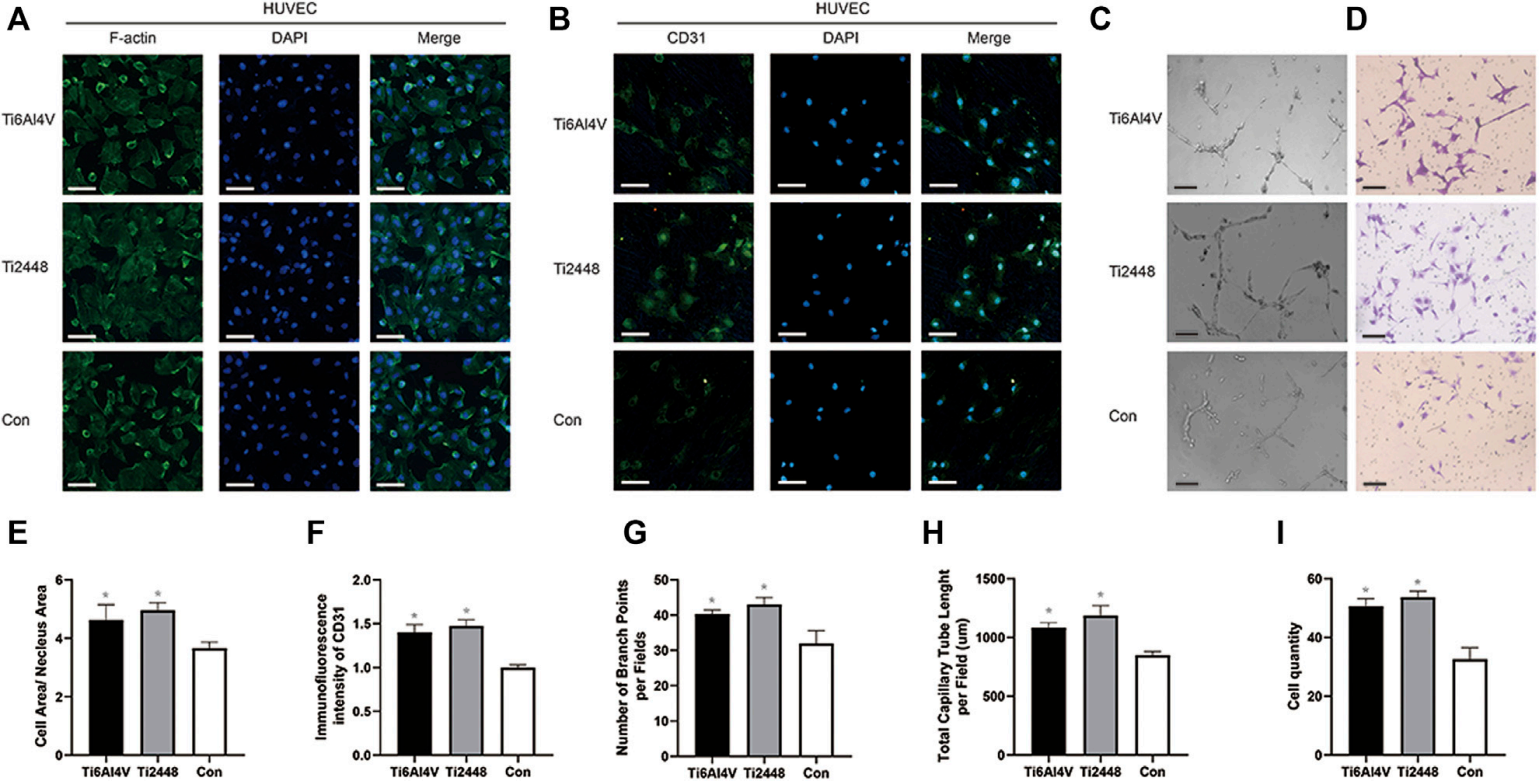
Evaluation of the effects of the different scaffolds on angiogenesis in HUVECs *in vitro*. Immunofluorescence staining for examination of the cytoskeleton and expression of the angiogenic marker CD31 in HUVECs. Scale bar = 100 µm **(A,B)**. Tube formation **(C)** and Transwell **(D)** assays to determine the effect of different scaffolds on HUVECs. Scale bar = 200 µm. Quantitative analysis of the cell area/nuclear area in different groups **(E)**. Quantitative analysis of the fluorescence intensity of CD31 in different groups **(F)**. Quantitative analysis of the number of branch points per field in different groups **(G)**. Quantitative analysis of the total capillary tube length per field in different groups **(H)**. Quantitative analysis of the cell quantity in different groups **(I)**. Note: *p < 0.05 compared to the control.

To evaluate the angiogenic effect of Ti2448 and Ti6Al4V on HUVECs *in vitro*, we observed the morphology of HUVECs using immunofluorescence, and the results showed no significant difference in the morphology of HUVECs on the surface of the two scaffolds, but the ratio of cell area to nucleus area in both groups was higher than that in the control group (Figures 7A,E). Immunofluorescence staining images are shown in Figures 7B,F. No significant difference was observed in the intensity of CD31 staining between the Ti2448 and Ti6Al4V groups (*p* > 0.05), indicating similar levels of CD31 synthesis. However, the expression of CD31 in both groups was higher than that in the control group. Tubule formation and Transwell assays showed no significant difference between Ti2448 and Ti6Al4V in terms of the capability of HUVECs for angiogenesis or invasion (Figures 7C,D, G-I).

**FIGURE 8:**
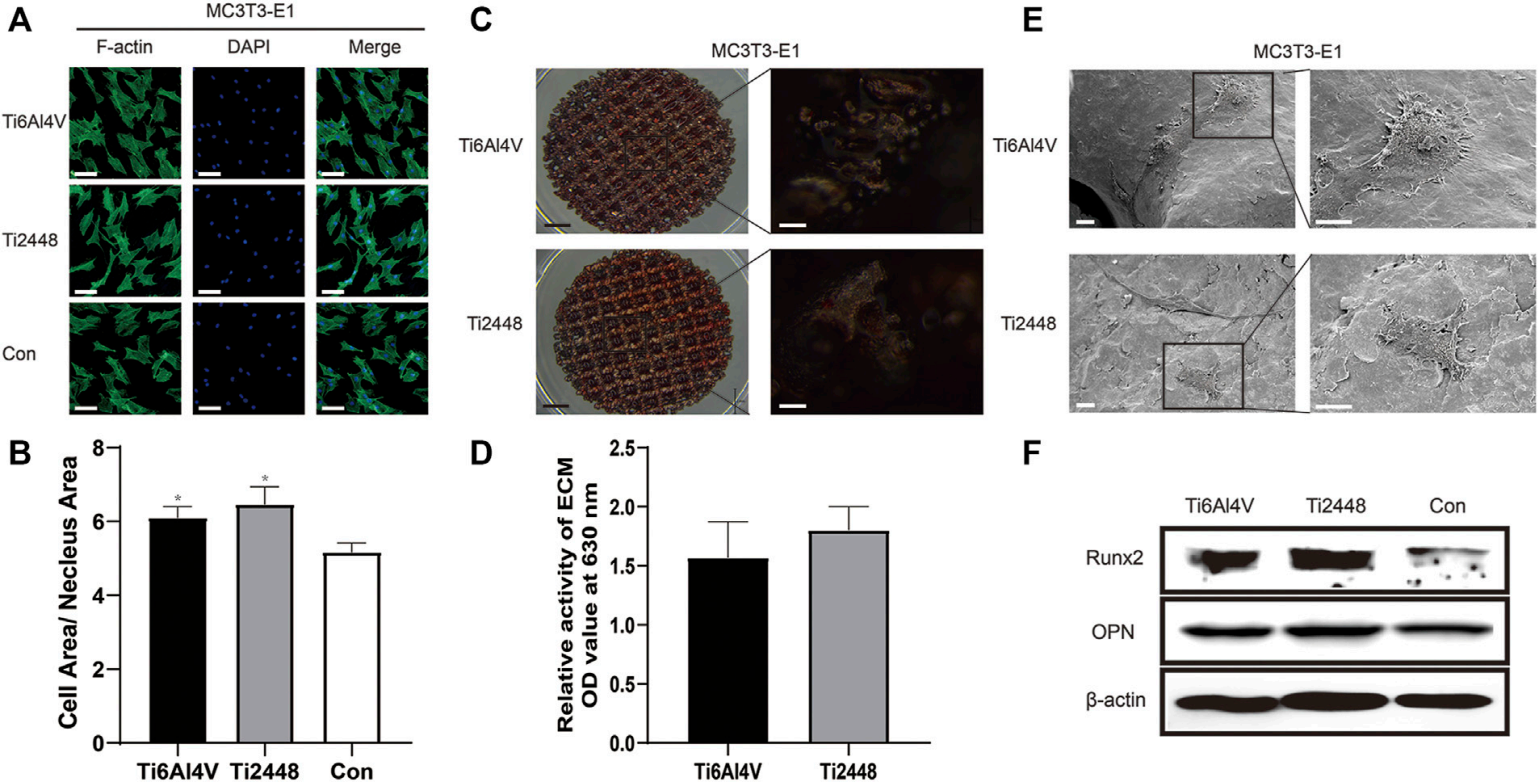
Evaluation of the effects of different scaffolds on osteogenesis in MC3T3-E1 cells *in vitro*. Staining of the cytoskeleton in MC3T3-E1 cells. Scale bar = 100 µm **(A)**. Quantitative analysis of the cell area/nuclear area in different groups **(B)**. Alizarin red staining after 21 days of osteogenic induction to evaluate the effect of different scaffolds on MC3T3-E1 cells. Scale bars: black = 2 mm, white = 100 µm **(C)**. Semiquantitative analysis of mineralization in cells cultured on different scaffolds **(D)**. Observation of the cell morphology on the surface of the scaffolds by SEM. Scale bar = 100 µm **(E)**. Analysis of the expression levels of Runx2 and OPN by western blot **(F)**. Note: *p < 0.05 compared to the control.

The morphology of MC3T3-E1 cells was evaluated and analysed by immunofluorescence staining of the cytoskeleton, as shown in Figure 8A. The results showed that the extent of cell spreading was similar in the Ti2448 and Ti6Al4V groups, with no significant difference in the ratio of the cell area to the nuclear area on the surface, but the ratio of cell area to nucleus area in both groups was higher than that in the control group (Figure 8B). According to the results of alizarin red staining and semiquantitative analysis, the amount of calcium nodule formation on the surface of the scaffold material was similar in the Ti2448 and Ti6Al4V groups, with no significant difference (Figures 8C,D) (*p* > 0.05). As shown in Figure 8E, cells in each group were in a good spreading state on the material, demonstrating relatively high cell activity. The cell surface area and cell spreading state were similar in the two groups. The western blot results showed no significant difference in the expression of OPN or Runx2 between the groups, but the expression of OPN or Runx2 in both groups was higher than that in the control group (Figure 8F).

### Regulation of the Angiogenic Effect on HUVECs and the Osteogenic Effect on MC3T3-E1 Osteoblasts by the Direction of Macrophage Polarization

**FIGURE 9:**
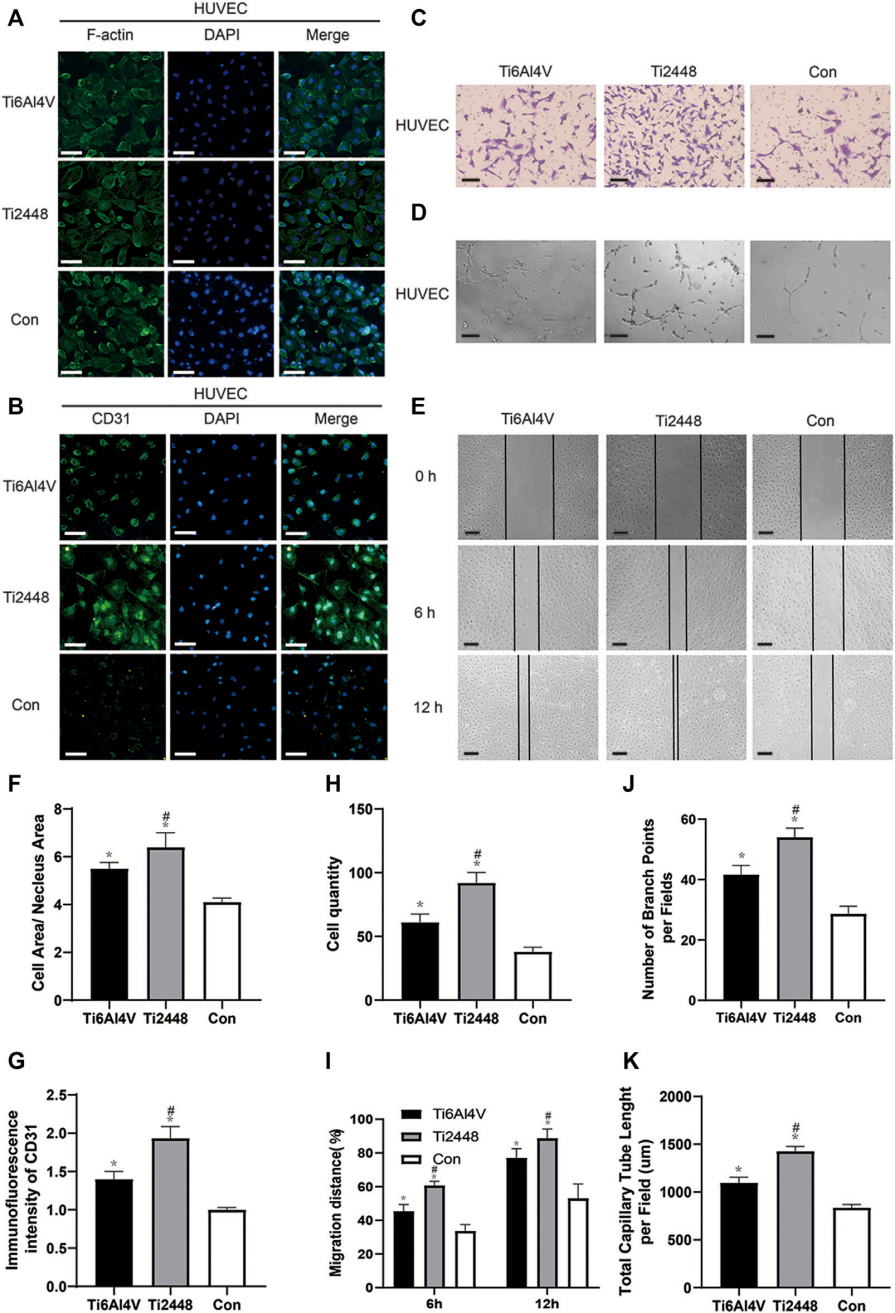
Effects of different scaffolds on the angiogenic effect on HUVECs by regulating the polarization of macrophages. Immunofluorescence staining for examination of the cytoskeleton and expression of the angiogenic marker CD31 in HUVECs. Scale bar = 100 µm **(A,B)**. Representative images of the invasion, tubule formation and migration assays. Scale bar = 200 µm **(C–E)**. Quantitative analysis of the cell area/nuclear area in different groups **(F)**. Quantitative analysis of the fluorescence intensity of CD31 in different groups **(G)**. Quantitative analysis of the cell quantity in different groups **(H)**. Quantitative analysis of the migration distance in different groups **(I)**. Quantitative analysis of the number of branch points per field in different groups **(J)**. Quantitative analysis of the total capillary tube length per field in different groups **(K)**. Note: *p < 0.05 compared to the control; #*p* < 0.05 compared to Ti2448.

The indirect coculture method was used to study the effect of the paracrine activity of the RAW cells induced by Ti2448 and Ti6Al4V on the spreading state of HUVECs. The results are shown in Figure 9A. After 2 days, the cytoplasm-to-nucleus ratio was 6.32 ± 0.31 in the Ti2448 group and 4.56 ± 0.55 in the Ti6Al4V group, with the lowest cytoplasm-to-nucleus ratio in the control group (3.96 ± 0.25) (Figure 9F) (*p* < 0.05). These results suggest that RAW cells can promote the maintenance of a better spreading state of HUVECs after induction. To investigate the effects of different materials on macrophage polarization-mediated angiogenesis, immunofluorescence staining was performed (Figure 9B). The images showed significantly higher CD31 expression on Ti2448 after incubation in CMA than on Ti6Al4V, with the lowest expression in the control group (Figure 9G). Transwell invasion, tubule formation and wound healing assays were used to detect the capability of HUVECs for invasion, angiogenesis and migration under the influence of macrophages, as shown in Figures 9C–E. The results showed better HUVEC invasion, angiogenesis and migration in the Ti2448 group than in the Ti6Al4V group (Figures 9H–K).

**FIGURE 10:**
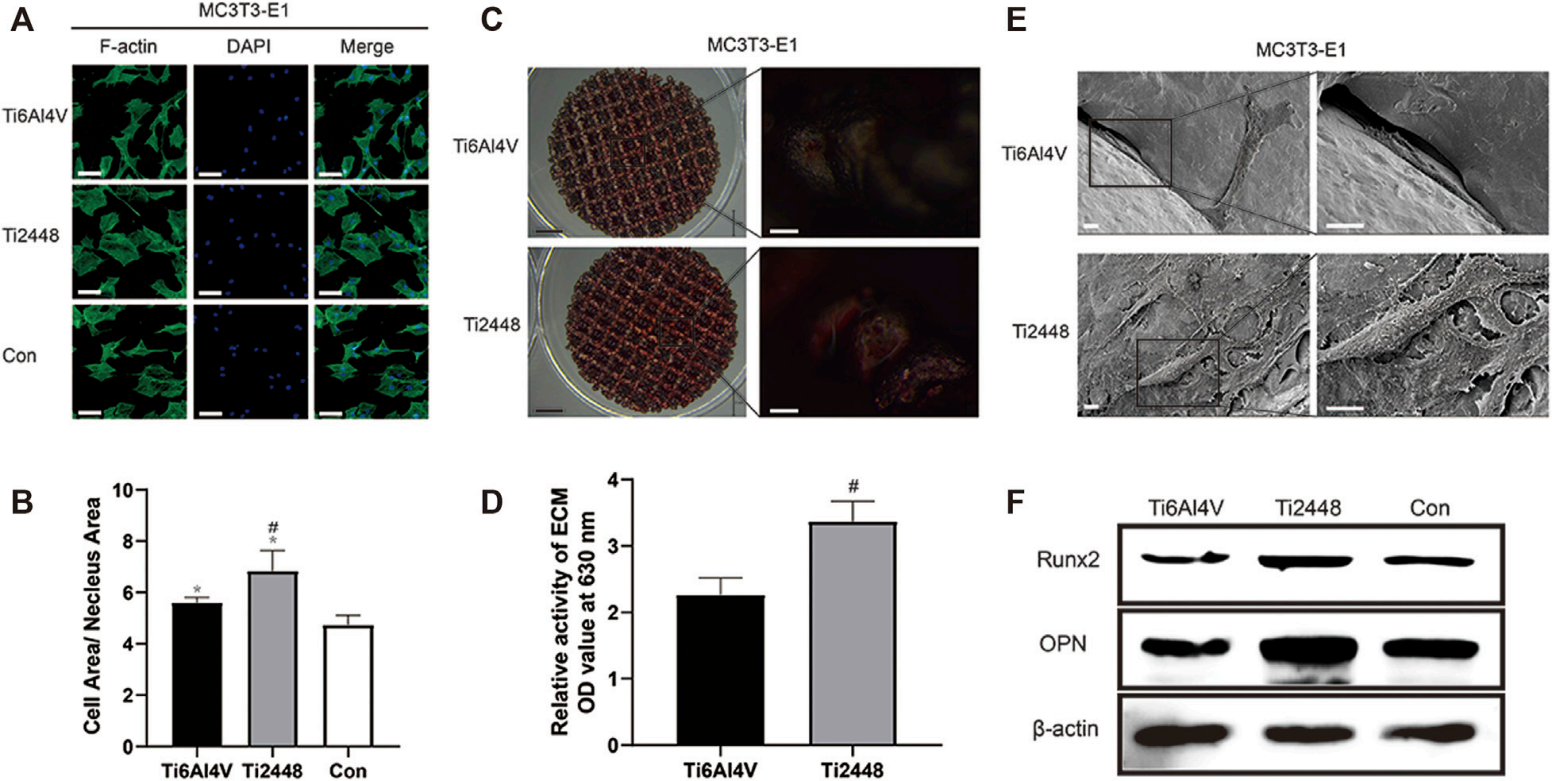
Effects of different scaffolds on the osteogenic effect on MC3T3-E1 cells by regulating the polarization of macrophages. Immunofluorescence staining for examination of the cytoskeleton of MC3T3-E1 cells and evaluation of the cell morphology. Scale bar = 100 µm **(A)**. Quantitative analysis of the cell area/nuclear area in different groups **(B)**. Examination of mineralized nodules on different scaffolds by stereoscopy to evaluate the osteogenic effect. Scale bars: black = 2 mm, white = 100 µm **(C)**. Semiquantitative analysis of mineralization in cells cultured on different scaffolds **(D)**. Observation of the morphology, adhesion and spreading of MC3T3-E1 cells on different scaffold surfaces by SEM. Scale bar = 100 µm **(E)**. Western blot analysis of the expression levels of Runx2 and OPN in different groups **(F)**. Note: *p < 0.05 compared to the control; #*p* < 0.05 compared to Ti2448.

Figure 10A shows the influence of the paracrine activity of RAW cells induced by the different materials on the spreading state of MC3T3-E1 cells. The results showed that the cytoplasm-to-nucleus ratio in the Ti2448 group was 6.87 ± 0.42, which was higher than that in the Ti6Al4V group (5.66 ± 0.35) and control group (4.16 ± 0.37) (Figure 10B) (*p* < 0.05). Alizarin red staining showed a higher number of mineralized nodules on the surface of Ti2448 than on that of Ti6Al4V after 21 days of osteogenic induction. This result suggests that the osteogenic ability of Ti2448 was better than that of Ti6Al4V in the presence of macrophages (Figures 10C,D). SEM showed the same phenomenon, the surface area of MC3T3-E1 cells was greater, the cell spreading condition was better, and more extracellular matrix and filiform pseudopodia were observed in the Ti2448 group, proving that MC3T3-E1 cells in the Ti2448 group had a better tendency to differentiate into osteoblasts (Figure 10E). Western blot analysis showed that the expression levels of the osteogenic-related proteins Runx2 and OPN were higher in the Ti2448 group than in the Ti6Al4V group, with the lowest expression levels in the control group (Figure 10F).

### Regulation of Macrophage Polarization

**FIGURE 11:**
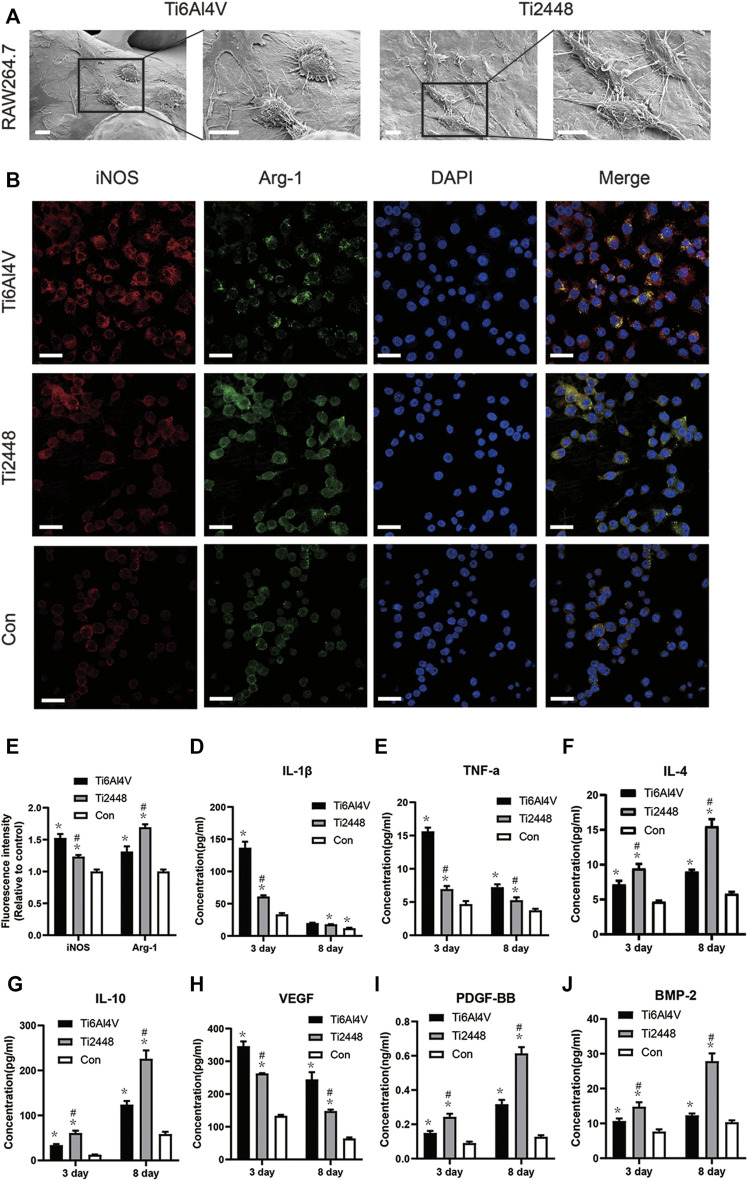
Scaffold stiffness modulates macrophage inflammatory activation. Morphological observation of macrophages on different scaffold surfaces. Scale bar = 100 µm **(A)**. Immunofluorescence staining for iNOS (red, M1 macrophages) and Arg-1 (green, M2 macrophages). Scale bar = 20 µm **(B)**. Quantitative analysis of immunofluorescence intensity **(C)**. Levels of IL-1β and TNF-α secreted by M1 macrophages **(D,E)**, IL-10 and IL-4 secreted by M2 macrophages **(F,G)**, and VEGF, PDGF-BB and BMP-2 **(H–J)** at 3 and 8 days. Note: *p < 0.05 compared to the control; #*p* < 0.05 compared to Ti2448.

SEM images showed that macrophages on Ti6Al4V were spherical, with more filamentous pseudopods. However, most of the macrophages on the surface of Ti2448 were elongated and spindle-shaped (Figure 11A). Immunofluorescence staining for the M2 macrophage marker Arg-1 (green) and M1 macrophage marker iNOS (red) (Figure 11B) showed that the fluorescence intensity of Arg-1 was higher in the Ti2448 group than in the Ti6Al4V group, while the fluorescence intensity of iNOS was higher in the Ti6Al4V group than in the Ti2448 group (Figure 11C). The expression levels of iNOS and Arg-1 in the control group were lower than those in the Ti2448 and Ti6Al4V groups. These results indicate that Ti6Al4V has a relative potential to induce the polarization of macrophages towards the M1 phenotype, while Ti2448 can promote the polarization of macrophages towards the M2 phenotype. As shown in Figure 11D, the level of IL-1β secreted by macrophages in the Ti2448 group and the Ti6Al4V group was lower than that in the control group, and the level in the Ti2448 group was lower than that in the Ti6Al4V group at 3 days (*p* < 0.05). However, there was no significant difference between the Ti2448 and Ti6Al4V groups at 8 days (*p* > 0.05). The TNF-α level in the Ti2448 and Ti6Al4V groups was lower than that in the control group, and the level in the Ti2448 group was significantly lower than that in the Ti6Al4V group at 3 and 8 days (*p* < 0.05) (Figure 11E). The IL-1β and TNF-α levels at 8 days were lower than those at 3 days. The levels of IL-4 and IL-10 secreted by macrophages were lower on Ti6Al4V than on Ti2448 at 3 and 8 days (Figures 11F,G) (*p* < 0.05). The IL-4 and IL-10 levels at 8 days were higher than those at 3 days. The VEGF level was higher in the Ti6Al4V group than in the Ti2448 group at 3 and 8 days, and the VEGF level at 8 days was lower than that at 3 days (*p* < 0.05) (Figure 11H). The PDGF-BB and BMP-2 levels in the Ti2448 group were higher than those in the Ti6Al4V group at 3 and 8 days (Figures 11I,J) (*p* < 0.05), and the PDGF-BB and BMP-2 levels at 8 days were higher than those at 3 days. The iNOS and Arg-1 expression levels and the level of protein secretion from RAW cells were not significantly different between the CMD-Ti6Al4V and CMD-Ti2448 groups (Supplementary Figure S2) (*p* > 0.05).

### Stress Perception of RAW Cells

**FIGURE 12:**
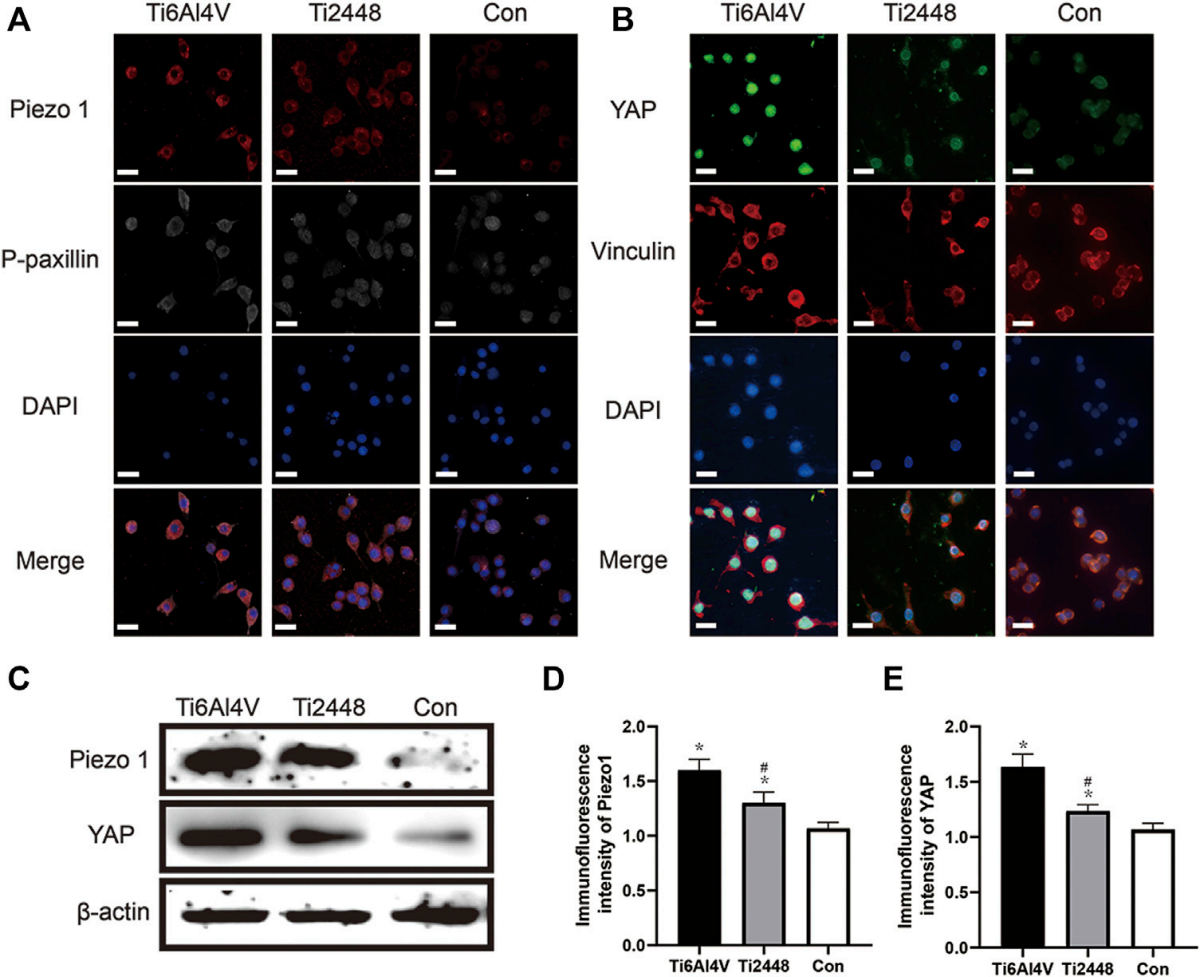
Substrate stiffness regulates Piezo1 expression and YAP nuclear translocation. Piezo1 expression and cell adhesion to the extracellular matrix (focal adhesion) were observed by immunofluorescence. Scale bar = 20 µm **(A)**. Effect of different substrate stiffnesses on YAP localization in macrophages evaluated by immunofluorescence staining. Scale bar = 20 µm **(B)**. Piezo1 and YAP expression in different groups **(C)**. Quantitative analysis of immunofluorescence intensity **(D,E)**. Note: *p < 0.05 compared to the control; #*p* < 0.05 compared to Ti2448.

To explore the perception of RAW cells to materials with different matrix stiffnesses, we seeded the cells onto the surface of different materials and analysed the cytoskeleton and Piezo1 and YAP expression by immunofluorescence (Figures 12A,B). The results showed lower Piezo1 expression on the surface of Ti2448 than on that of Ti6Al4V, and the RAW cells on the surface of Ti2448 were more spindle-shaped (Figures 12A,D). Regarding the influence of adhesion kinetics on the nuclear translocation of YAP, we observed high YAP expression and nuclear localization in the Ti6Al4V group. In contrast, in cells cultured on Ti2448, YAP was still outside of the nucleus, with a lower overall expression level (Figures 12B,E). The levels of Piezo1 and YAP expression were different among the groups, with a higher expression level in the Ti6Al4V group than in the Ti2448 group and the lowest expression level in the control group (Figure 12C).

## Discussion

Recently, the fabrication of porous Ti2448 scaffolds with excellent mechanical properties, good biocompatibility and improved new bone ingrowth has received considerable attention (Vu and Bose, 2020). The surface morphology of the 3D-printed Ti6Al4V and Ti2448 scaffolds was similar. The elemental composition of the two groups of scaffolds was different, with that of the Ti2448 scaffold circumventing potential toxicity. For the two kinds of scaffolds with pore size of 1,000 µm and porosity of 82.63%, the compressive strength of Ti2448 scaffold is higher than that of Ti6Al4V scaffold, while the elastic modulus of Ti2448 scaffold is lower than that of Ti6Al4V scaffold. The elastic modulus of Ti2448 scaffold was similar to that of natural bone (Liu et al., 2018).

Our previous studies demonstrated that a Ti2448 half-pin could enhance osseointegration and reduce pin loosening in the external fixation of tibial fractures in dogs *in vitro* and *in vivo* (Zheng et al., 2011). In this study, we also found that Ti2448 exhibited better angiogenesis and osteogenesis than Ti6Al4V in a rabbit femoral condylar defect model. Excellent osteogenesis and angiogenesis are characterized by the growth of bone and blood vessels around and inside the scaffold, Ca and P deposition, and increased CD31 expression around the scaffold. Recent studies have found that immune regulation is mainly manifested by changes in the state of macrophage polarization and the secretion of cytokines, which constitute the potential mechanism of promoting osteogenesis and angiogenesis (Sadowska and Ginebra, 2020). Subsequently, we generated a subcutaneous implant model and a femoral condylar defect model to explore these potential mechanisms in rabbits. Interestingly, CD68^+^ macrophages are widely distributed in tissues and organs of the body, with high heterogeneity and plasticity. The first stage of bone repair under physiological conditions is the inflammatory stage, and a large number of macrophages are recruited to the implant site (Vasse et al., 2021). In subcutaneous tissue and bone tissue, more CCR7^+^ cells surround Ti6Al4V, and more Arg-1^+^ macrophages surround Ti2448. Macrophages remove cell debris and foreign bodies through the Toll-like receptor pathway and induce an innate immune response (Rabelo et al., 2019). At the same time, mesenchymal stem cells (MSCs) are recruited (He et al., 2019). M2 macrophages around the material promote tissue repair and secrete factors that contribute to angiogenesis and osteogenesis.

The effector cells in which Ti2448 exerts biological effects include osteogenic precursor cells, endothelial cells, and macrophages, among others. We directly seeded MC3T3-E1 cells, HUVECs and RAW cells onto the surface of Ti6Al4V and Ti2448 scaffolds, with different stiffnesses, and the results showed no significant difference in the effect of the two materials on angiogenesis or osteogenesis. However, macrophages responded positively to Ti2448, with low stiffness. Macrophages appear in the initial stage of bone repair and can also be said to be involved in the stage of bone repair initiation (Lopez-Silva et al., 2020). Decreasing the number of macrophages in the initial stage of bone repair and inhibiting the inflammatory response will lead to poor bone repair, suggesting that macrophages play an important role in the whole process of bone repair (Sridharan et al., 2019). The plasticity and importance of macrophages in the process of bone repair make them a potential regulatory target for promoting bone repair (He et al., 2020). Some studies have reported that some angiogenic factors are secreted by macrophages (Ge et al., 2021). For example, M1 macrophages can secrete VEGF and promote the germination and extension of blood vessels (Luo et al., 2021). The secretion of PDGF-BB and BMP-2 from M2 macrophages is conducive to vascular maturation and stability and osteogenesis (Jain et al., 2019). We found that materials with different stiffnesses showed significant differences in macrophage-mediated angiogenesis and osteogenesis by the indirect coculture of macrophages with MC3T3-E1 cells and HUVECs on the material surface. These results further confirmed that the differences in angiogenesis and osteogenesis of Ti2448 *in vivo* and *in vitro* are indirectly caused by macrophages.

Previous studies have shown that the properties or composition of a biomaterial, such as its chemical function, size, shape, and stiffness, can regulate the severity and outcome of the immune response (Li W. et al., 2019). The elastic modulus of an implant can also regulate the capacity of macrophages for phagocytosis and migration (Sridharan et al., 2019). Previous studies have explored the influence of the elastic modulus on the phenotype of macrophages (Chaudhuri et al., 2020). A hard matrix induces the formation of M1 macrophages, while a soft matrix induces the polarization of macrophages towards the M2 phenotype, followed by the corresponding osteogenic and angiogenic effects mediated by M2 macrophages (Jain et al., 2019). Studies have also shown that macrophages adopt their morphology according to their polarization state (Chen et al., 2020). McWhorter et al. showed that the M2 phenotype of mouse bone marrow-derived macrophages (BMDMs) was characterized by cell elongation and spindle-shaped cells expressing M2 markers (Locati et al., 2020). In contrast, macrophages exhibit multiple filopodia and increased adhesiveness in the process of M1 polarization (McWhorter et al., 2013). Congruent with the above findings, we found evidence for the M2 polarization of macrophages on Ti2448, with a low elastic modulus, and the M1 polarization of macrophages on Ti6Al4V, with a high elastic modulus. We observed that macrophages on the surface of Ti2448 showed elongation and increased Arg-1 expression, while those on the surface of Ti6Al4V showed more filopodia and increased iNOS expression. In addition, macrophages on the surface of Ti6Al4V secreted more IL-1β, TNF-α, and VEGF, which are conducive to promoting inflammation and blood vessel germination, while macrophages on the surface of Ti2448 secreted more anti-inflammatory factors, such as IL-4 and IL-10, as well as PDGF-BB and BMP-2, which are conducive to vascular stability and osteogenesis.

Macrophages receive mechanical signals from the extracellular environment, which are then converted through mechanical transduction into biochemical signals, ultimately leading to a series of behavioural and gene-level changes (Hasegawa et al., 2021). However, the molecular mechanism underlying this process remains unclear, and possible mechanisms may involve integrins, podosome-type adhesions (PTAs) and Piezo1 (Coughlin et al., 2021). Piezo-type mechanosensitive ion channel component 1 (Piezo1) has been proved to be a key sensor in the mechanical microenvironment and is widely expressed in non-sensory tissues and cells under physiological conditions (Solis et al., 2019). Cells can sense mechanical stimuli from the microenvironment and manifest as YAP activity levels, and manipulation of YAP levels can determine cell behavior, and YAP-mediated mechanotransduction tunes the macrophage inflammatory response (Dupont et al., 2011). This study revealed the role of Piezo1/YAP signaling axis in the perception of material stiffness by macrophages. To explore the perception by macrophages of titanium alloy materials with different stiffnesses, we carried out immunofluorescence staining and western blot on macrophages cultured on the surface of these materials. Macrophages on the surface of Ti6Al4V showed high Piezo1 and YAP expression, with nuclear YAP localization, while macrophages on the surface of Ti2448 showed more cytoplasmic YAP localization. Results showed that there was no significant difference in the expression levels of P-paxillin and Vinculin in macrophages on Ti2448 and Ti6Al4V. This work shows that titanium alloy implants may modulate the macrophage inflammatory response through Piezo1/YAP signaling axis, thereby regulating angiogenesis and osteogenesis.

In summary, our results indicate that macrophages may adjust their polarization state and function according to the stiffness of the titanium alloy surface, suggesting that biomaterials designed with a low elastic modulus may be beneficial for modulating the remodelling response after implantation. The response of macrophages to the matrix stiffness has been studied in terms of several aspects of different cell phenotypes (Locati et al., 2020). To our knowledge, our study is the first to investigate the effect of the matrix stiffness of titanium alloys on the polarization state of macrophages. Therefore, revealing the role of the biophysical and biochemical properties of titanium alloys in regulating the macrophage response will be helpful for understanding their underlying mechanisms in osteogenesis and angiogenesis.

## Conclusion

This study demonstrates that macrophage polarization plays an important role in angiogenesis and osteogenesis in relation to the stiffness of titanium alloys. The direction of macrophage polarization is modulated by the design of the titanium alloy, with a low elastic modulus promoting angiogenesis *in vivo* and *in vitro* and thereby promoting bone formation. In addition, this study confirms that low-elastic-modulus Ti2448 has no direct or significant effect on angiogenesis and osteogenesis but can promote angiogenesis and osteogenesis by regulating macrophage polarization. This study not only provides guidance for the regulation of M1/M2 macrophage polarization after prosthesis implantation to promote angiogenesis and thus promote osseointegration but also provides a theoretical basis for clinical application.

## Data Availability

The original contributions presented in the study are included in the article/Supplementary Material, further inquiries can be directed to the corresponding authors.

## Supplementary Material

Supplementary Figure S1 An indirect coculture of macrophages with MC3T3-E1 cells and HUVECs on the material surface.

Supplementary Figure S2 The influence of the different compositions of the Ti6Al4V and Ti2448 scaffolds on the regulation of macrophage polarization. Ti6Al4V and Ti2448 scaffolds were immersed in DMEM for 3 days to prepare extracts, which were saved as conditioned medium D (CMD, denoted as CMD-Ti6Al4V and CMD-Ti2448). The expression of iNOS and Arg-1 was detected by immunofluorescence **(A,C)**. Western blotting was used to analyse the expression of iNOS and Arg-1 **(B)**. Levels of cytokines secreted by macrophages **(D–J)**.
