# Supplementary figures and images for: Three-Dimensionally Printed Ti2448 With Low Stiffness Enhanced Angiogenesis and Osteogenesis by Regulating Macrophage Polarization via Piezo1/YAP Signaling Axis
Source: Front Cell Dev Biol. 2021 Nov 15;9:750948. doi: 10.3389/fcell.2021.750948 (PMC8634253; doi:10.3389/fcell.2021.750948)

## Slide 1
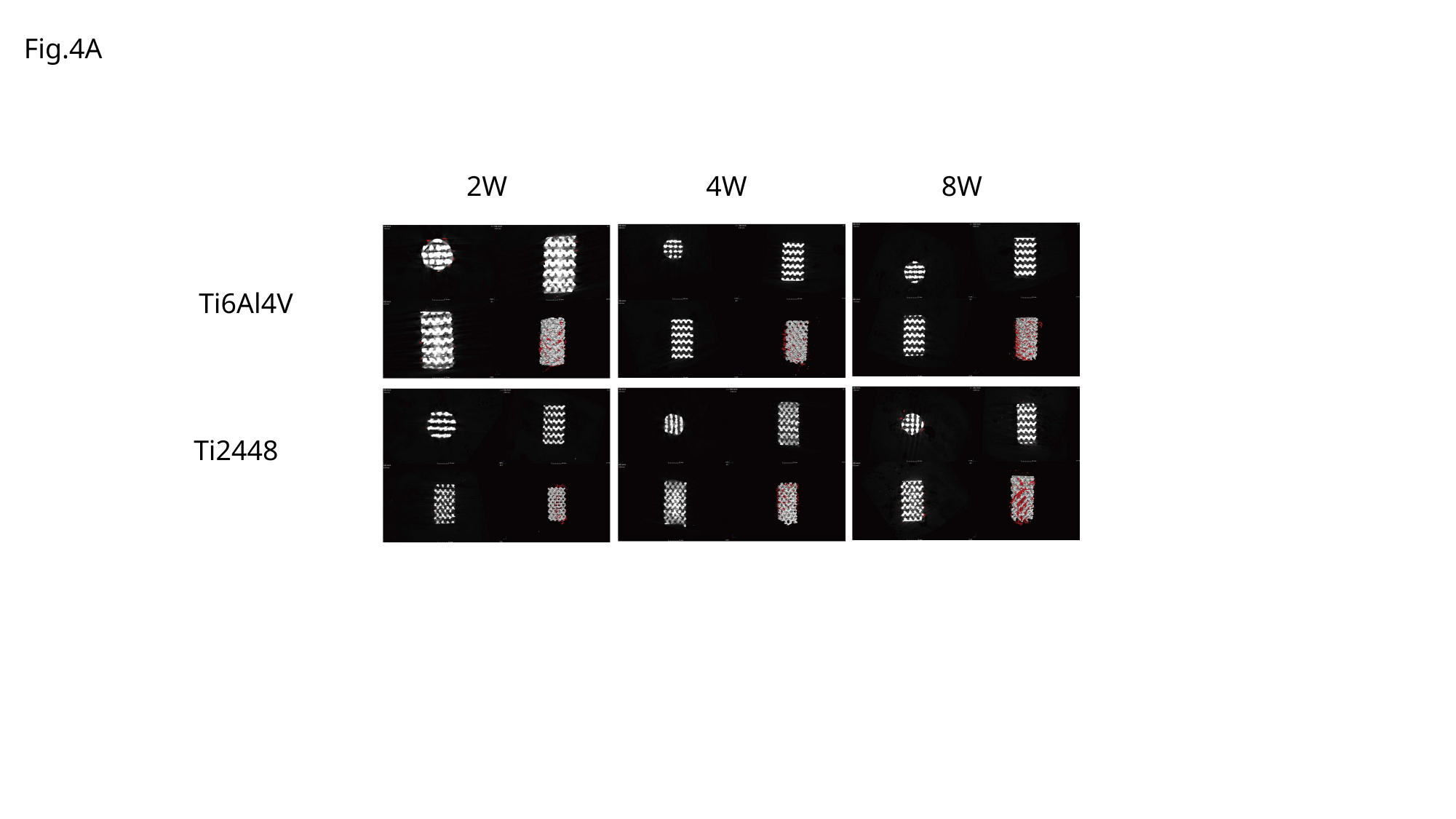

Fig.4A
2W
4W
8W
Ti6Al4V
Ti2448

## Slide 2
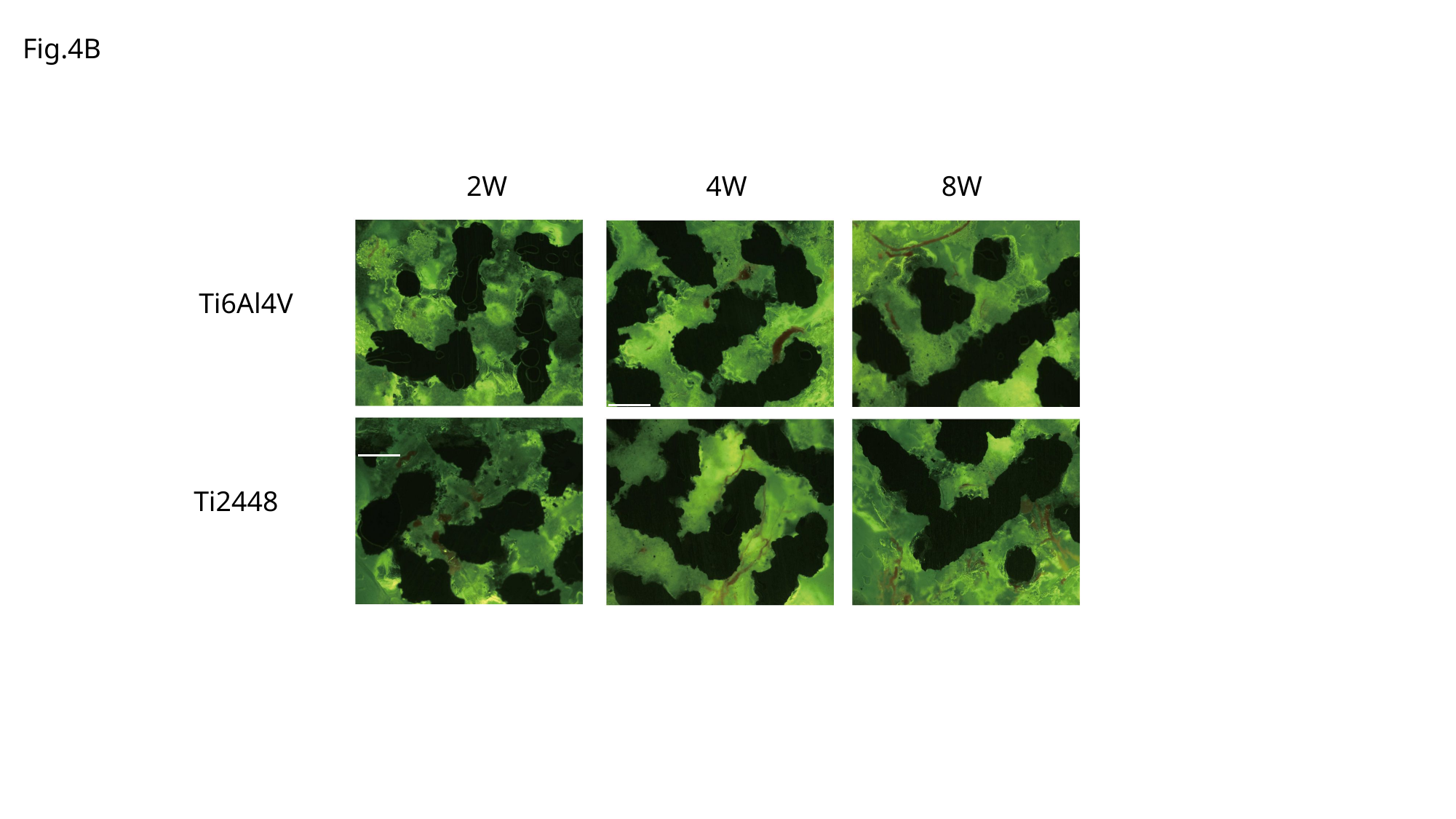

Fig.4B
2W
4W
8W
Ti6Al4V
Ti2448

Supplement: Supplementary file 1 [file DataSheet3.zip › Raw data of angiogenesis in vivo/Raw data of angiogenesis in vivo.pptx]

## Slide 1
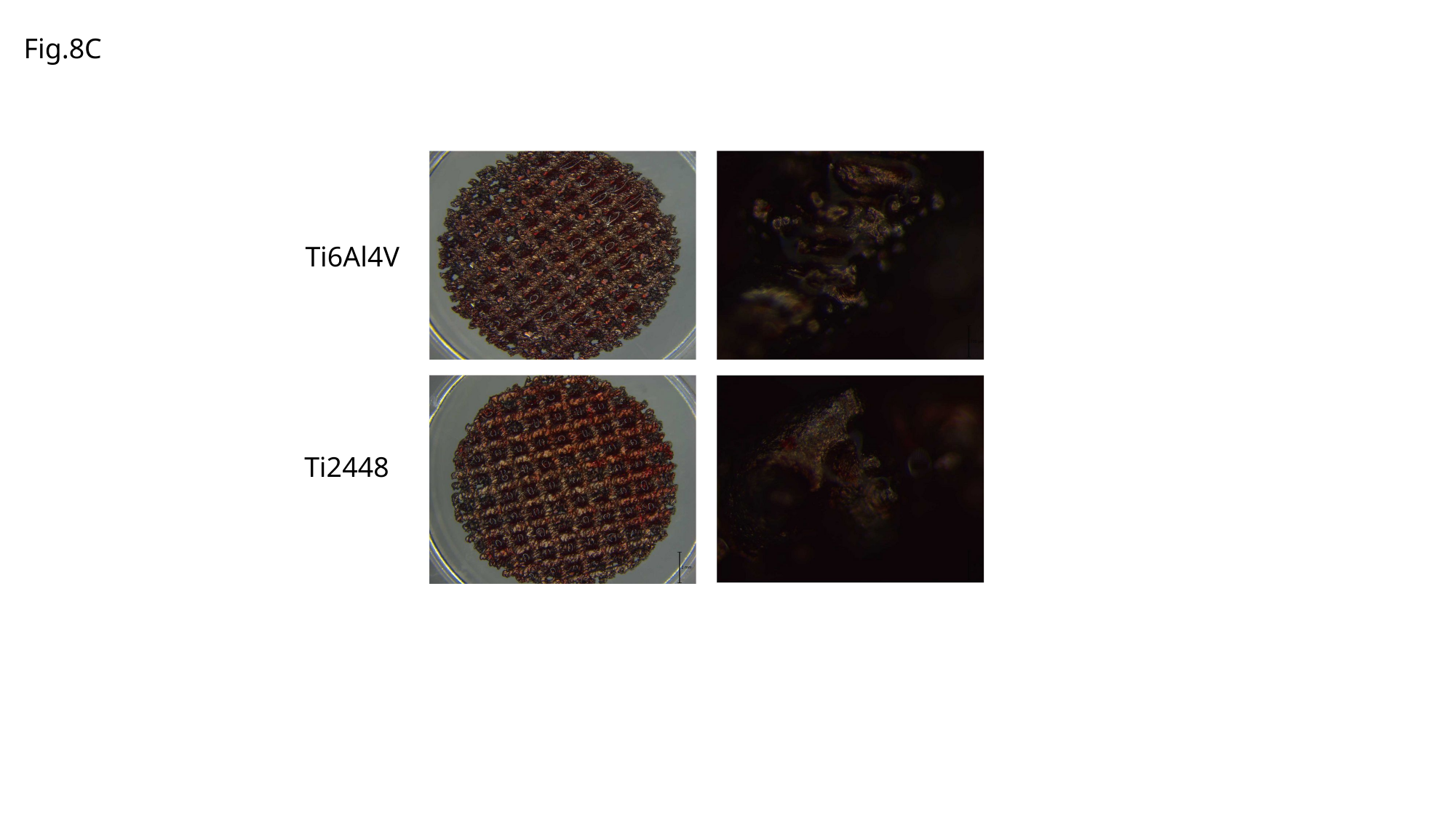

Fig.8C
Ti6Al4V
Ti2448

## Slide 2
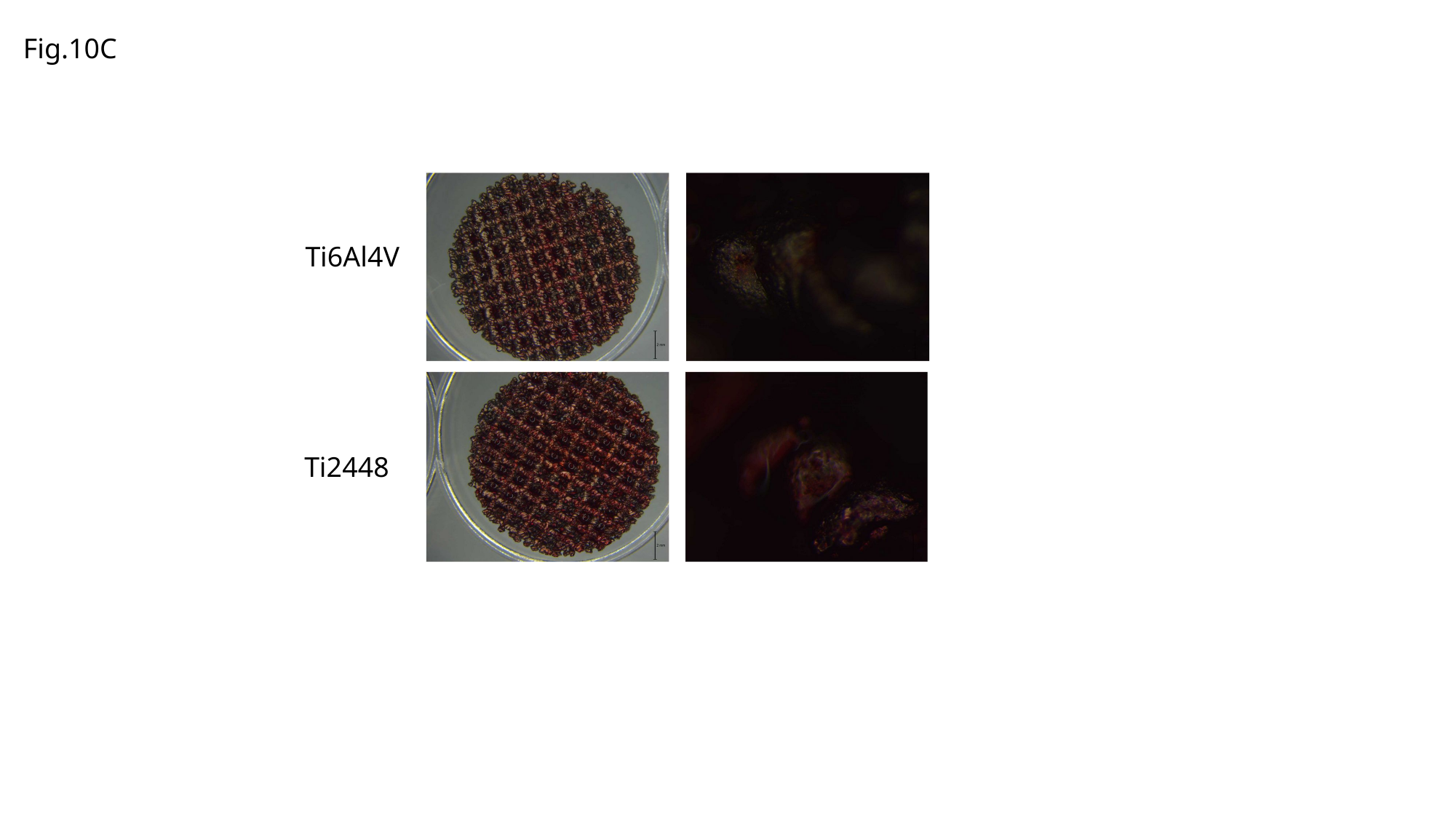

Fig.10C
Ti6Al4V
Ti2448

Supplement: Supplementary file 3 [file DataSheet8.zip › Raw data of osteogenesis in vitro/Raw data of osteogenesis in vitro.pptx]

## Slide 1
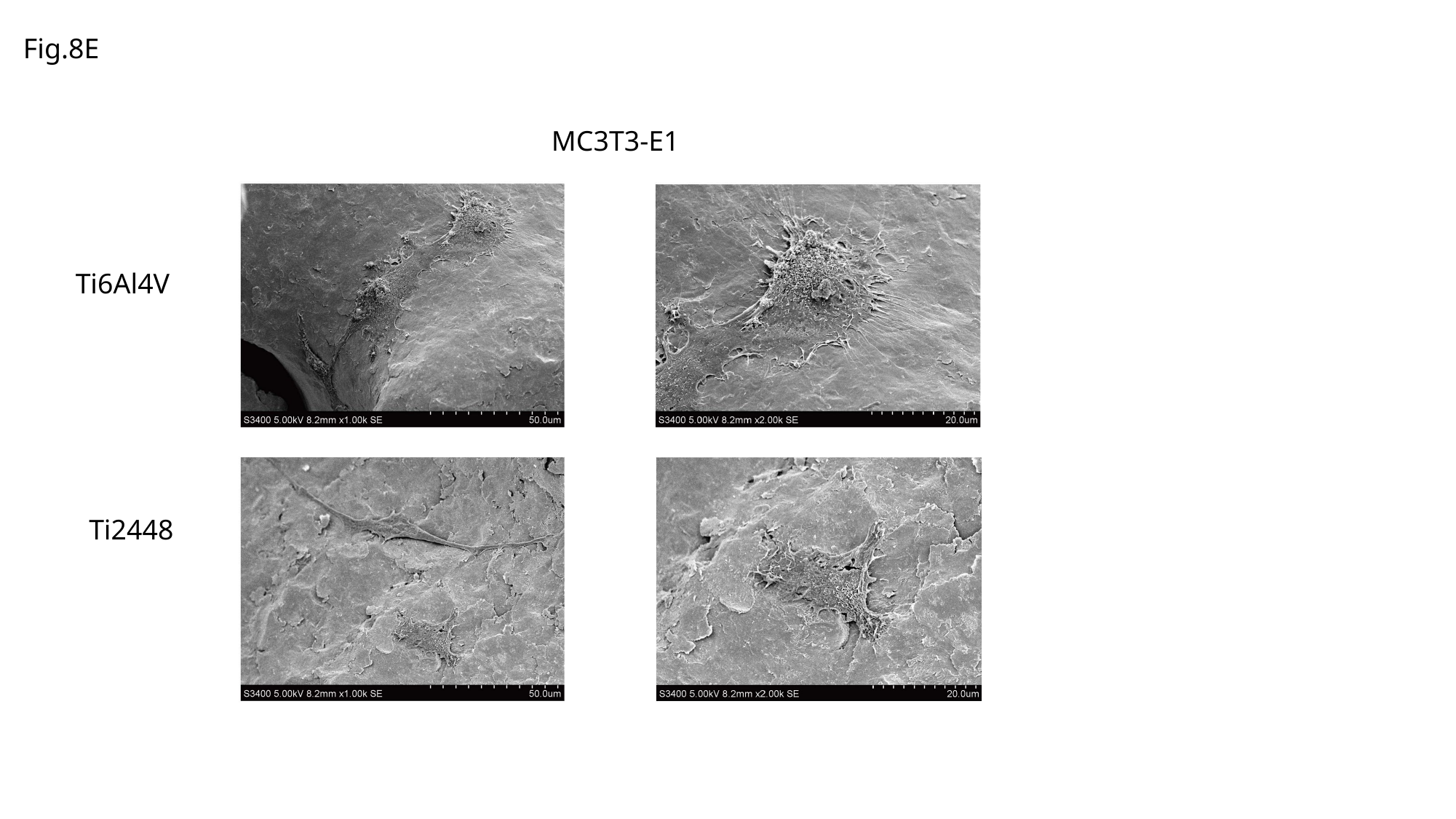

Fig.8E
MC3T3-E1
Ti6Al4V
Ti2448

## Slide 2
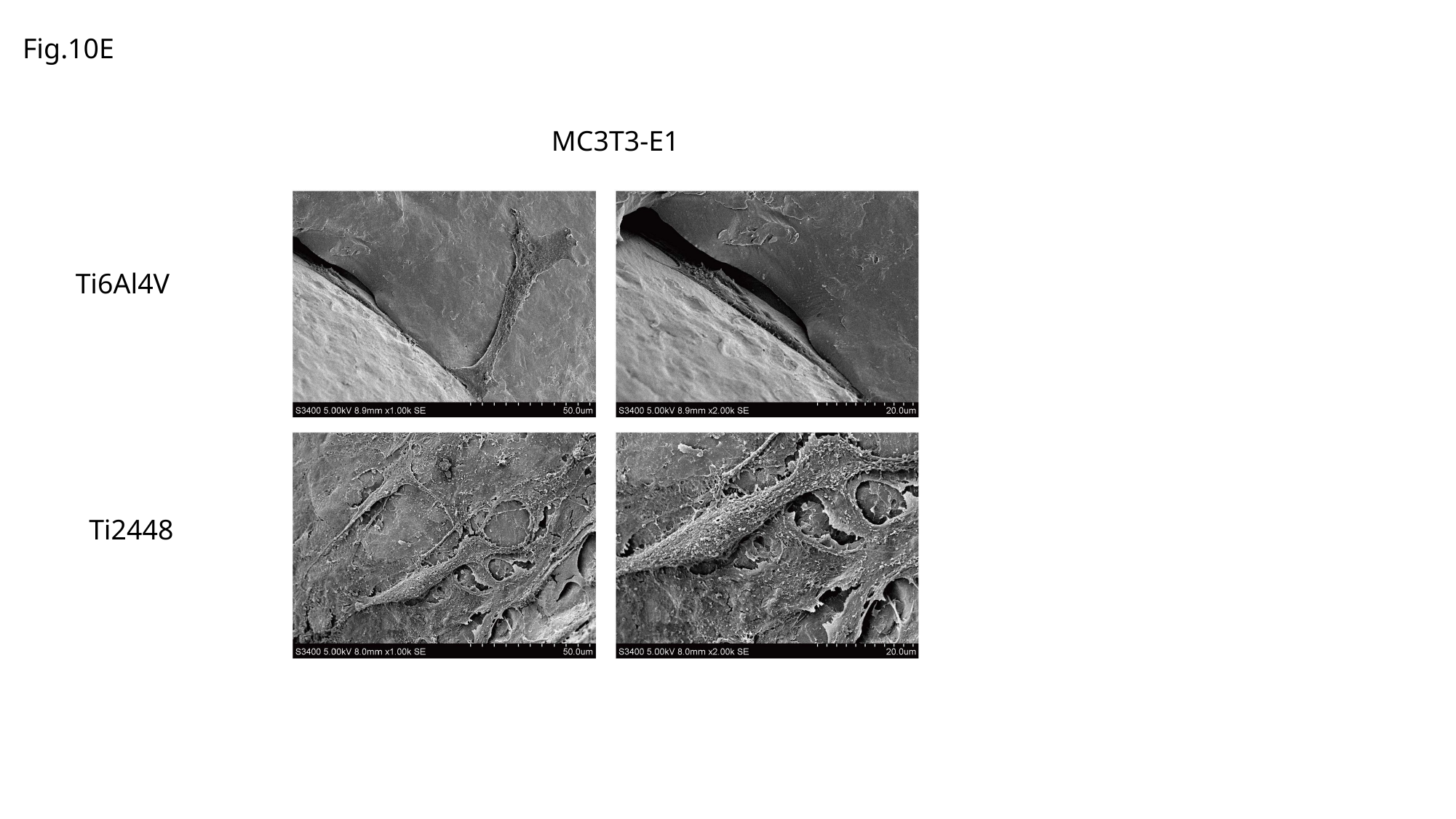

Fig.10E
MC3T3-E1
Ti6Al4V
Ti2448

## Slide 3
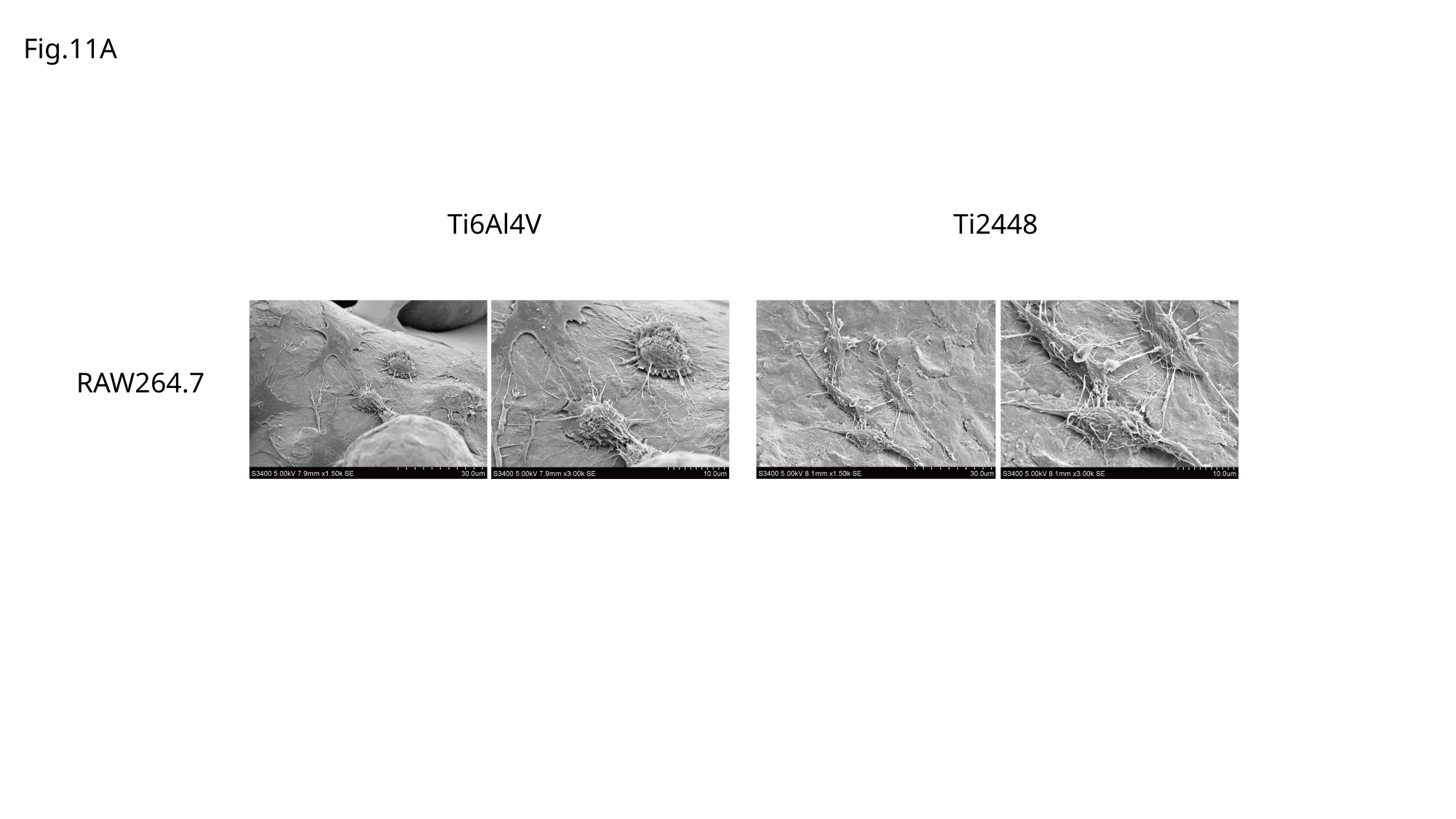

Fig.11A
Ti6Al4V
Ti2448
RAW264.7

Supplement: Supplementary file 6 [file DataSheet1.zip › Raw data of scanning electron microscope/Raw data of scanning electron microscope .pptx]

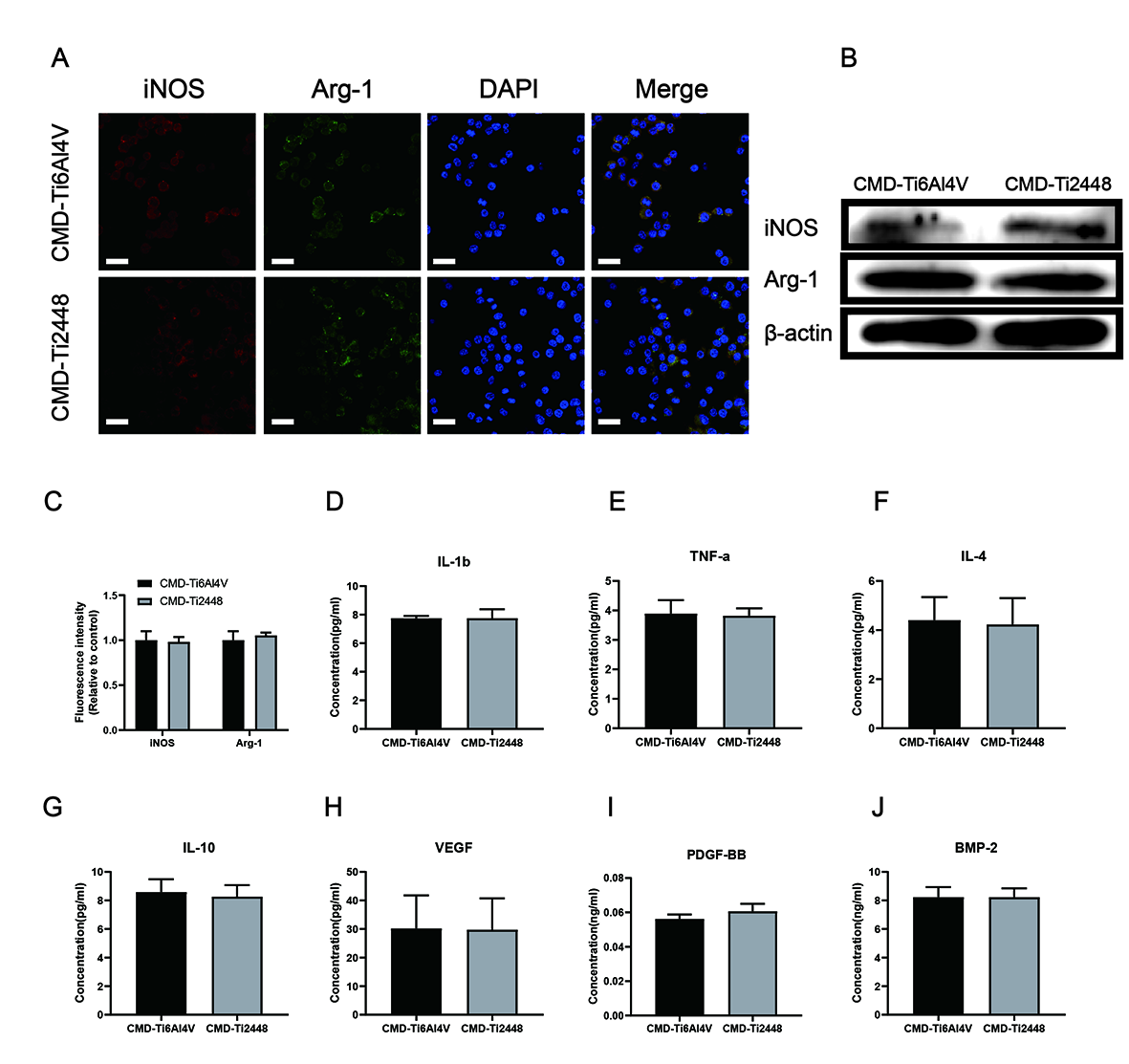

Supplement: Supplementary file 7 [file Image2.TIF]

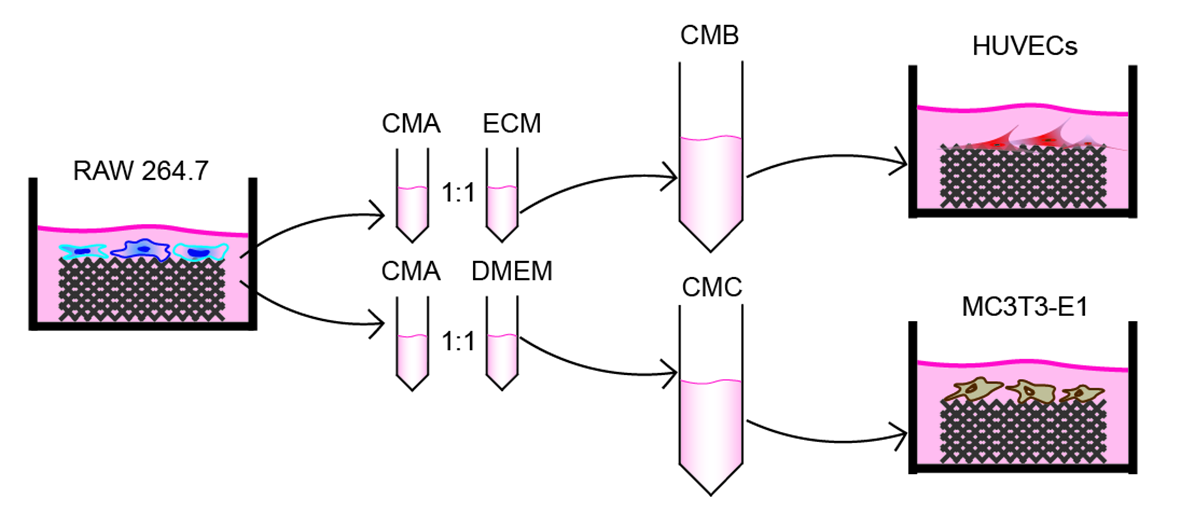

Supplement: Supplementary file 8 [file Image1.TIF]

## Slide 1
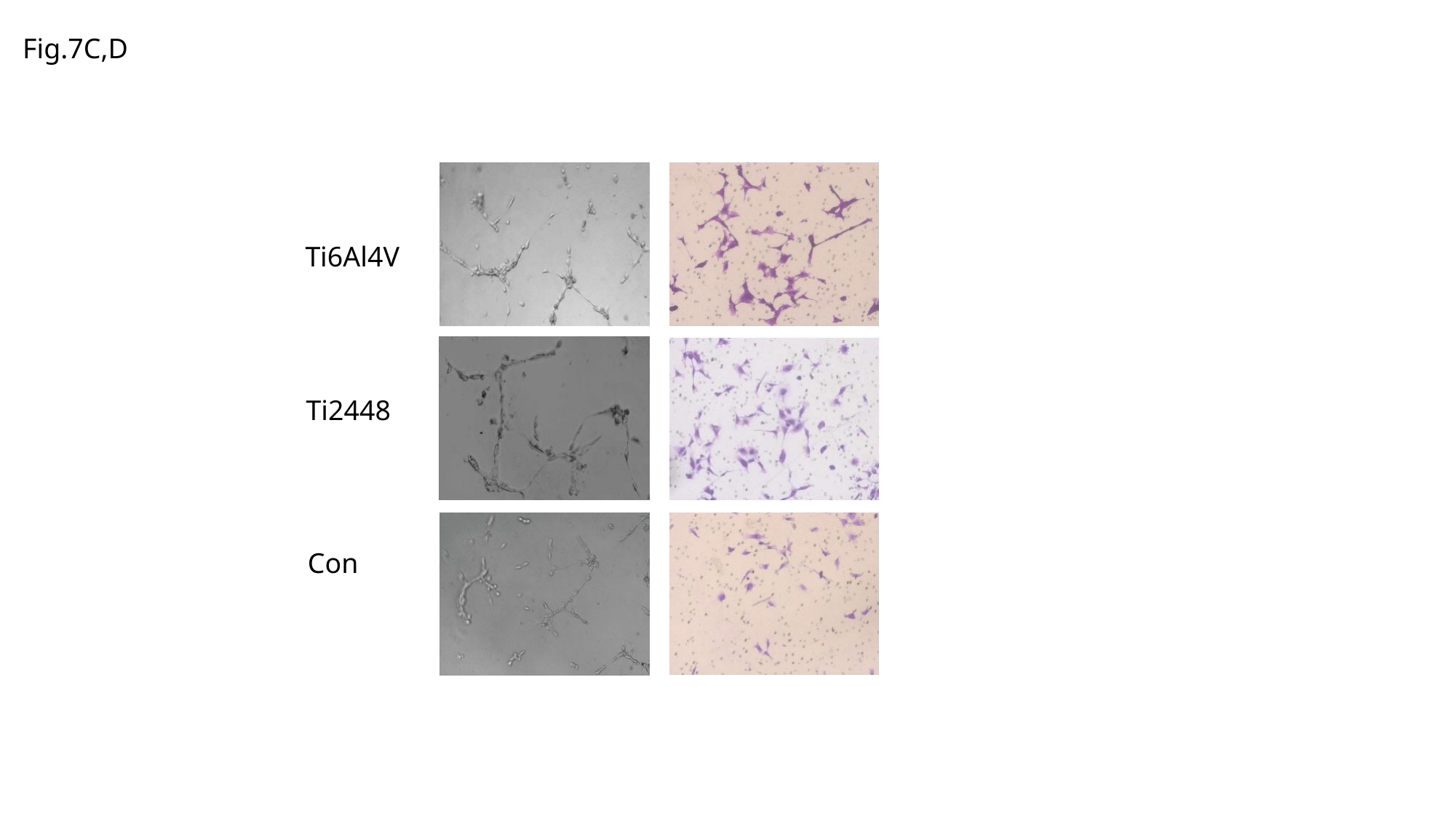

Fig.7C,D
Ti6Al4V
Ti2448
Con

## Slide 2
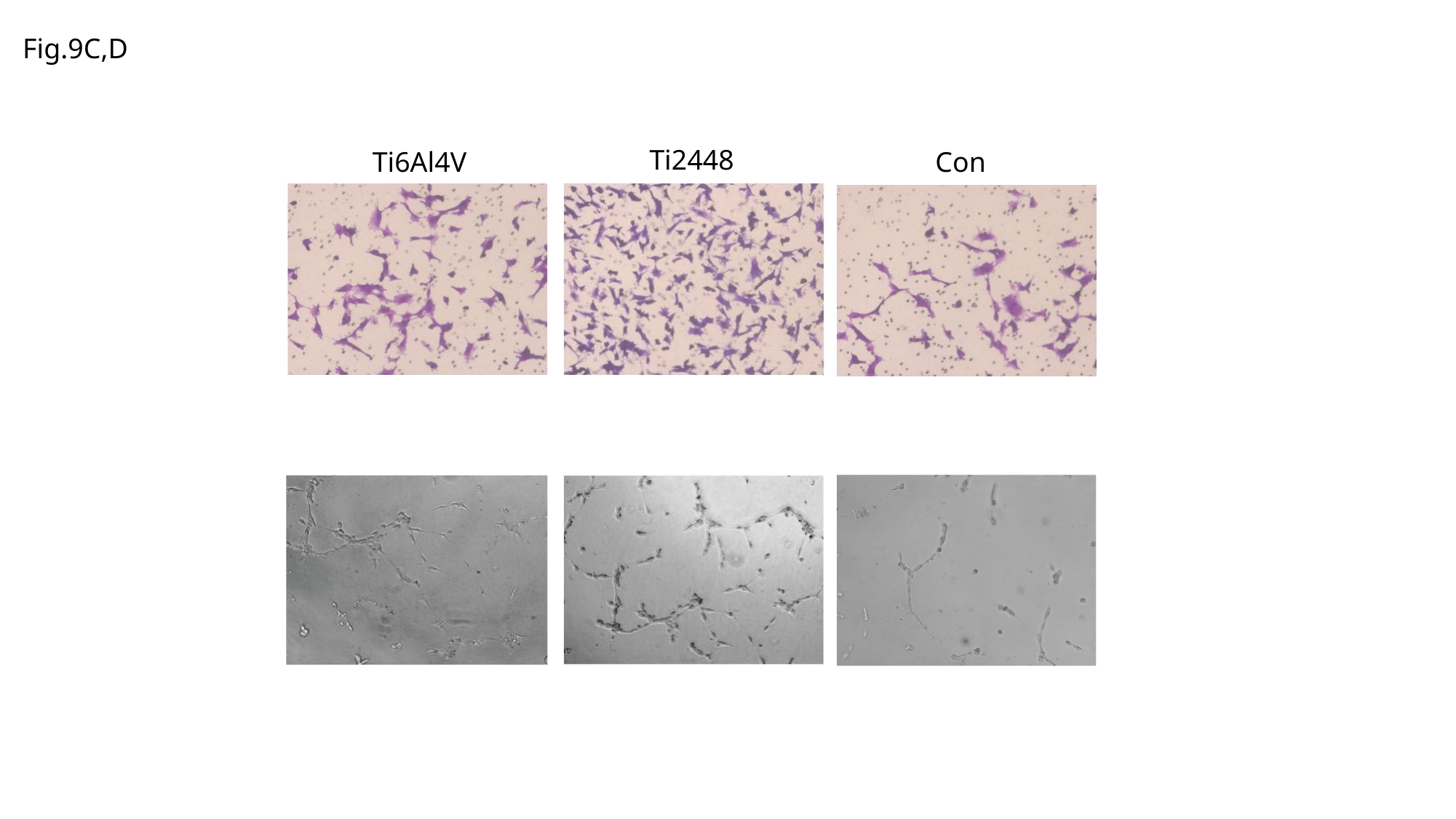

Fig.9C,D
Ti2448
Ti6Al4V
Con

## Slide 3
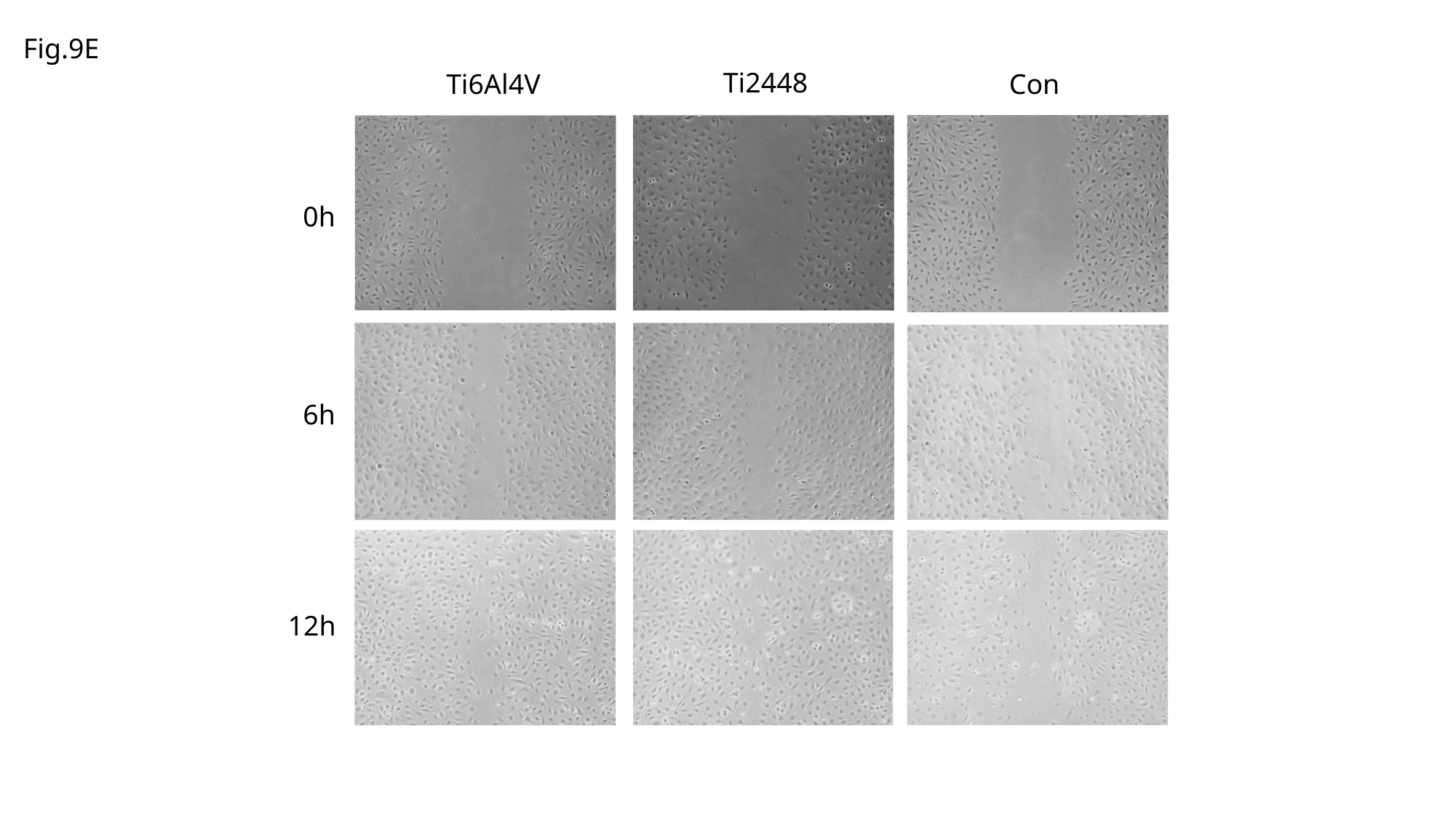

Fig.9E
Ti2448
Ti6Al4V
Con
0h
6h
12h

Supplement: Supplementary file 11 [file DataSheet2.zip › Raw data of angiogenesis in vitro/Raw data of angiogenesis in vitro.pptx]

## Slide 1
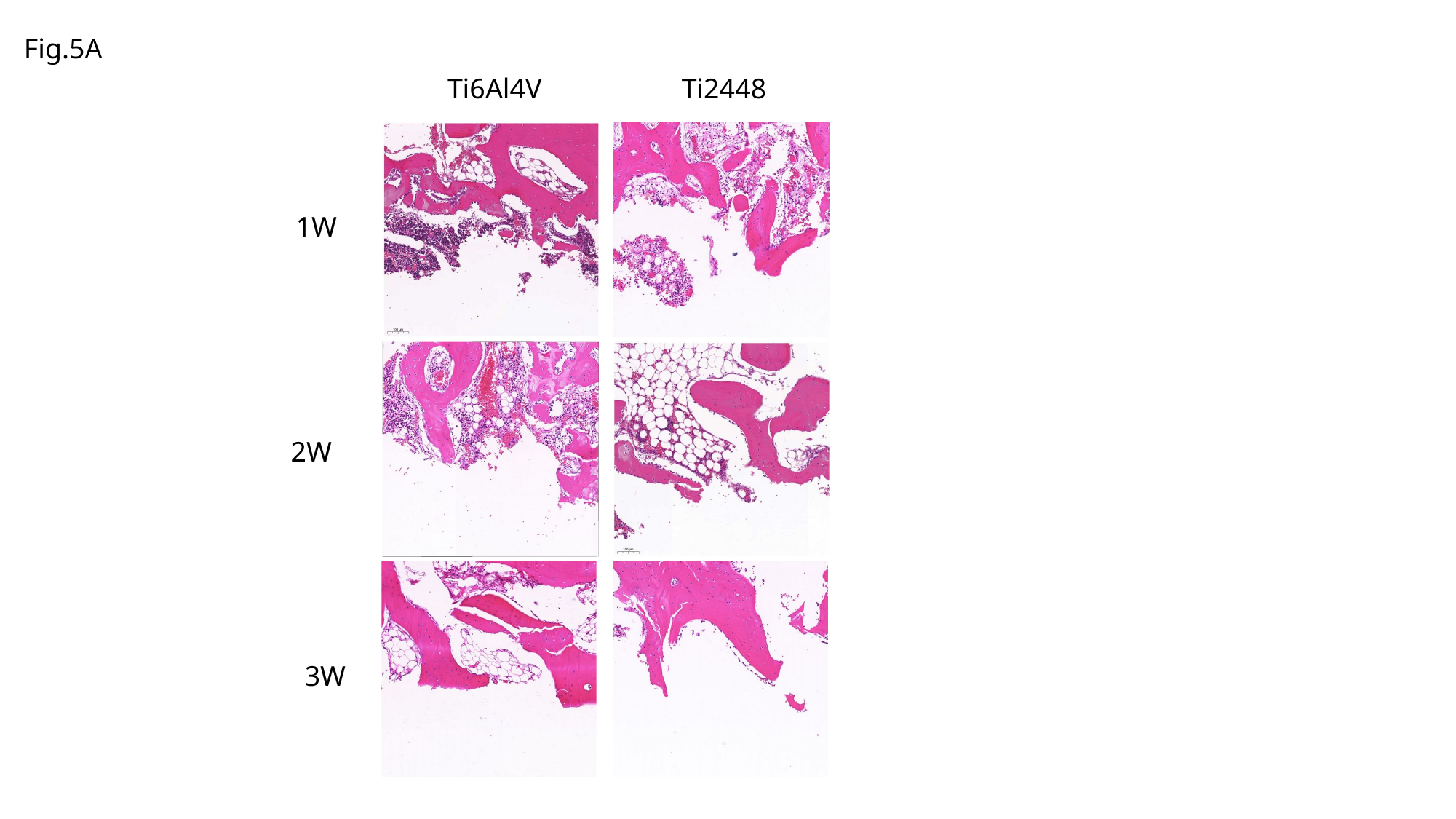

Fig.5A
Ti6Al4V
Ti2448
1W
2W
3W

## Slide 2
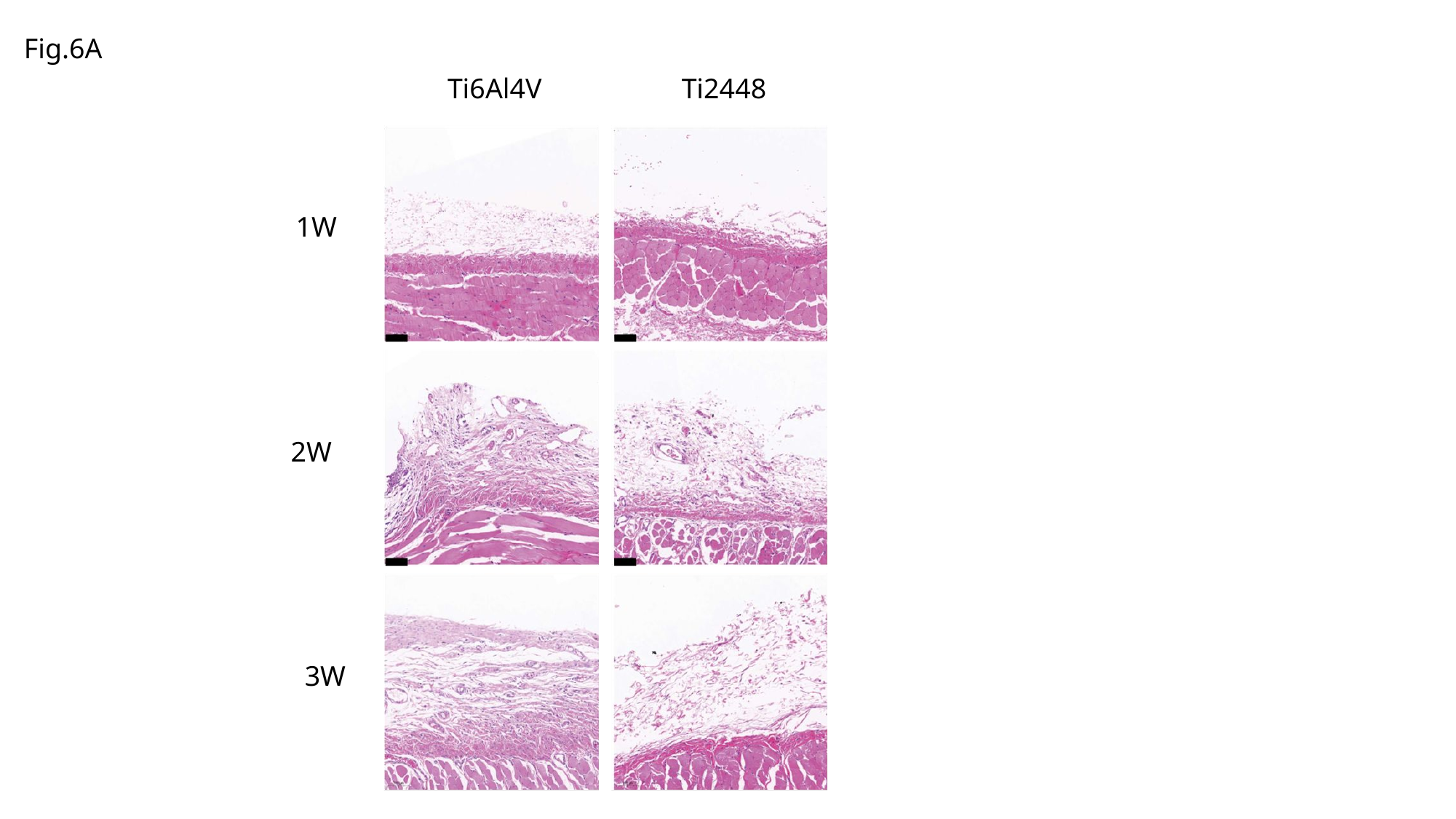

Fig.6A
Ti6Al4V
Ti2448
1W
2W
3W

Supplement: Supplementary file 12 [file DataSheet5.zip › Raw data of H&E/Raw data of H&E.pptx]
