# Supplementary material for: Three-Dimensionally Printed Ti2448 With Low Stiffness Enhanced Angiogenesis and Osteogenesis by Regulating Macrophage Polarization via Piezo1/YAP Signaling Axis
Source: Front Cell Dev Biol. 2021 Nov 15;9:750948. doi: 10.3389/fcell.2021.750948 (PMC8634253; doi:10.3389/fcell.2021.750948)
Supplement: Supplementary file 2 [file DataSheet11.zip › Raw data of western blot/Raw data of western blot.pptx]

## Slide 1
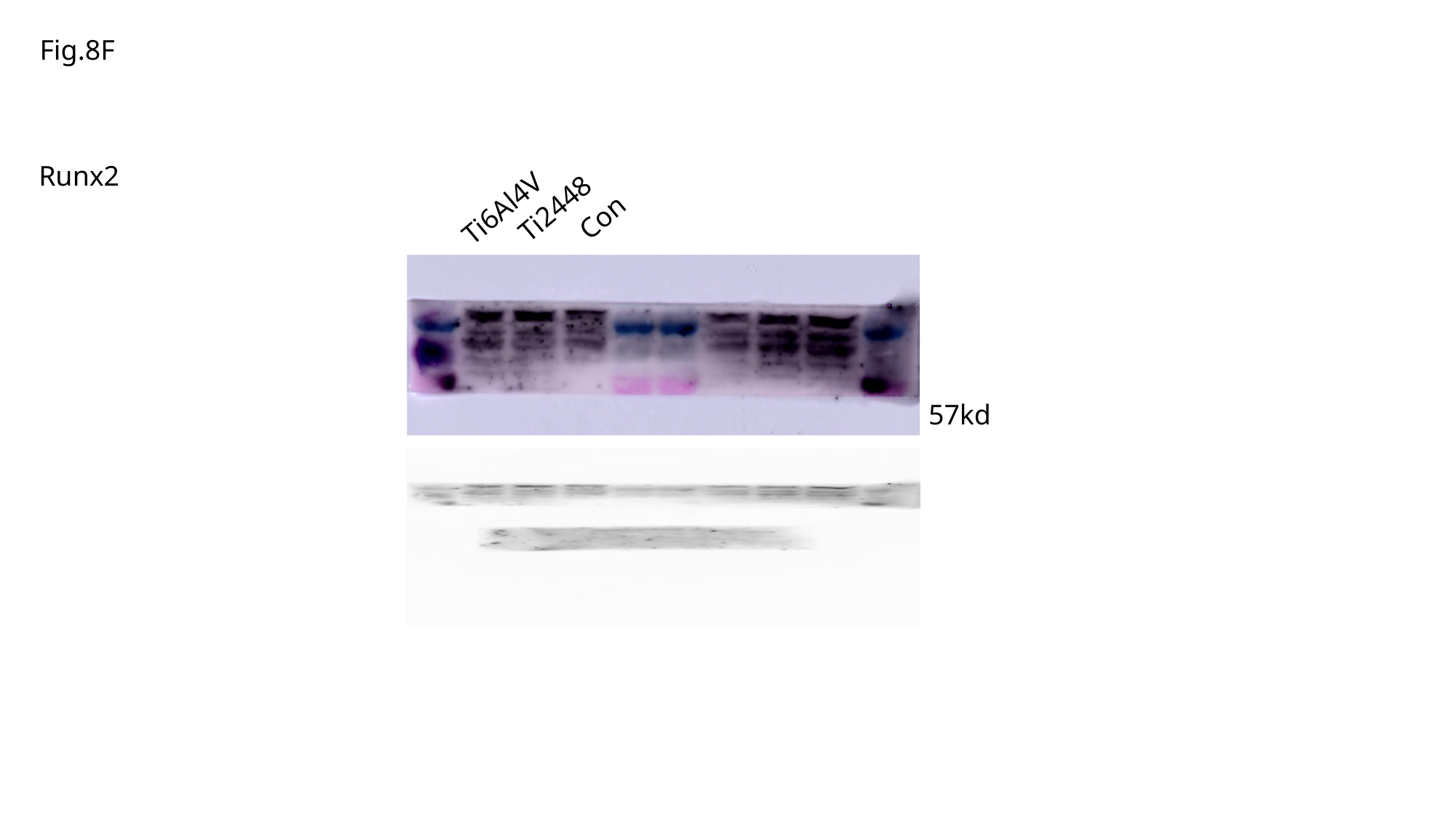

Fig.8F
Runx2
Ti6Al4V
Ti2448
Con
57kd

## Slide 2
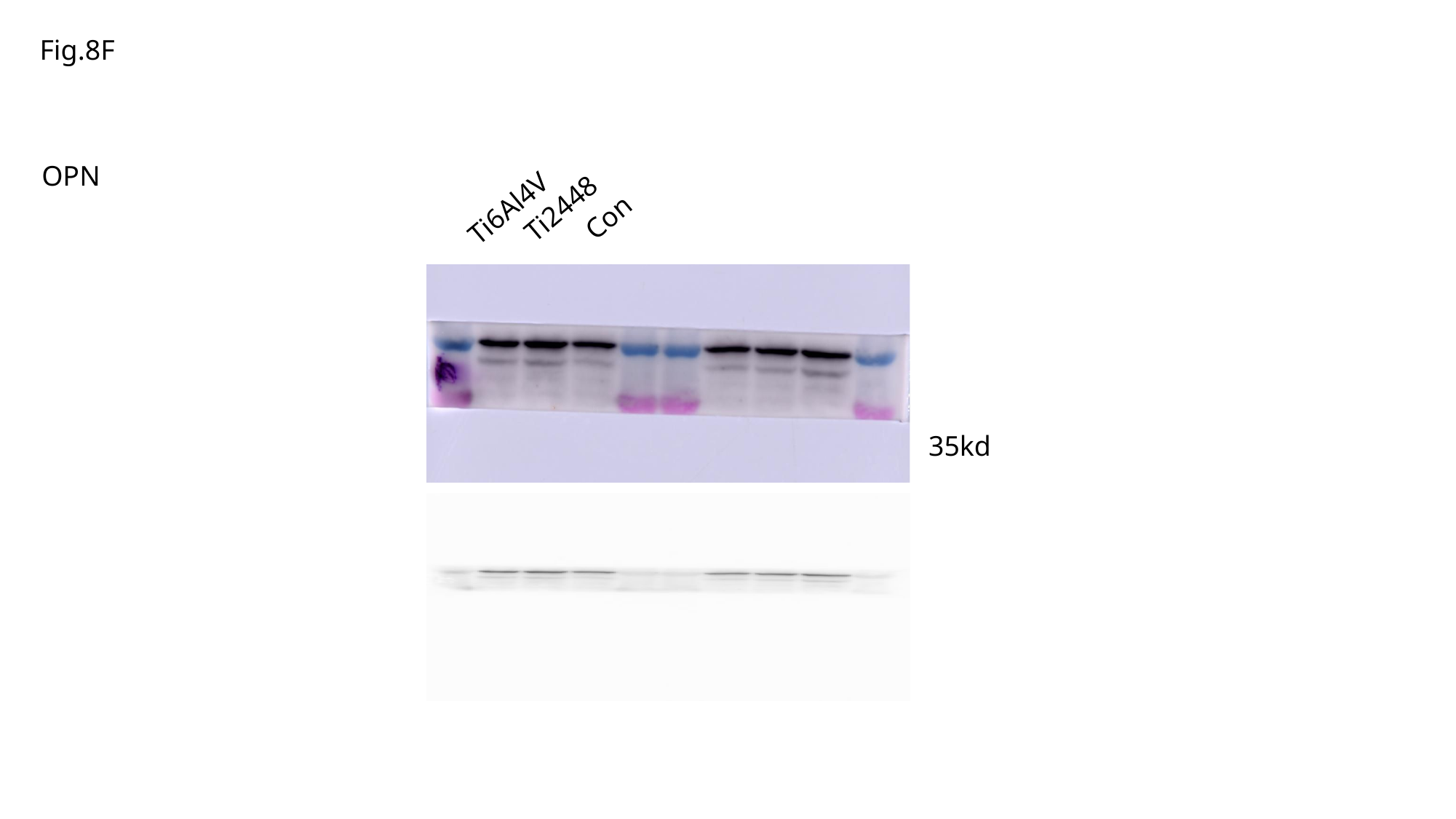

Fig.8F
OPN
Ti6Al4V
Ti2448
Con
35kd

## Slide 3
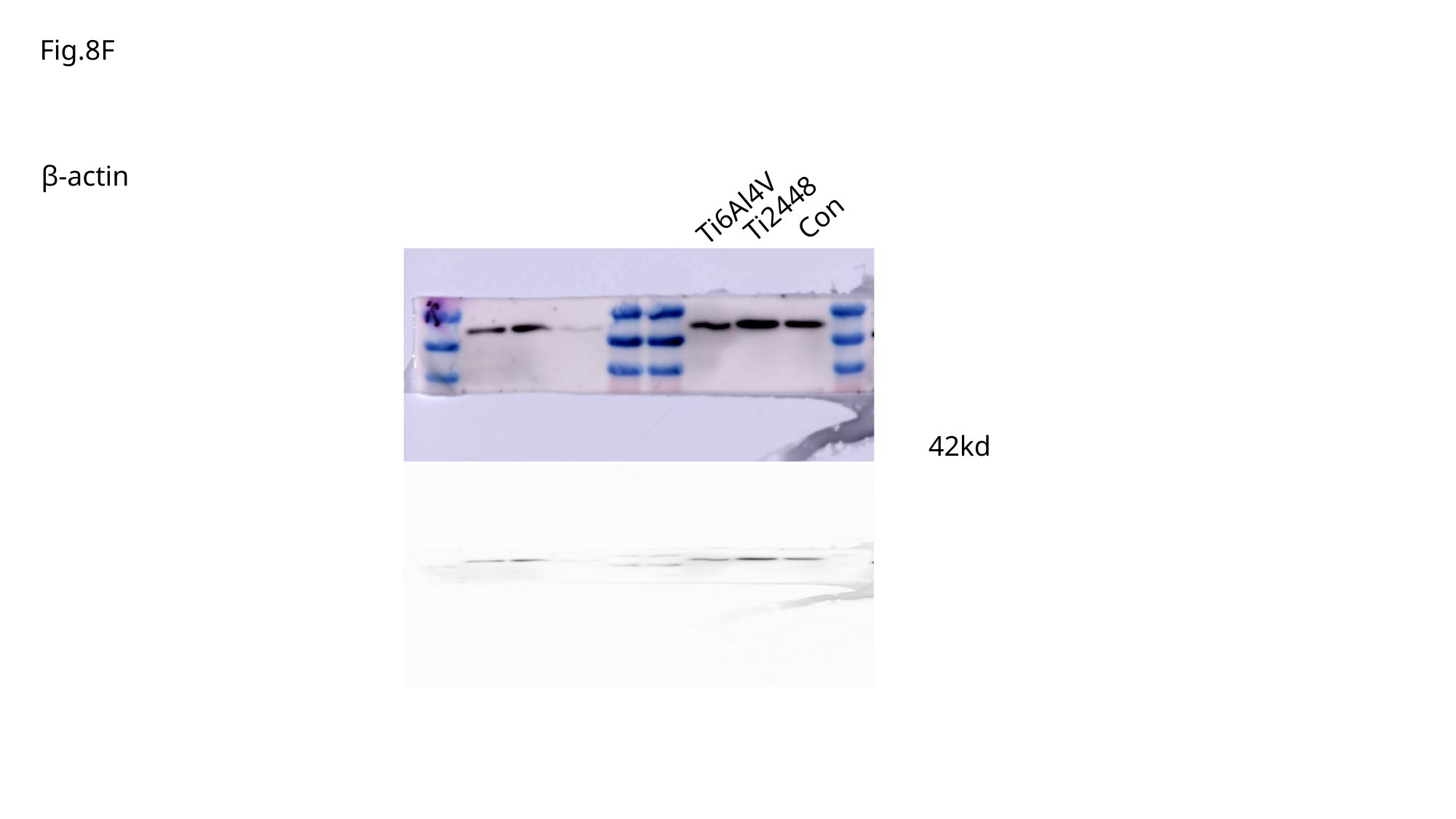

Fig.8F
β-actin
Ti6Al4V
Ti2448
Con
42kd

## Slide 4
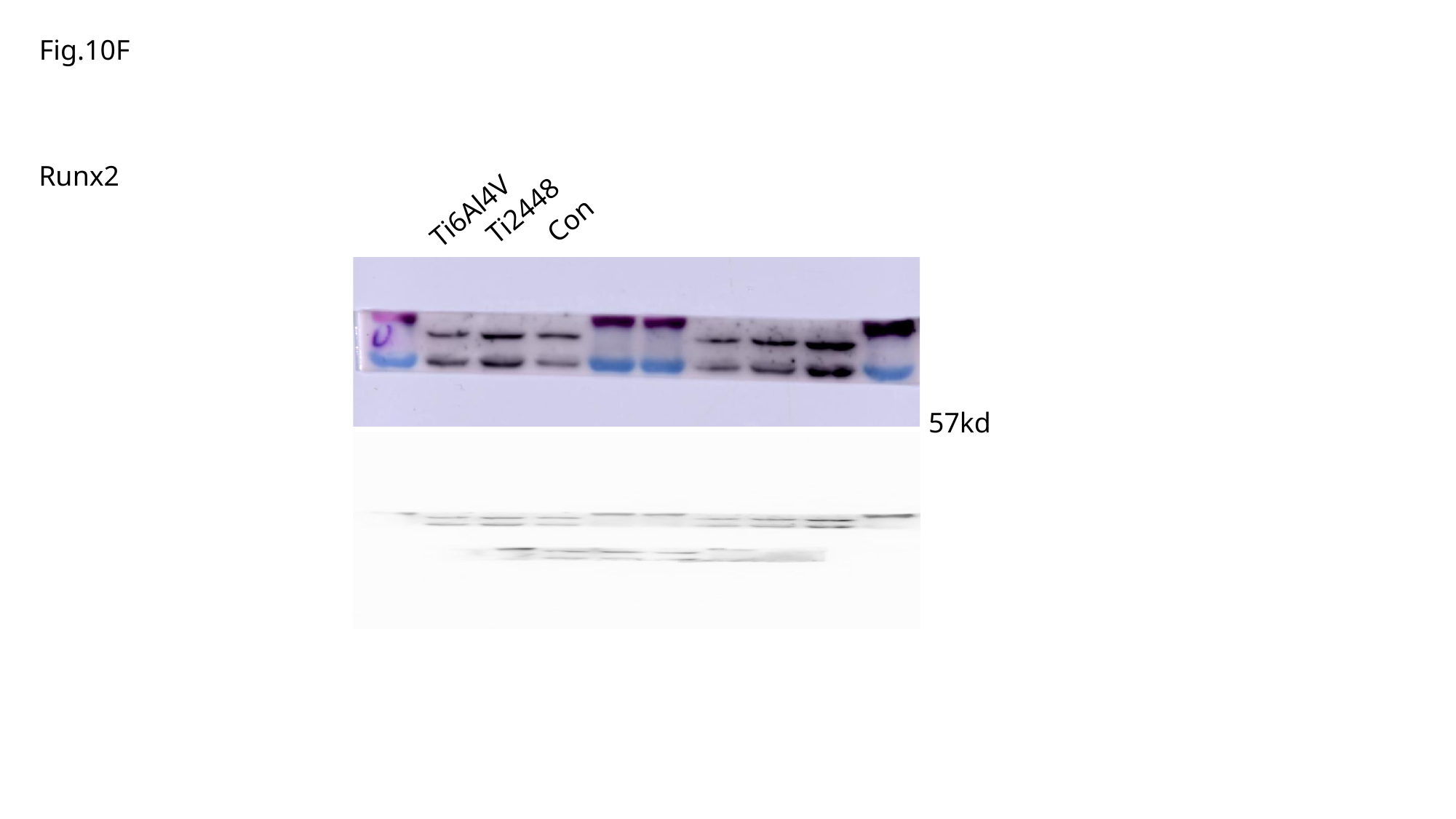

Fig.10F
Runx2
Ti6Al4V
Ti2448
Con
57kd

## Slide 5
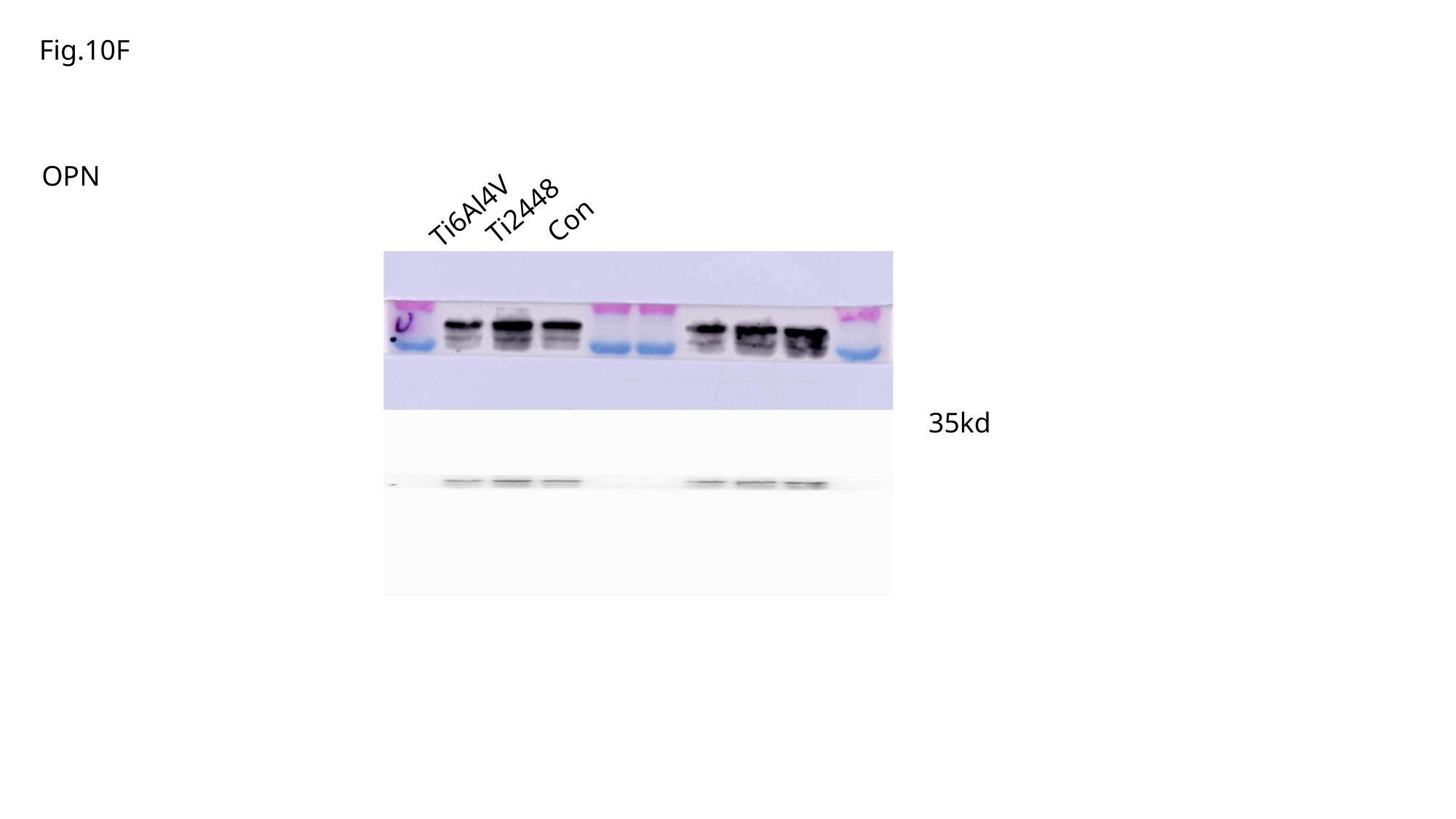

Fig.10F
OPN
Ti6Al4V
Ti2448
Con
35kd

## Slide 6
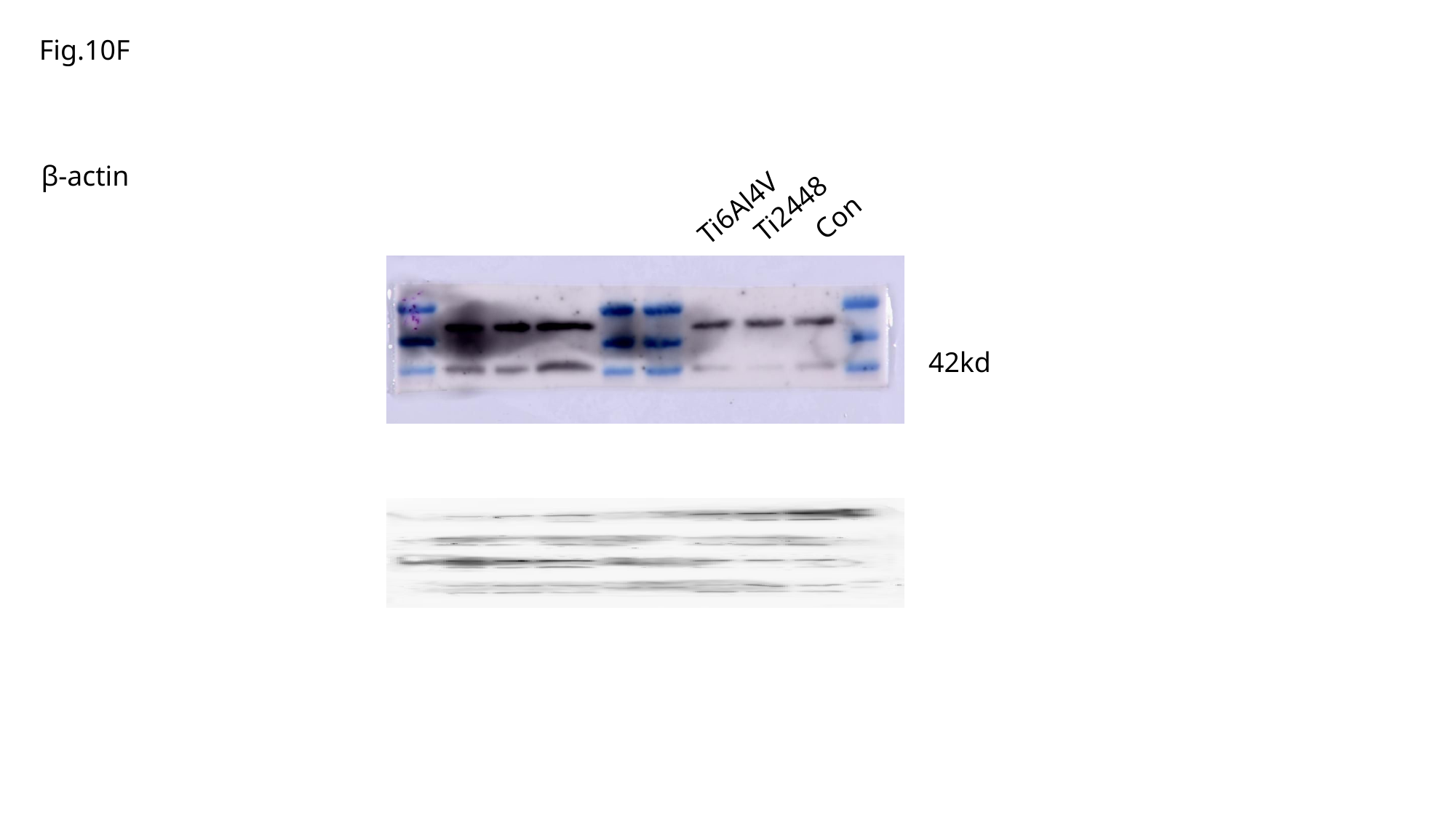

Fig.10F
β-actin
Ti6Al4V
Ti2448
Con
42kd

## Slide 7
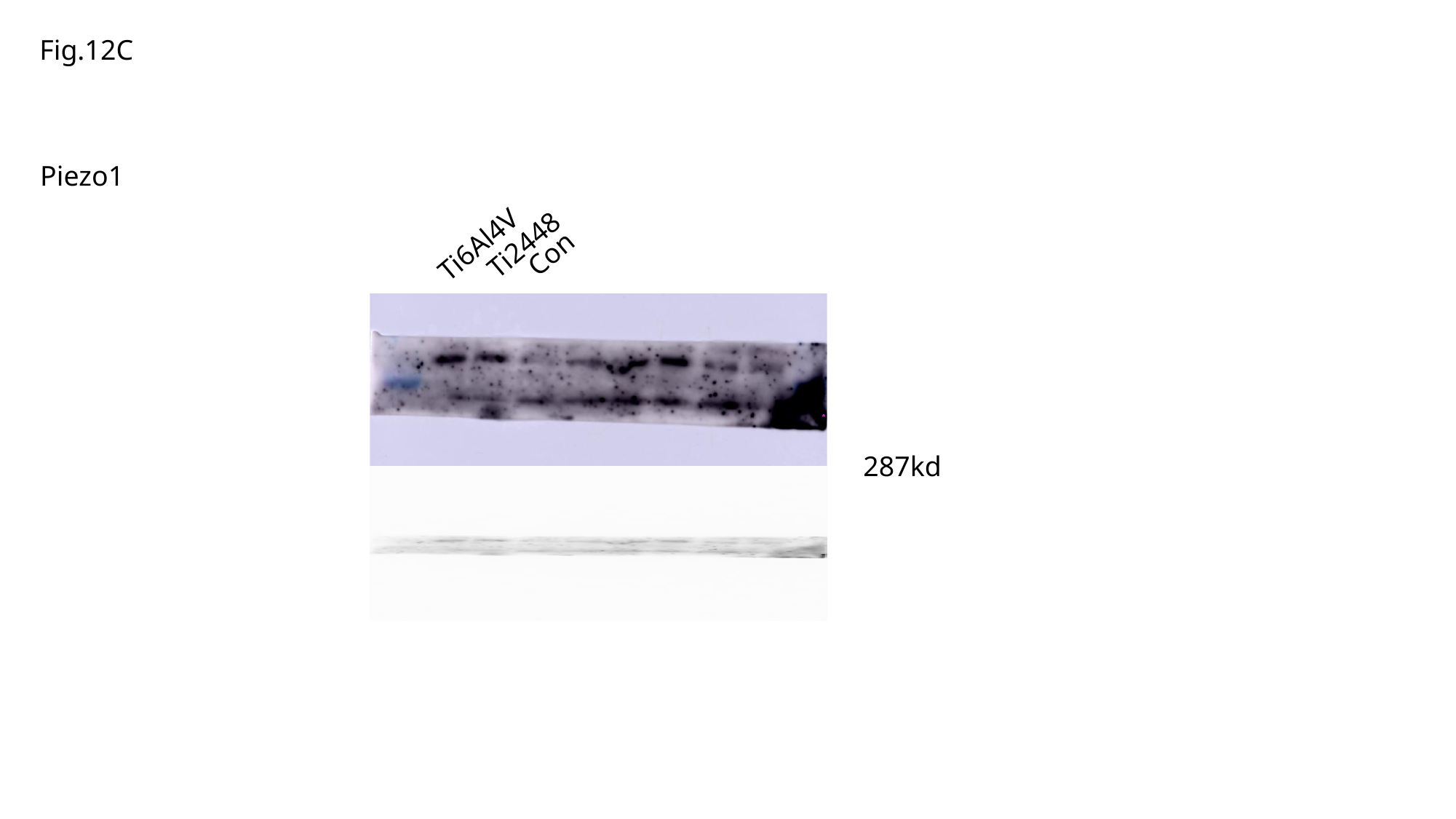

Fig.12C
Piezo1
Ti6Al4V
Ti2448
Con
287kd

## Slide 8
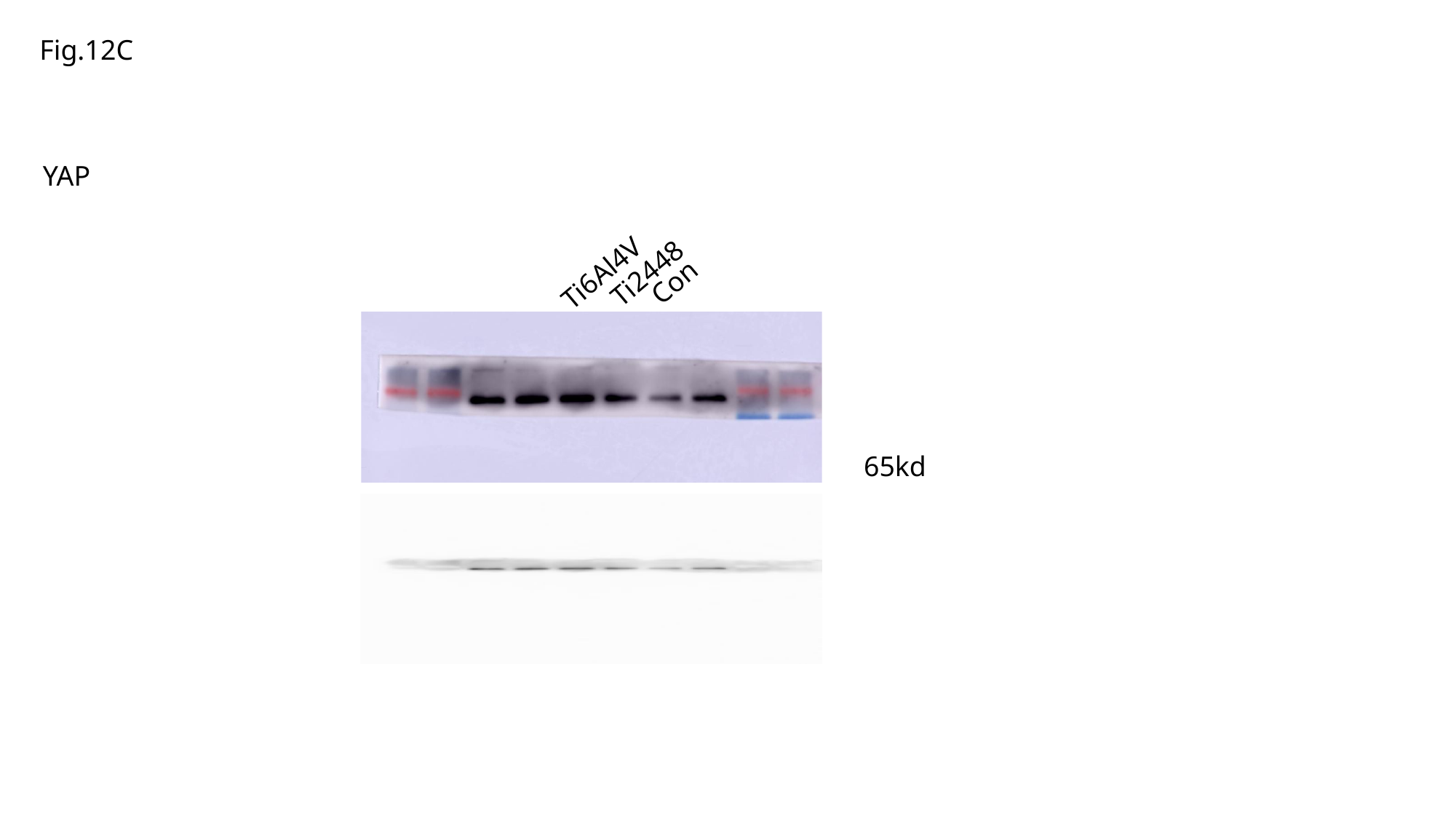

Fig.12C
YAP
Ti6Al4V
Ti2448
Con
65kd

## Slide 9
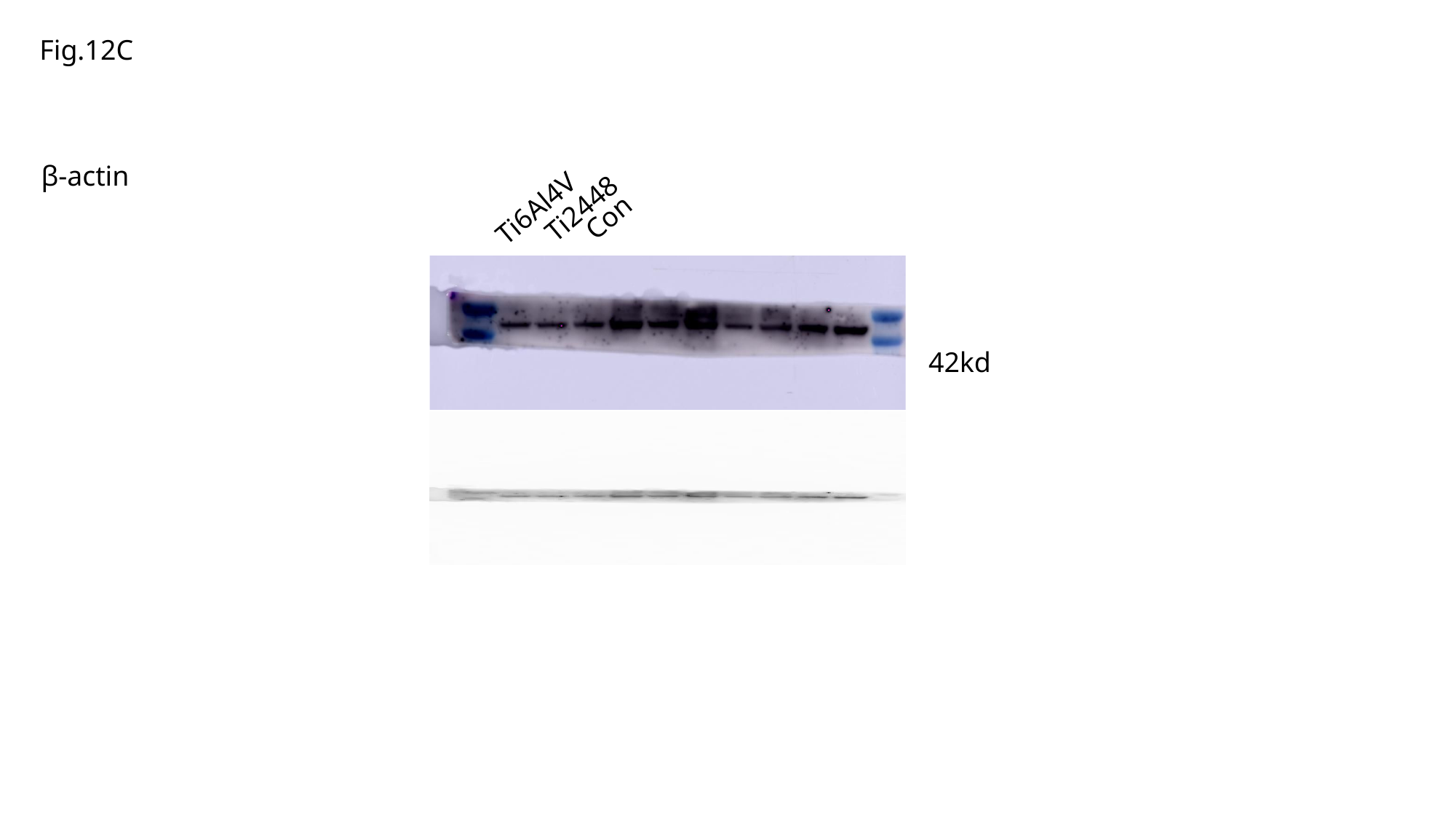

Fig.12C
β-actin
Ti6Al4V
Ti2448
Con
42kd

## Slide 10
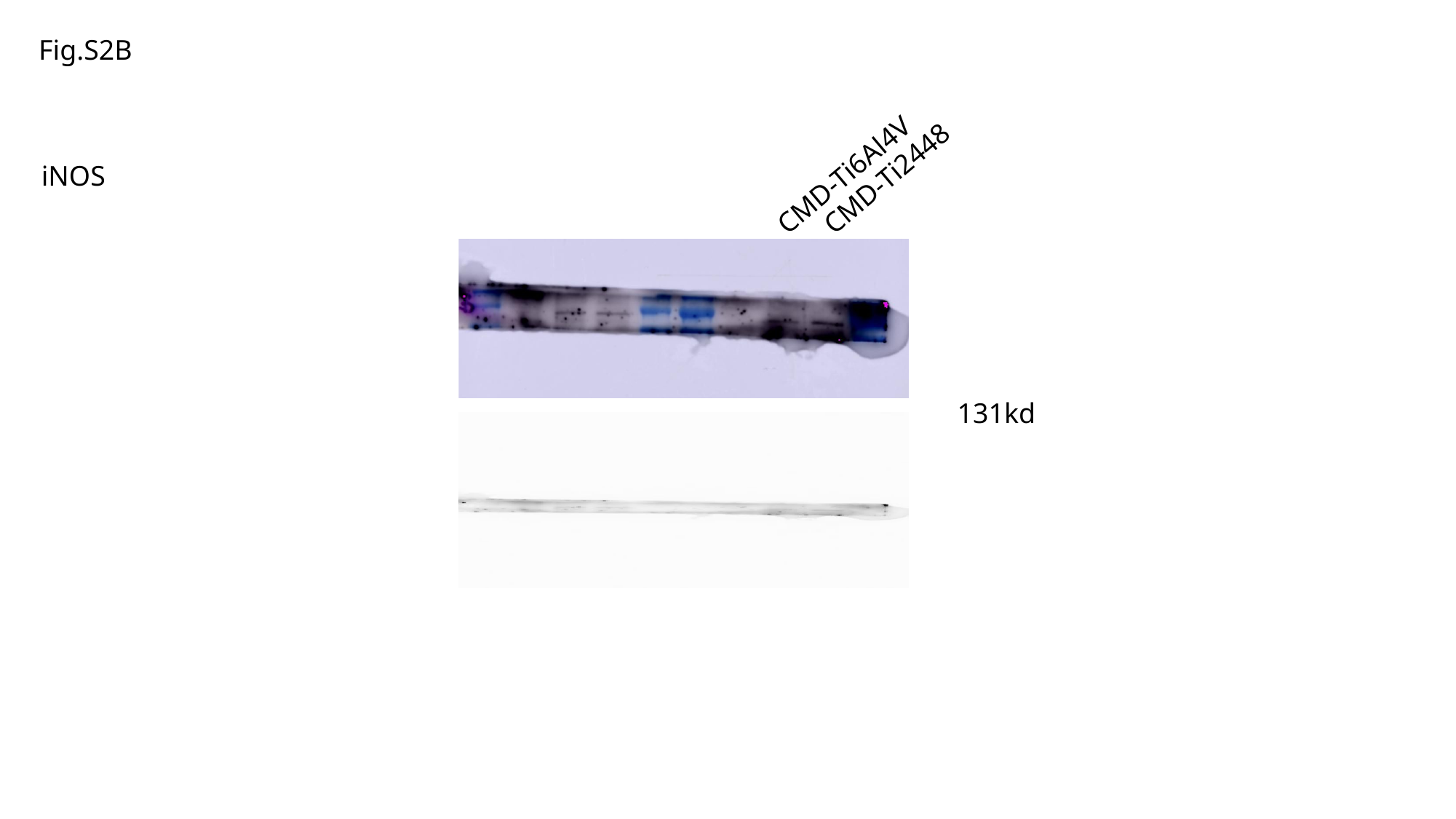

Fig.S2B
CMD-Ti6Al4V
iNOS
CMD-Ti2448
131kd

## Slide 11
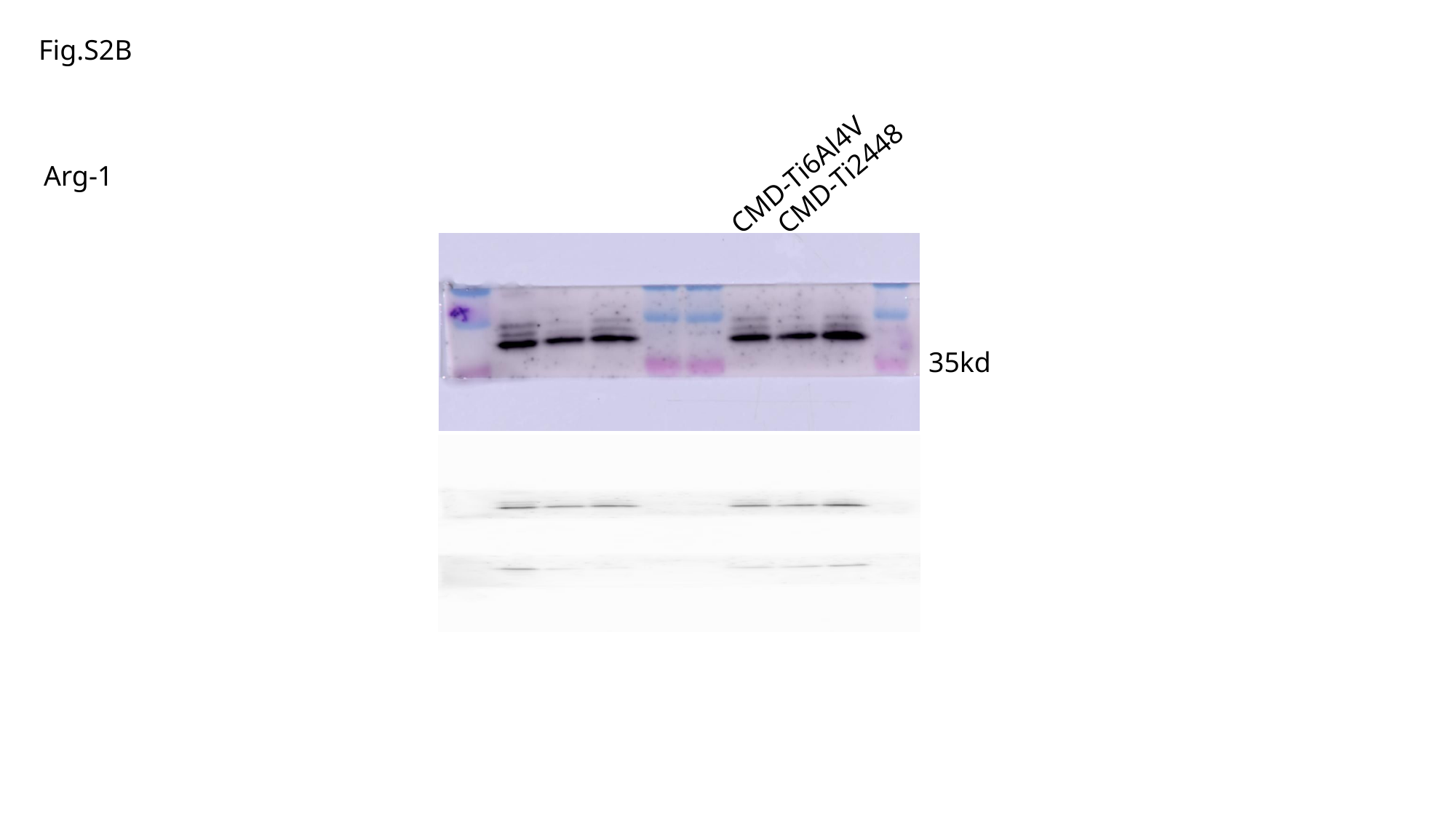

Fig.S2B
CMD-Ti6Al4V
Arg-1
CMD-Ti2448
35kd

## Slide 12
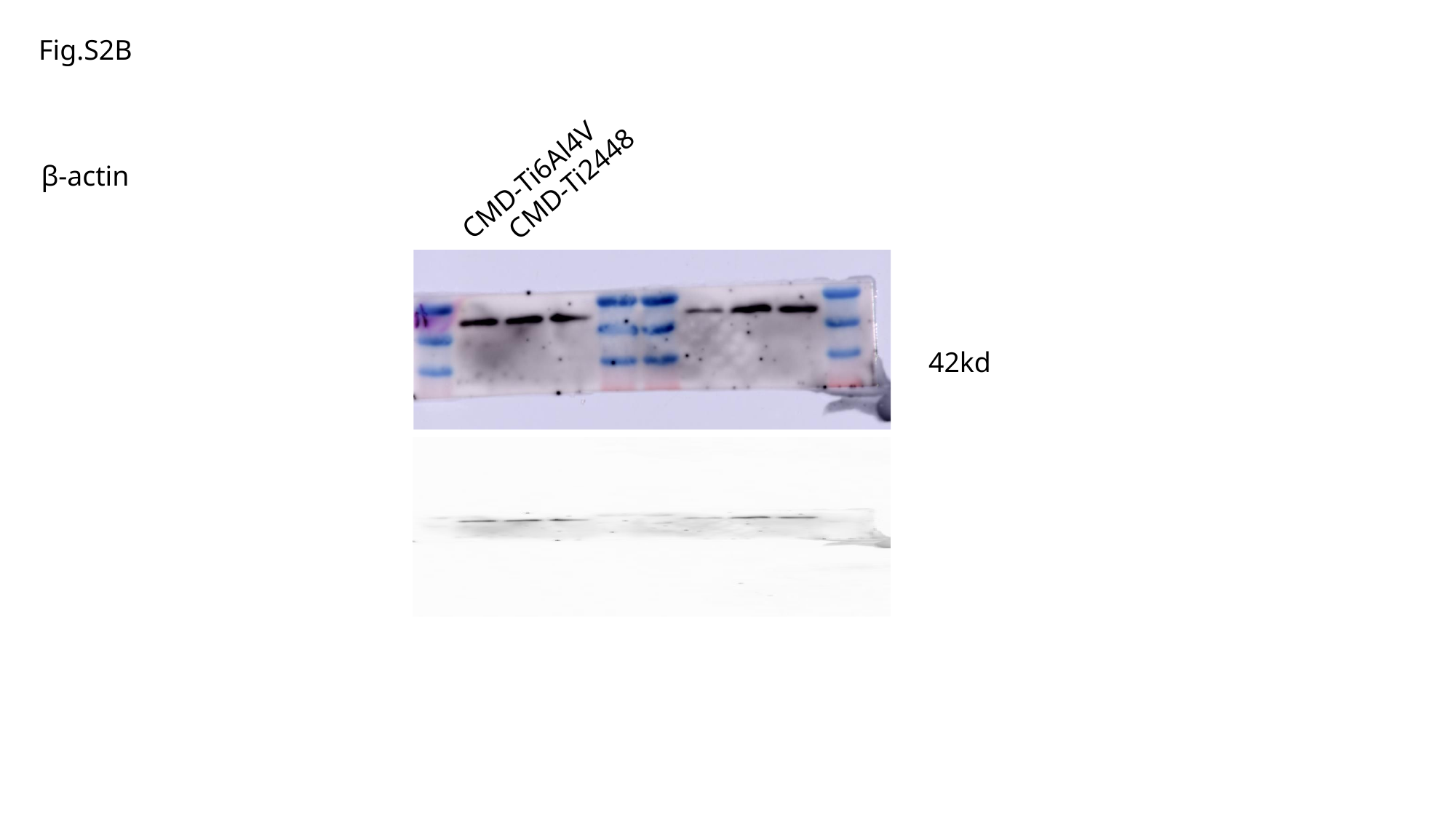

Fig.S2B
β-actin
CMD-Ti6Al4V
CMD-Ti2448
42kd
