# Supplementary material for: Three-Dimensionally Printed Ti2448 With Low Stiffness Enhanced Angiogenesis and Osteogenesis by Regulating Macrophage Polarization via Piezo1/YAP Signaling Axis
Source: Front Cell Dev Biol. 2021 Nov 15;9:750948. doi: 10.3389/fcell.2021.750948 (PMC8634253; doi:10.3389/fcell.2021.750948)
Supplement: Supplementary file 4 [file DataSheet9.zip › Raw data of osteogenesis in vivo/Raw data of osteogenesis in vivo .pptx]

## Slide 1
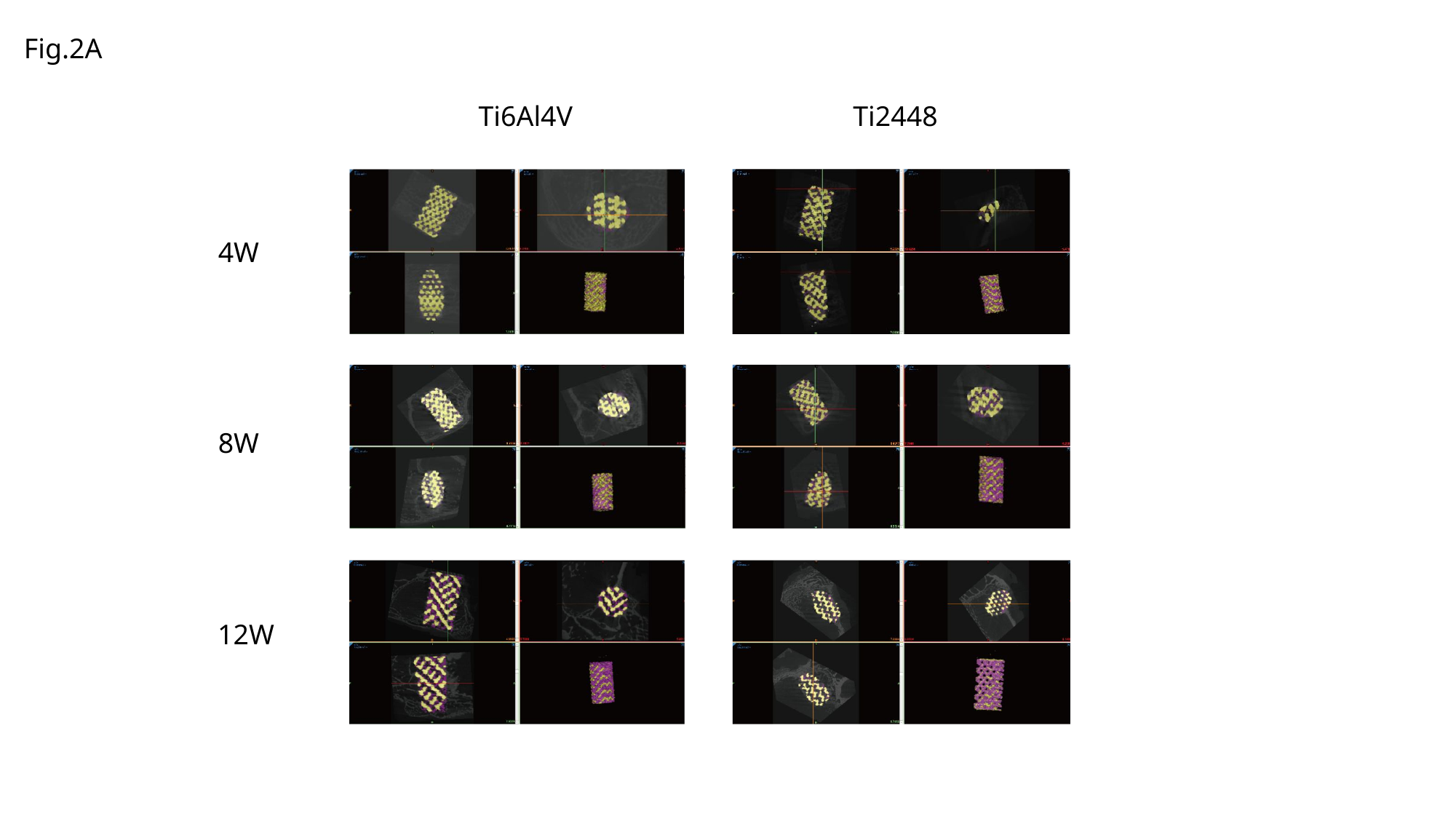

Fig.2A
Ti6Al4V
Ti2448
4W
8W
12W

## Slide 2
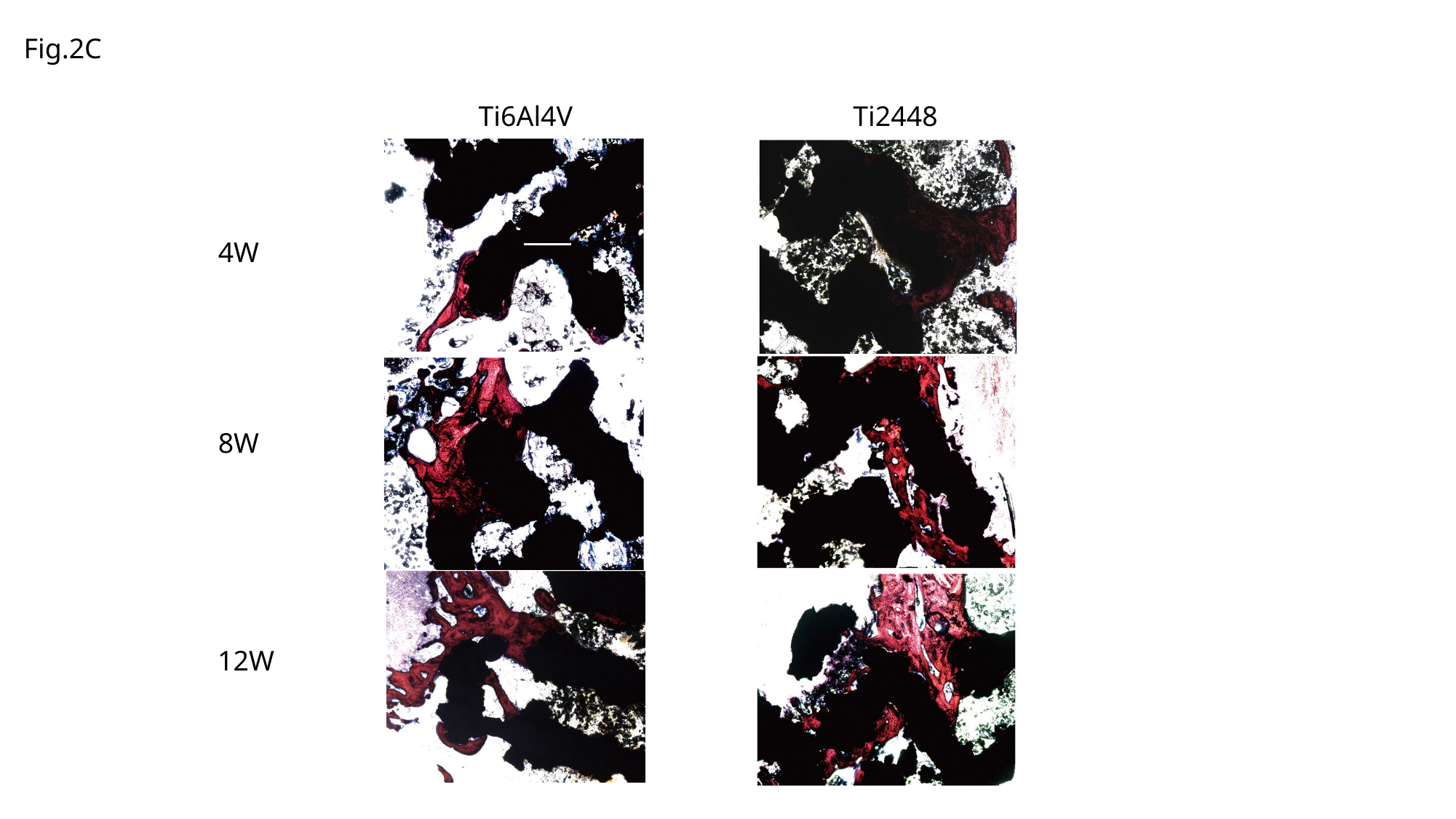

Fig.2C
Ti6Al4V
Ti2448
4W
8W
12W

## Slide 3
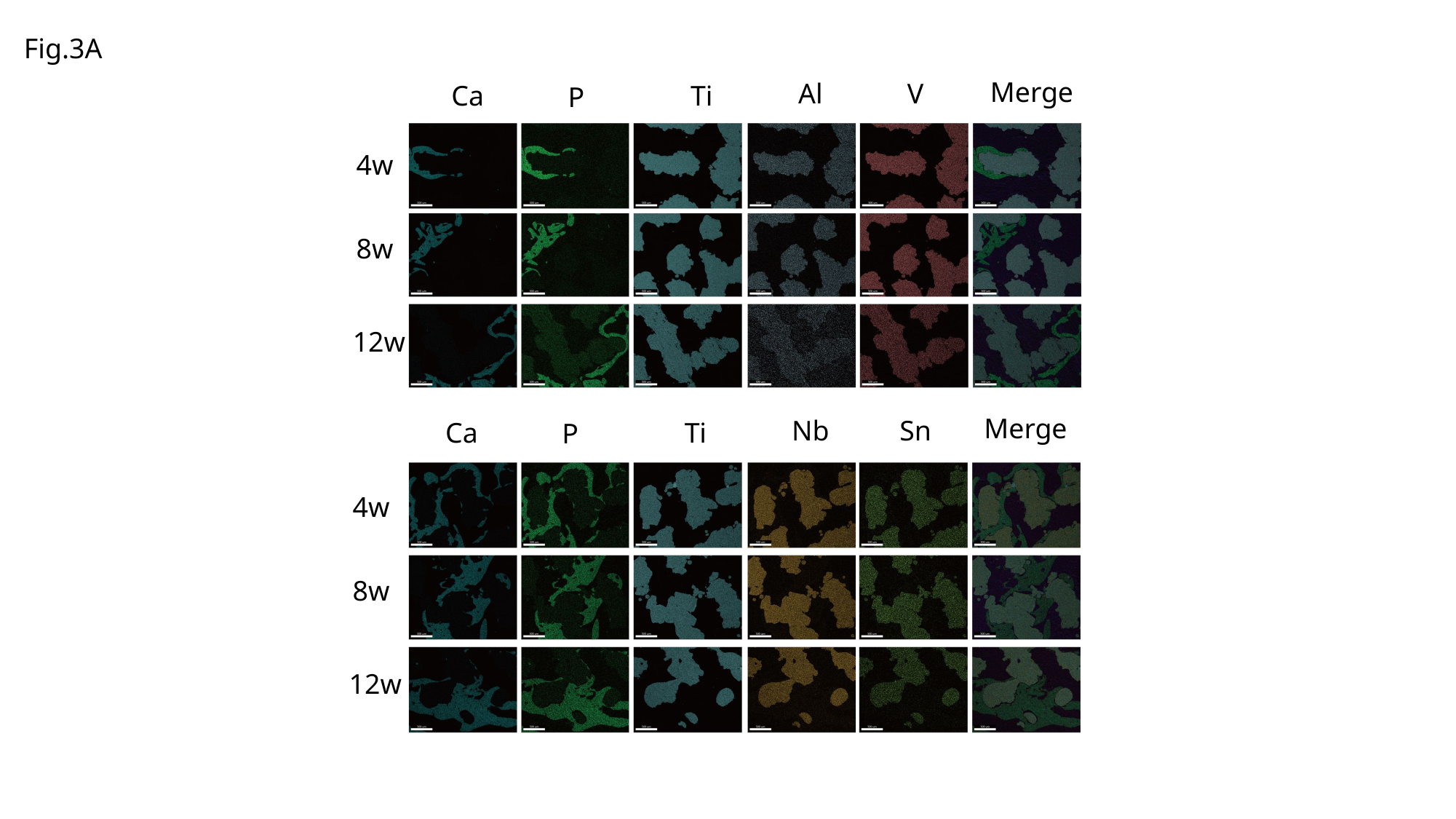

Fig.3A
Merge
Al
V
Ca
Ti
P
4w
8w
12w
Merge
Nb
Sn
Ca
Ti
P
4w
8w
12w
