# Supplementary material for: Three-Dimensionally Printed Ti2448 With Low Stiffness Enhanced Angiogenesis and Osteogenesis by Regulating Macrophage Polarization via Piezo1/YAP Signaling Axis
Source: Front Cell Dev Biol. 2021 Nov 15;9:750948. doi: 10.3389/fcell.2021.750948 (PMC8634253; doi:10.3389/fcell.2021.750948)
Supplement: Supplementary file 5 [file DataSheet4.zip › Raw data of Elisa/Raw data of Elisa.pptx]

## Slide 1
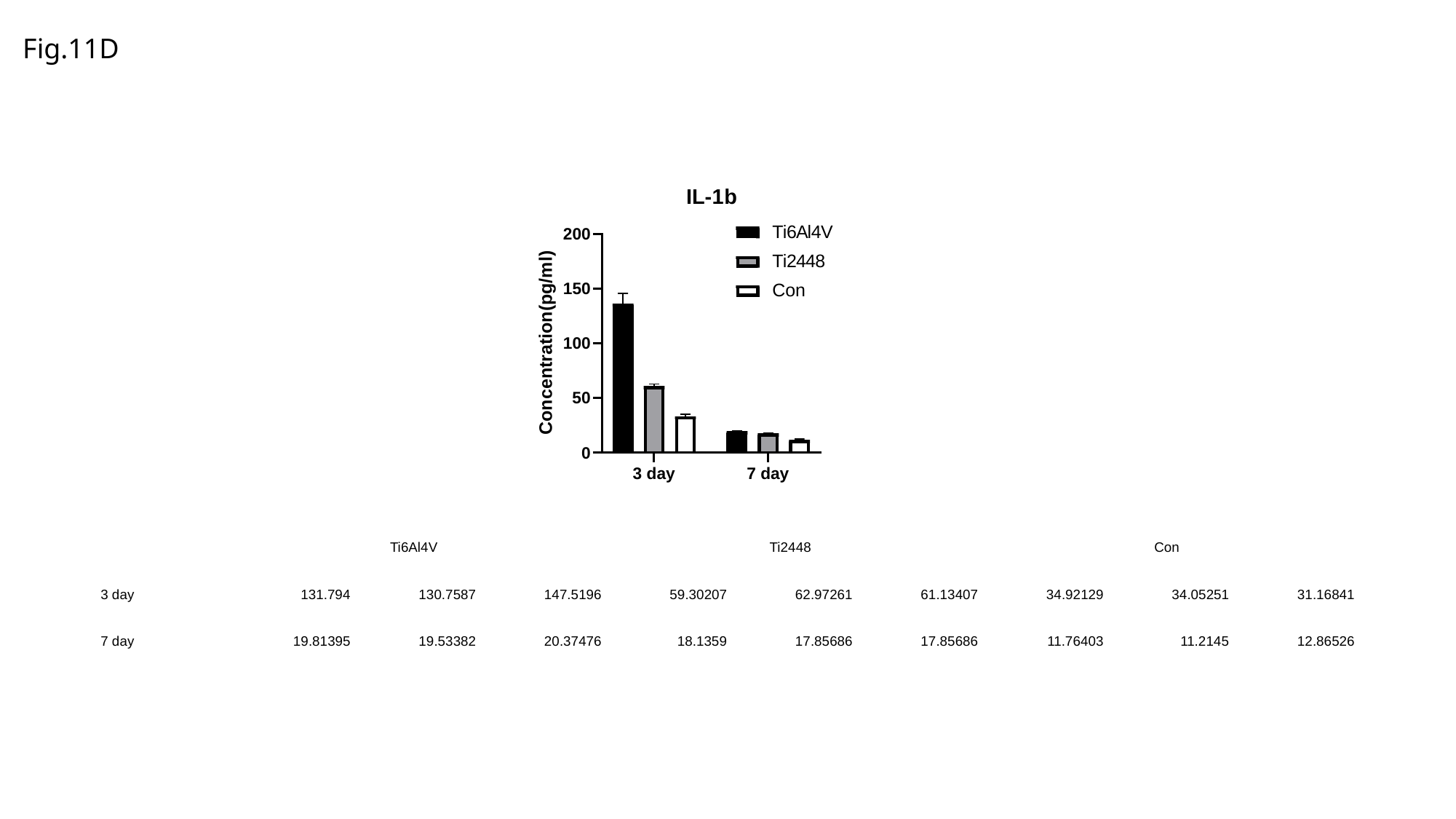

Fig.11D
| | Ti6Al4V | | | Ti2448 | | | Con | | |
| --- | --- | --- | --- | --- | --- | --- | --- | --- | --- |
| 3 day | 131.794 | 130.7587 | 147.5196 | 59.30207 | 62.97261 | 61.13407 | 34.92129 | 34.05251 | 31.16841 |
| 7 day | 19.81395 | 19.53382 | 20.37476 | 18.1359 | 17.85686 | 17.85686 | 11.76403 | 11.2145 | 12.86526 |

## Slide 2
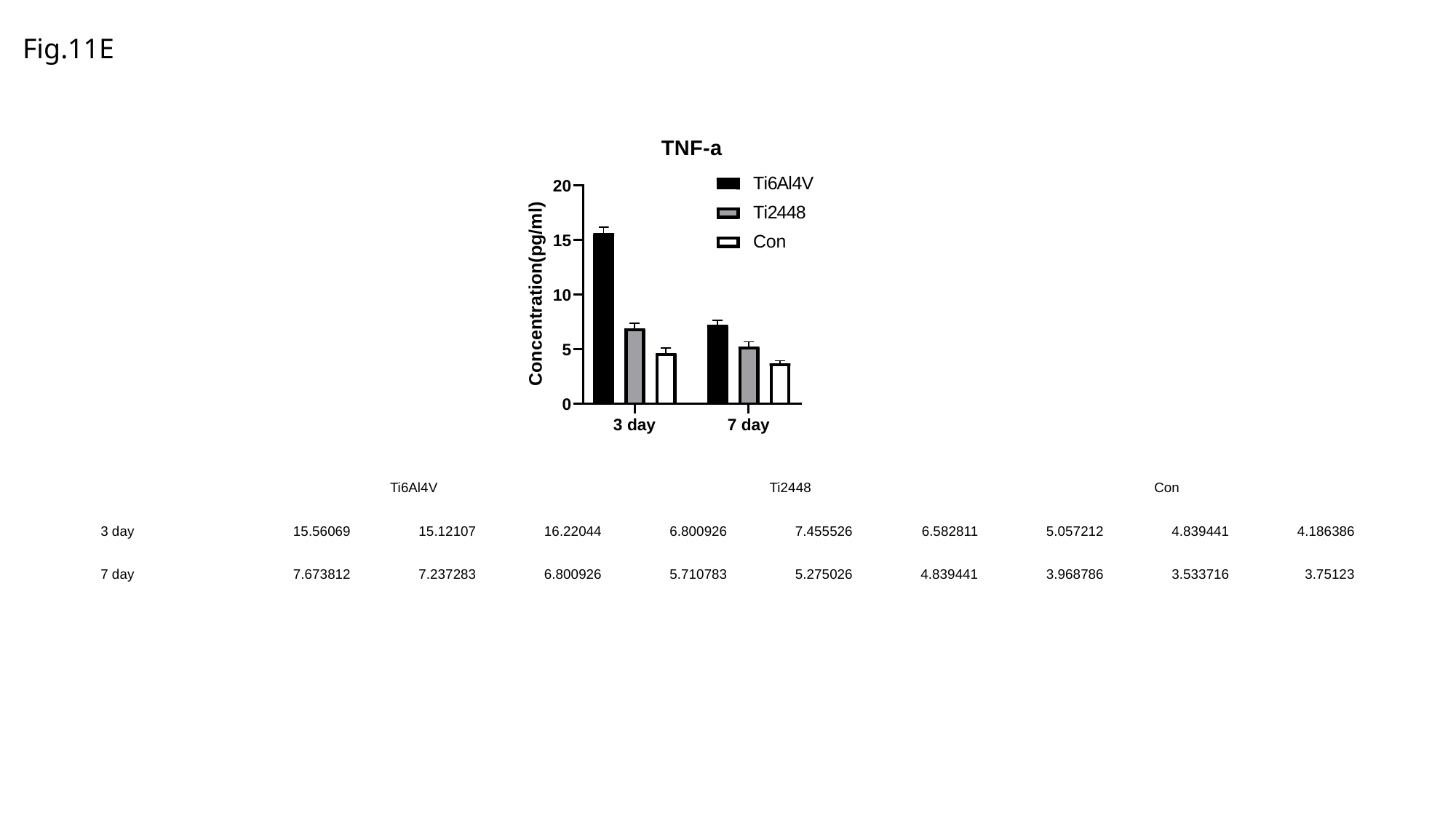

Fig.11E
| | Ti6Al4V | | | Ti2448 | | | Con | | |
| --- | --- | --- | --- | --- | --- | --- | --- | --- | --- |
| 3 day | 15.56069 | 15.12107 | 16.22044 | 6.800926 | 7.455526 | 6.582811 | 5.057212 | 4.839441 | 4.186386 |
| 7 day | 7.673812 | 7.237283 | 6.800926 | 5.710783 | 5.275026 | 4.839441 | 3.968786 | 3.533716 | 3.75123 |

## Slide 3
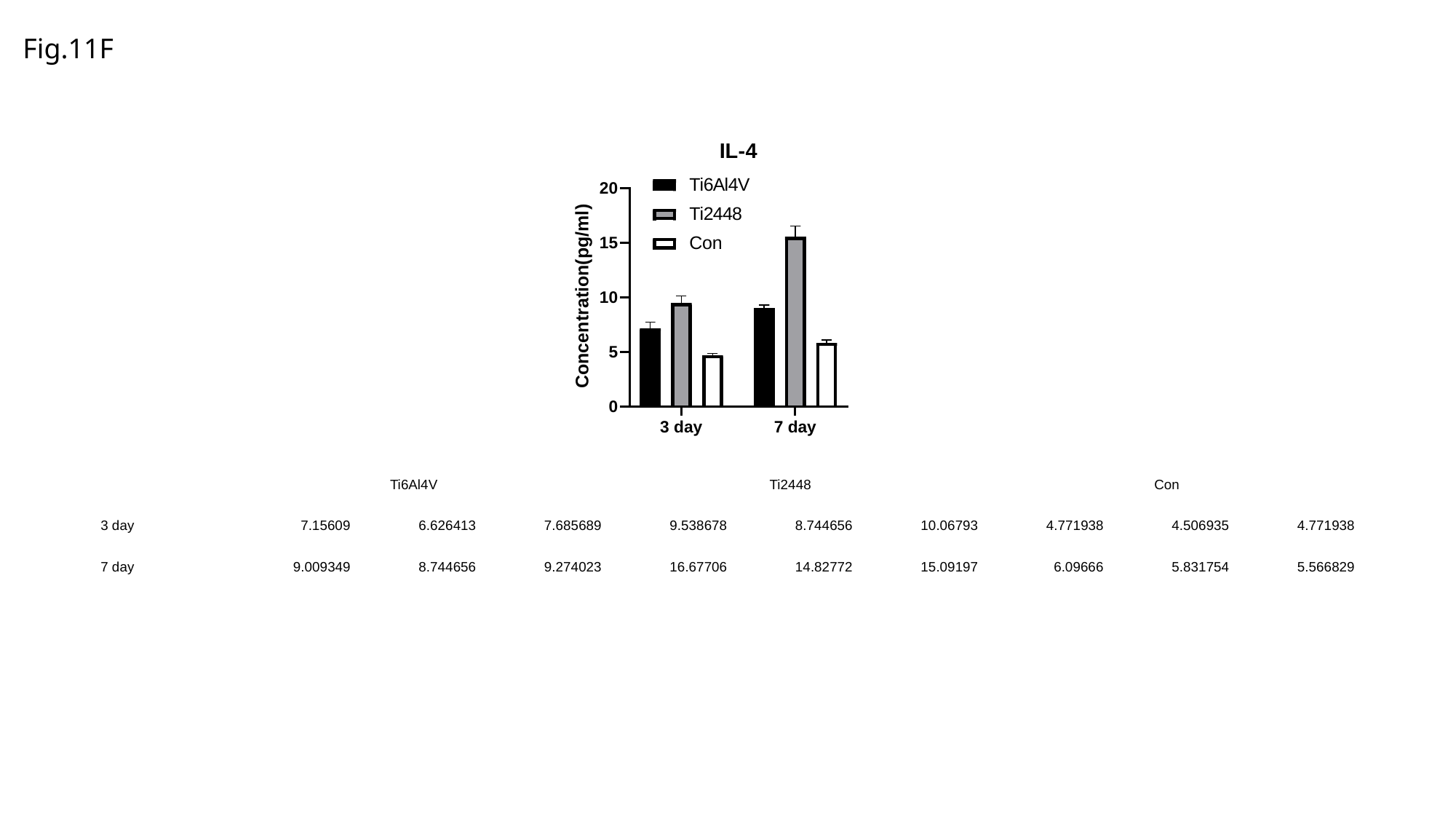

Fig.11F
| | Ti6Al4V | | | Ti2448 | | | Con | | |
| --- | --- | --- | --- | --- | --- | --- | --- | --- | --- |
| 3 day | 7.15609 | 6.626413 | 7.685689 | 9.538678 | 8.744656 | 10.06793 | 4.771938 | 4.506935 | 4.771938 |
| 7 day | 9.009349 | 8.744656 | 9.274023 | 16.67706 | 14.82772 | 15.09197 | 6.09666 | 5.831754 | 5.566829 |

## Slide 4
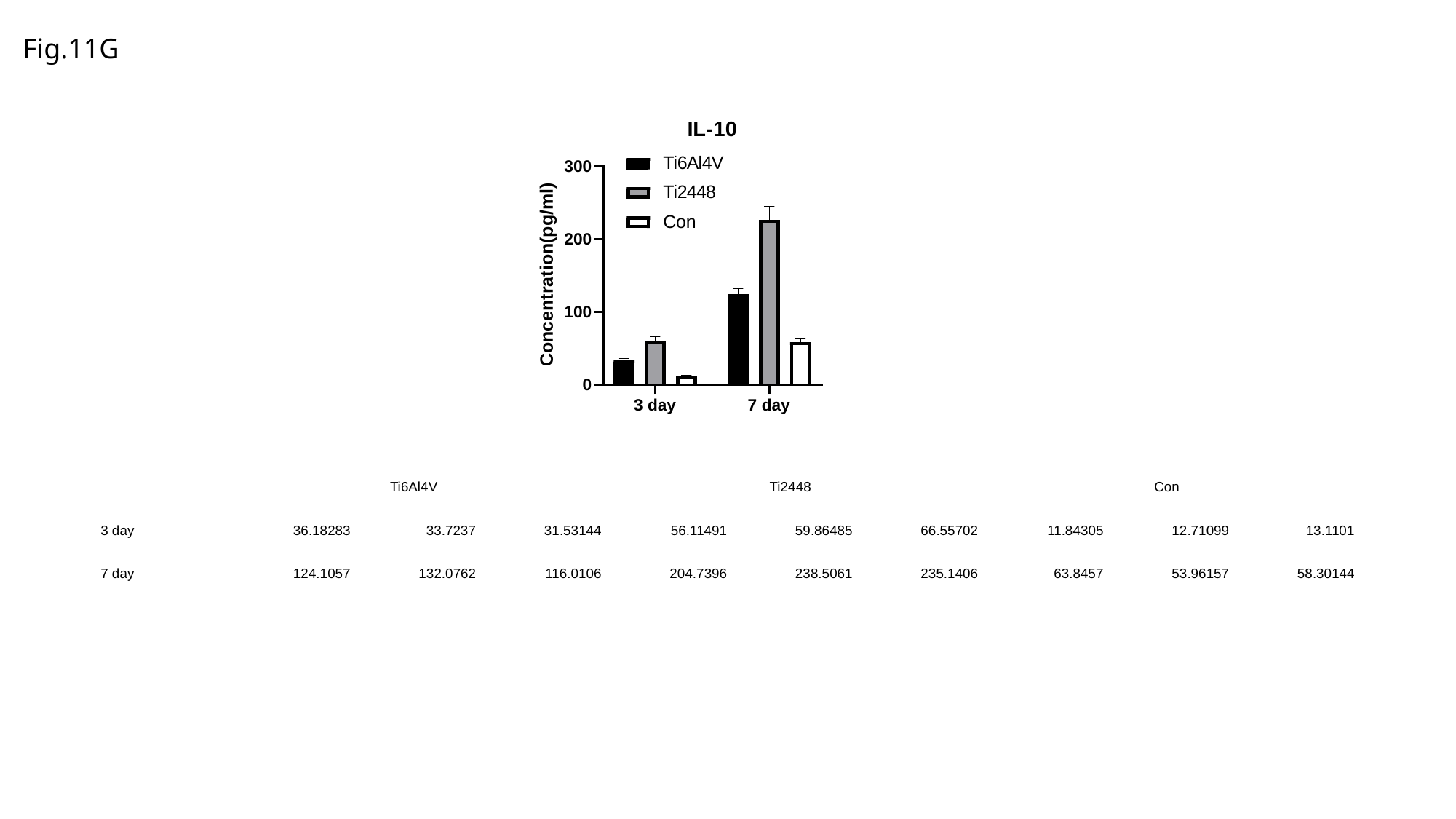

Fig.11G
| | Ti6Al4V | | | Ti2448 | | | Con | | |
| --- | --- | --- | --- | --- | --- | --- | --- | --- | --- |
| 3 day | 36.18283 | 33.7237 | 31.53144 | 56.11491 | 59.86485 | 66.55702 | 11.84305 | 12.71099 | 13.1101 |
| 7 day | 124.1057 | 132.0762 | 116.0106 | 204.7396 | 238.5061 | 235.1406 | 63.8457 | 53.96157 | 58.30144 |

## Slide 5
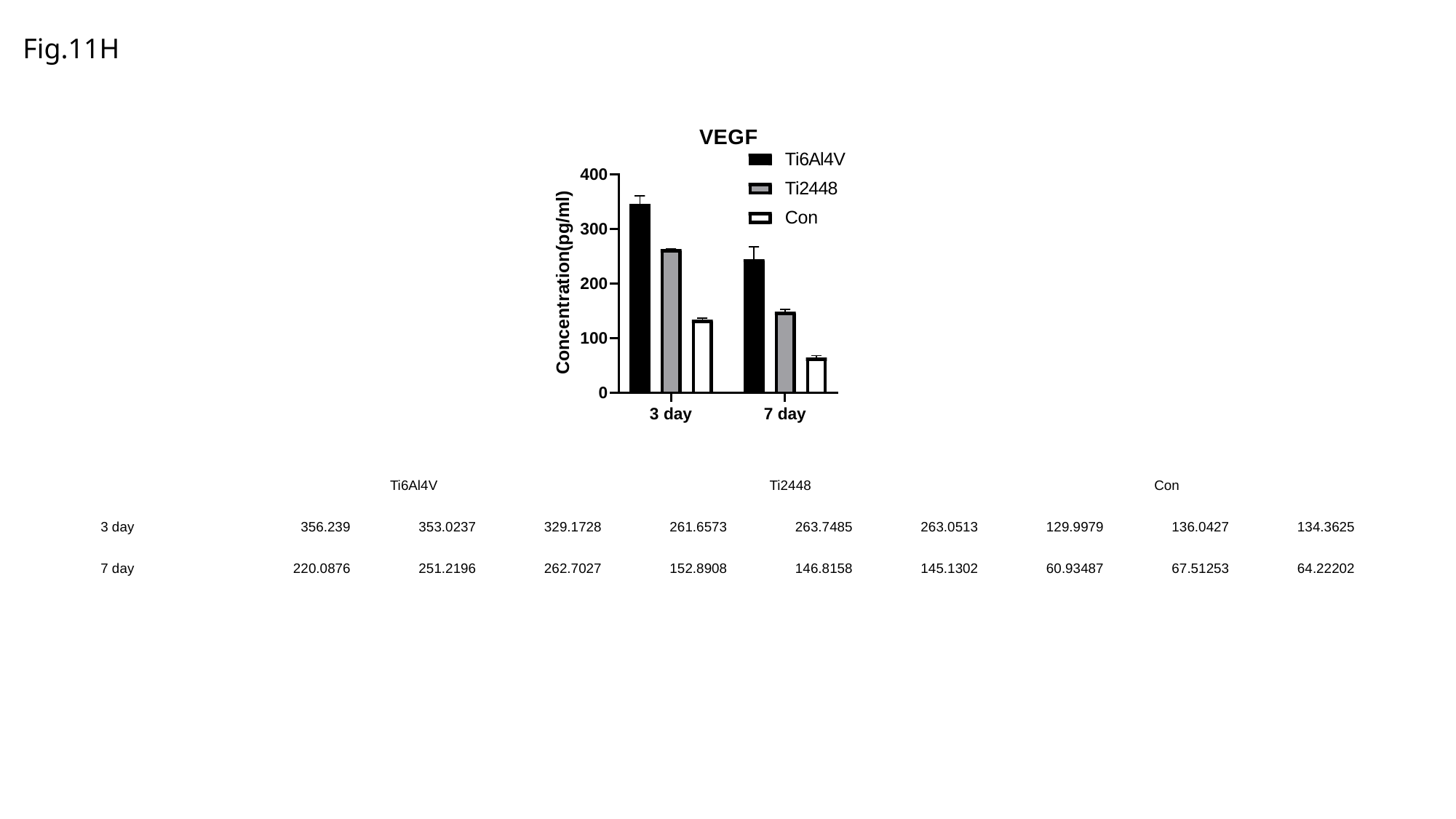

Fig.11H
| | Ti6Al4V | | | Ti2448 | | | Con | | |
| --- | --- | --- | --- | --- | --- | --- | --- | --- | --- |
| 3 day | 356.239 | 353.0237 | 329.1728 | 261.6573 | 263.7485 | 263.0513 | 129.9979 | 136.0427 | 134.3625 |
| 7 day | 220.0876 | 251.2196 | 262.7027 | 152.8908 | 146.8158 | 145.1302 | 60.93487 | 67.51253 | 64.22202 |

## Slide 6
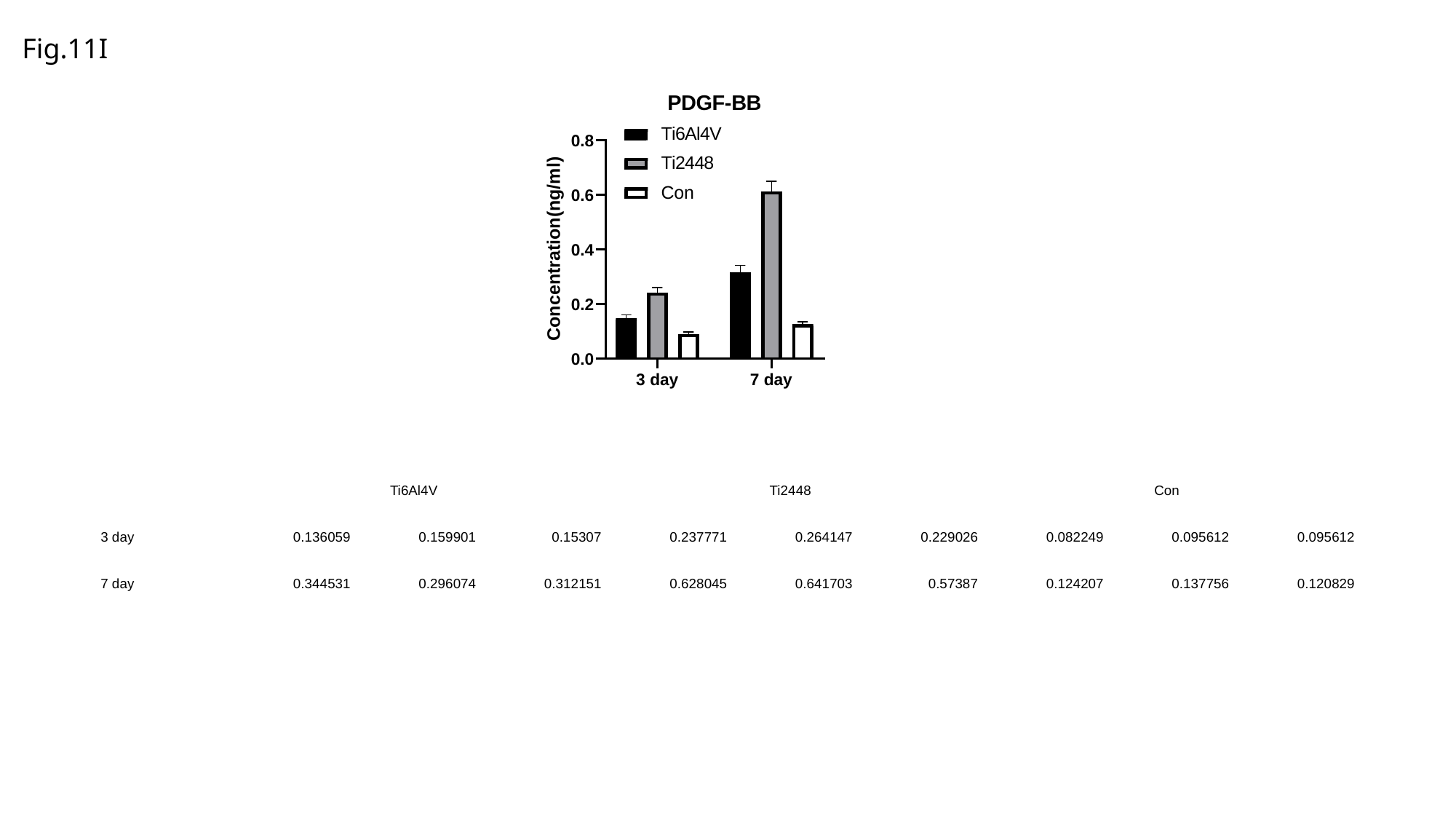

Fig.11I
| | Ti6Al4V | | | Ti2448 | | | Con | | |
| --- | --- | --- | --- | --- | --- | --- | --- | --- | --- |
| 3 day | 0.136059 | 0.159901 | 0.15307 | 0.237771 | 0.264147 | 0.229026 | 0.082249 | 0.095612 | 0.095612 |
| 7 day | 0.344531 | 0.296074 | 0.312151 | 0.628045 | 0.641703 | 0.57387 | 0.124207 | 0.137756 | 0.120829 |

## Slide 7
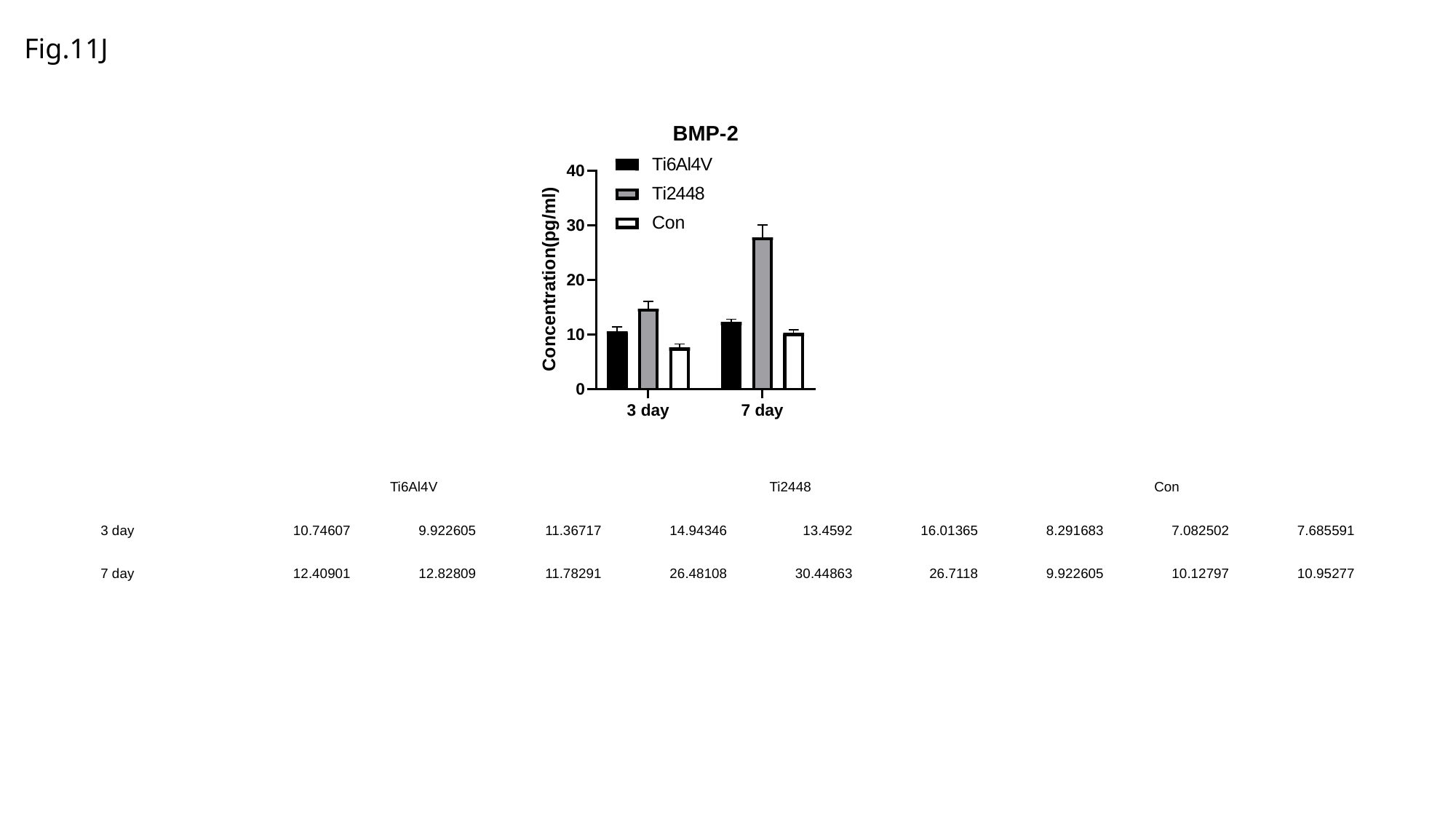

Fig.11J
| | Ti6Al4V | | | Ti2448 | | | Con | | |
| --- | --- | --- | --- | --- | --- | --- | --- | --- | --- |
| 3 day | 10.74607 | 9.922605 | 11.36717 | 14.94346 | 13.4592 | 16.01365 | 8.291683 | 7.082502 | 7.685591 |
| 7 day | 12.40901 | 12.82809 | 11.78291 | 26.48108 | 30.44863 | 26.7118 | 9.922605 | 10.12797 | 10.95277 |

## Slide 8
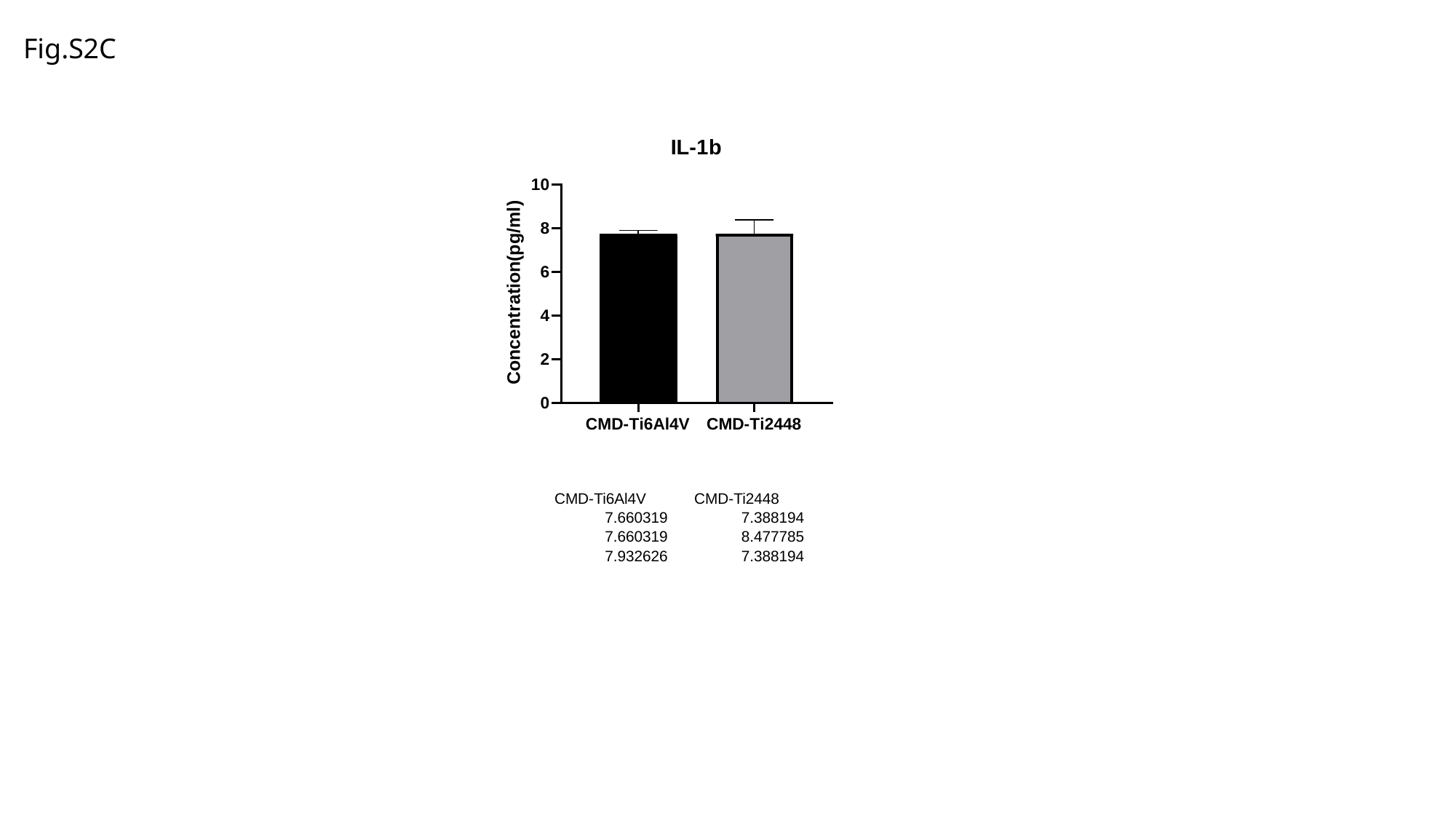

Fig.S2C
| CMD-Ti6Al4V | CMD-Ti2448 |
| --- | --- |
| 7.660319 | 7.388194 |
| 7.660319 | 8.477785 |
| 7.932626 | 7.388194 |

## Slide 9
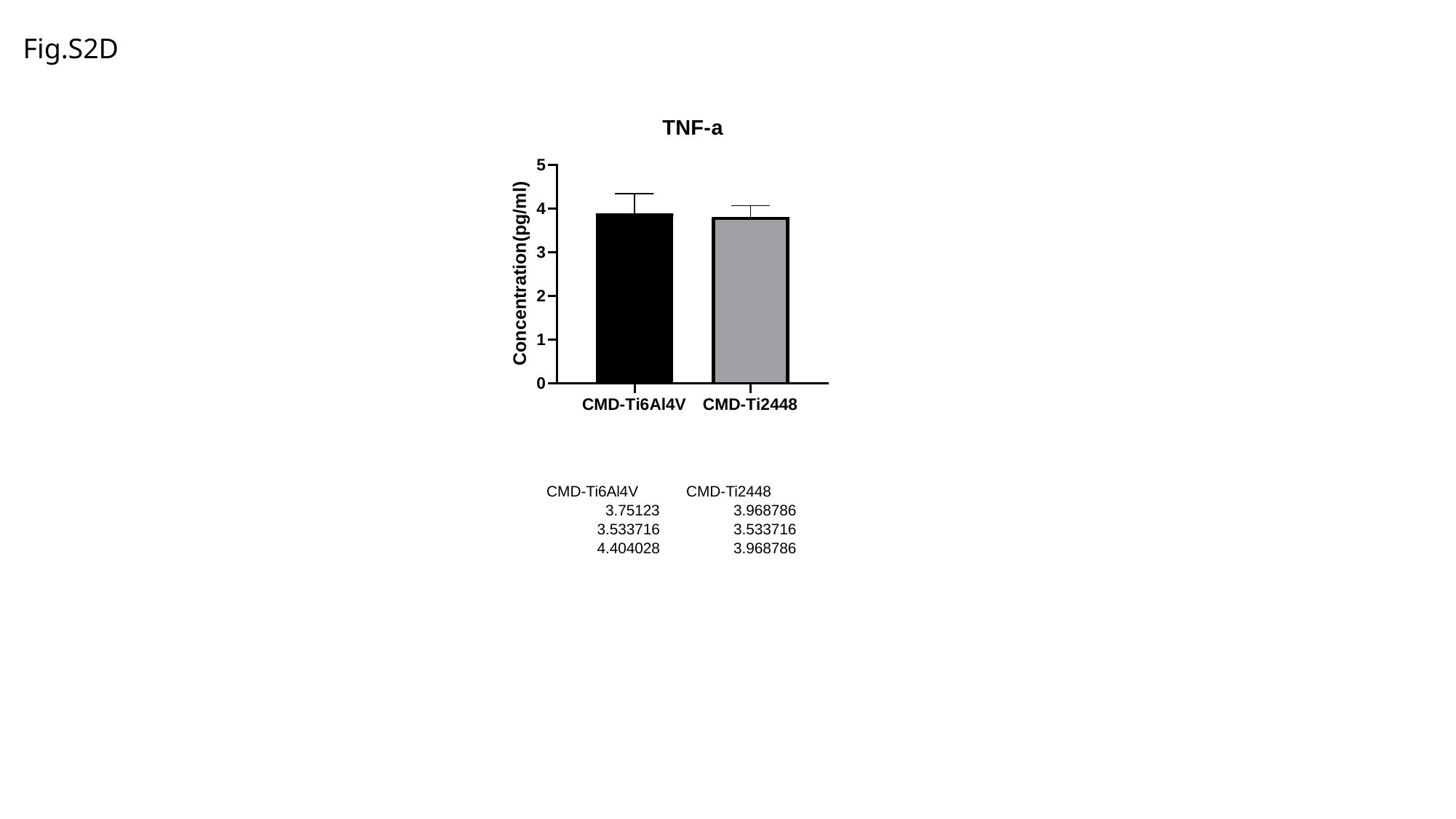

Fig.S2D
| CMD-Ti6Al4V | CMD-Ti2448 |
| --- | --- |
| 3.75123 | 3.968786 |
| 3.533716 | 3.533716 |
| 4.404028 | 3.968786 |

## Slide 10
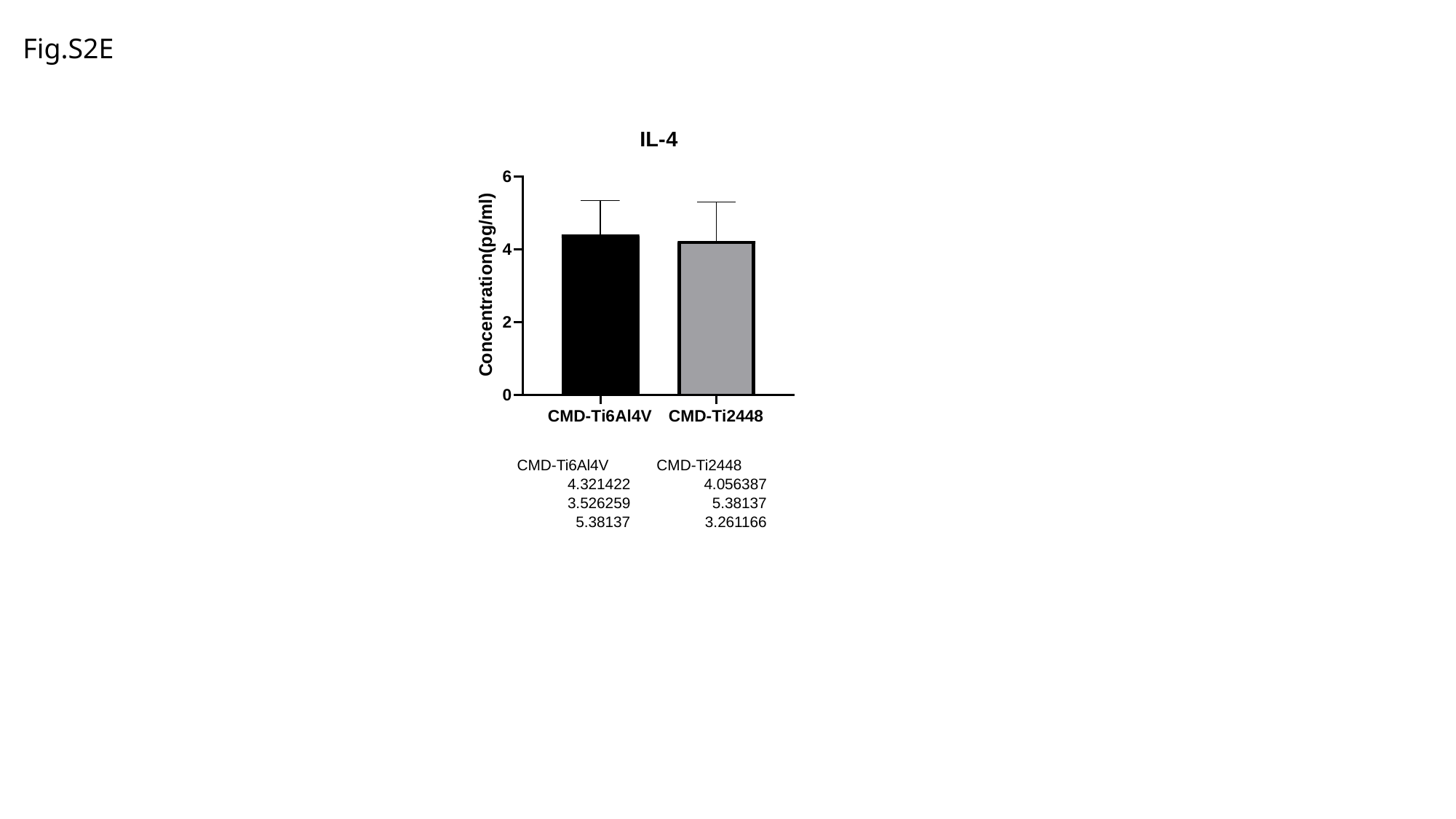

Fig.S2E
| CMD-Ti6Al4V | CMD-Ti2448 |
| --- | --- |
| 4.321422 | 4.056387 |
| 3.526259 | 5.38137 |
| 5.38137 | 3.261166 |

## Slide 11
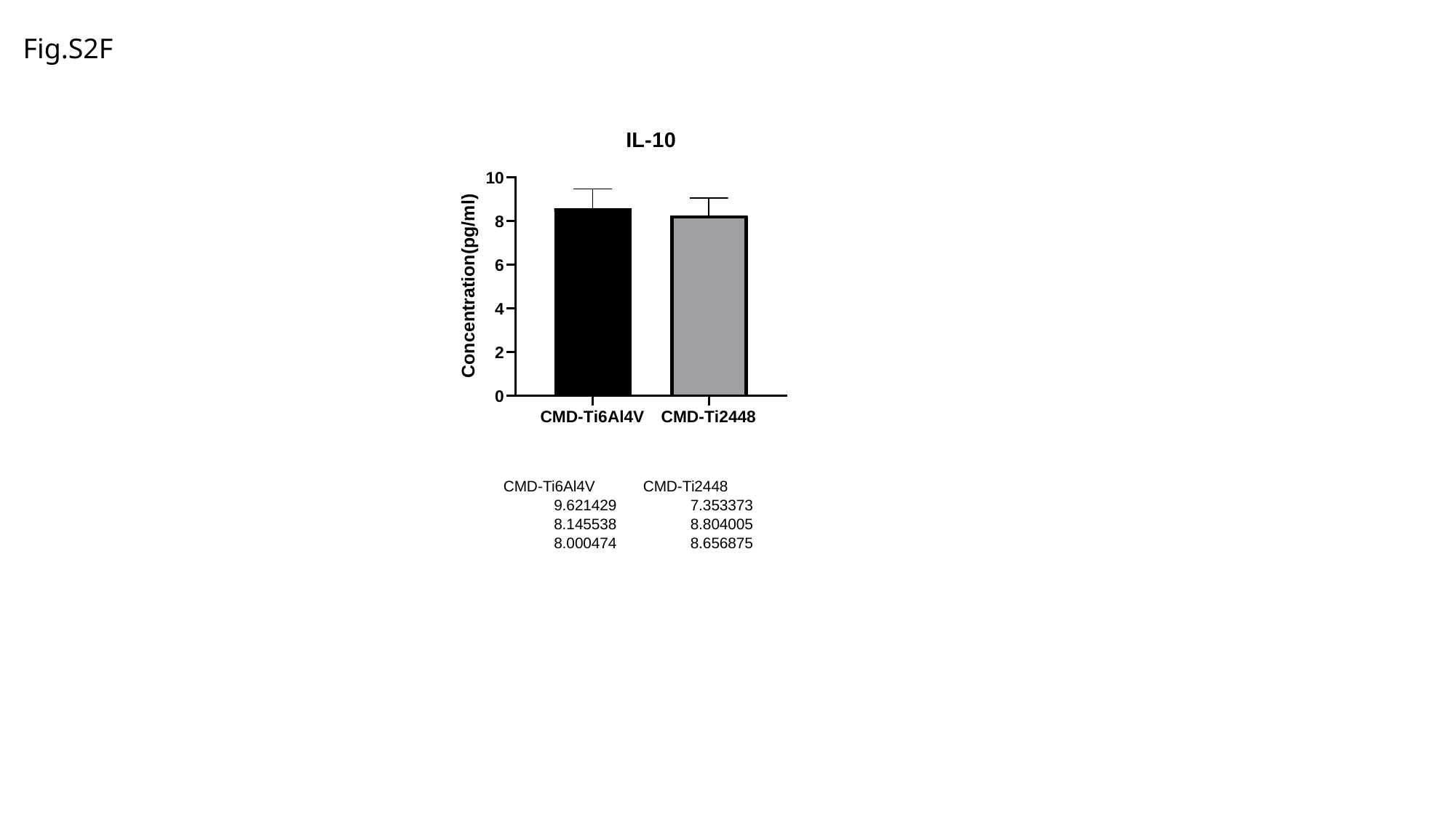

Fig.S2F
| CMD-Ti6Al4V | CMD-Ti2448 |
| --- | --- |
| 9.621429 | 7.353373 |
| 8.145538 | 8.804005 |
| 8.000474 | 8.656875 |

## Slide 12
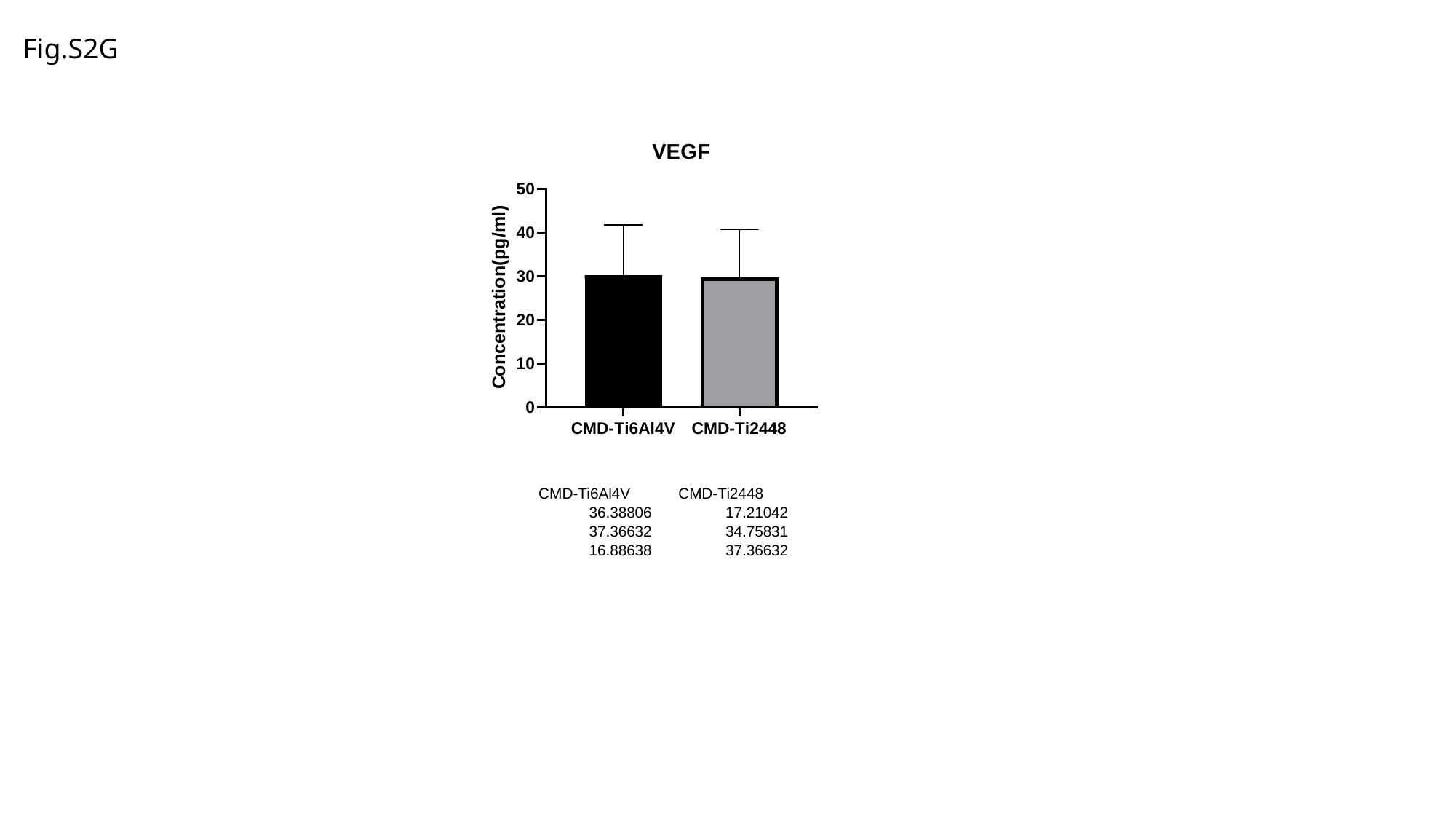

Fig.S2G
| CMD-Ti6Al4V | CMD-Ti2448 |
| --- | --- |
| 36.38806 | 17.21042 |
| 37.36632 | 34.75831 |
| 16.88638 | 37.36632 |

## Slide 13
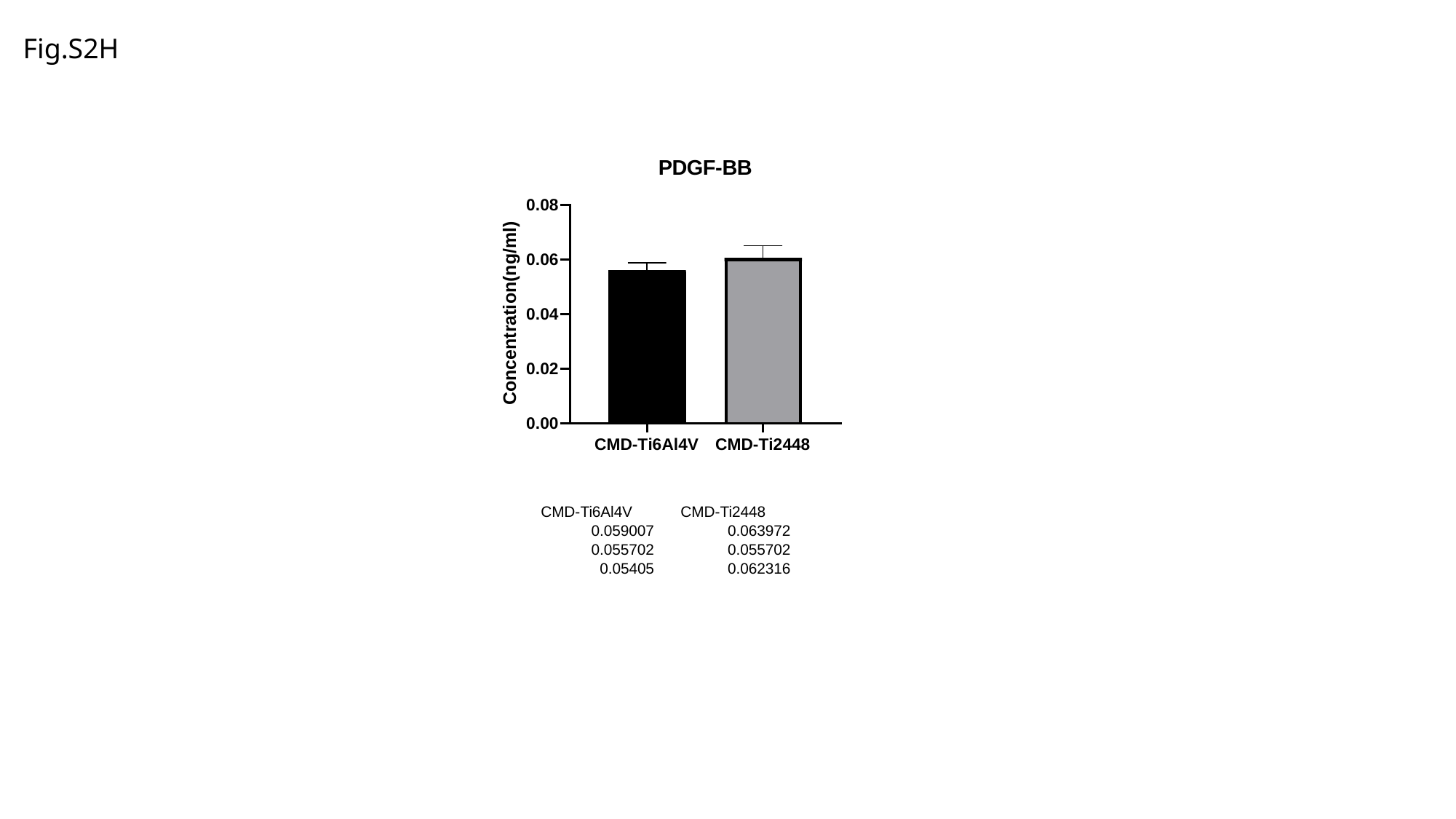

Fig.S2H
| CMD-Ti6Al4V | CMD-Ti2448 |
| --- | --- |
| 0.059007 | 0.063972 |
| 0.055702 | 0.055702 |
| 0.05405 | 0.062316 |

## Slide 14
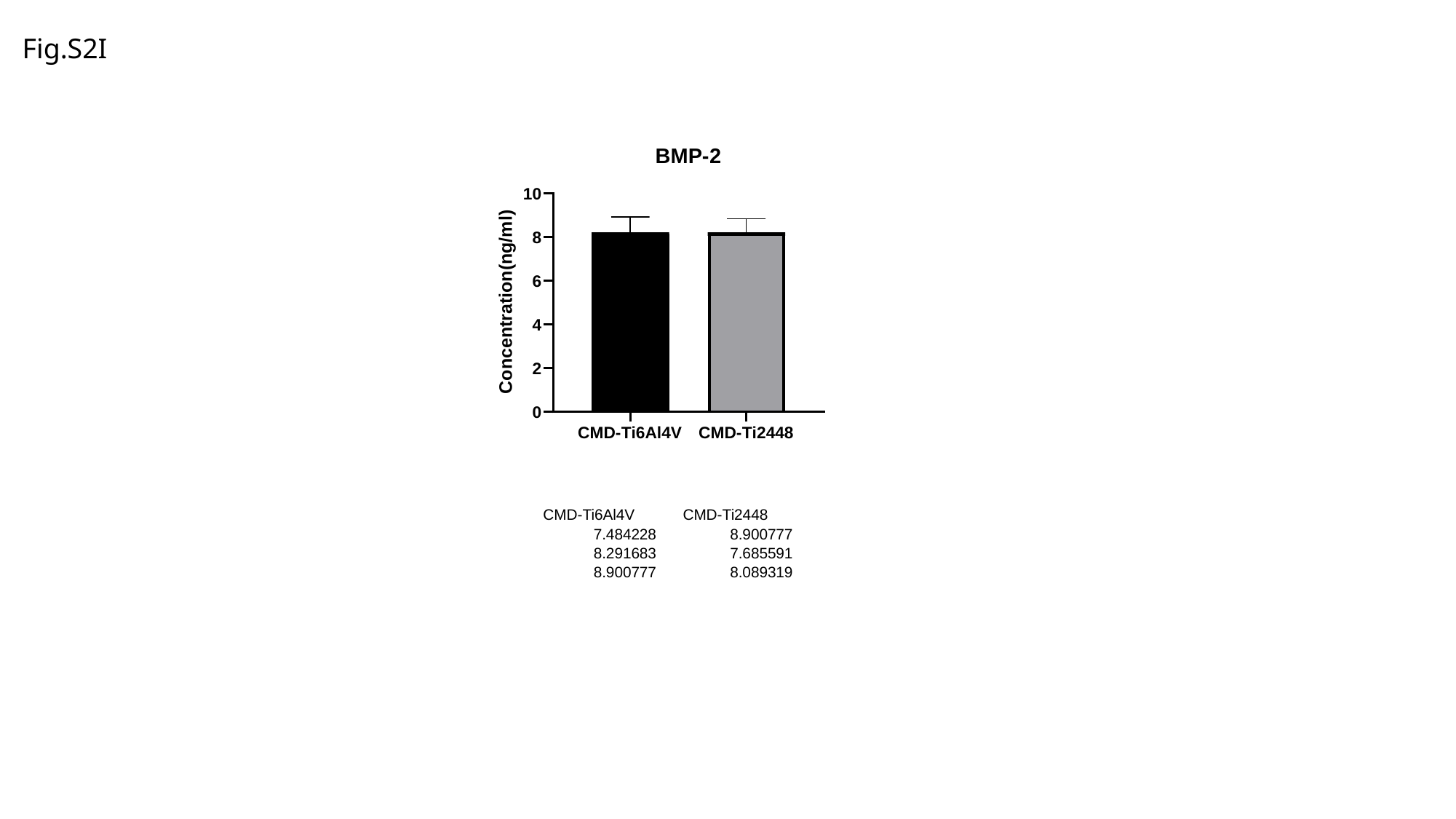

Fig.S2I
| CMD-Ti6Al4V | CMD-Ti2448 |
| --- | --- |
| 7.484228 | 8.900777 |
| 8.291683 | 7.685591 |
| 8.900777 | 8.089319 |
