# Supplementary material for: Three-Dimensionally Printed Ti2448 With Low Stiffness Enhanced Angiogenesis and Osteogenesis by Regulating Macrophage Polarization via Piezo1/YAP Signaling Axis
Source: Front Cell Dev Biol. 2021 Nov 15;9:750948. doi: 10.3389/fcell.2021.750948 (PMC8634253; doi:10.3389/fcell.2021.750948)
Supplement: Supplementary file 9 [file DataSheet10.zip › Raw data of scaffold fabrication and characterization/Raw data of scaffold fabrication and characterization .pptx]

## Slide 1
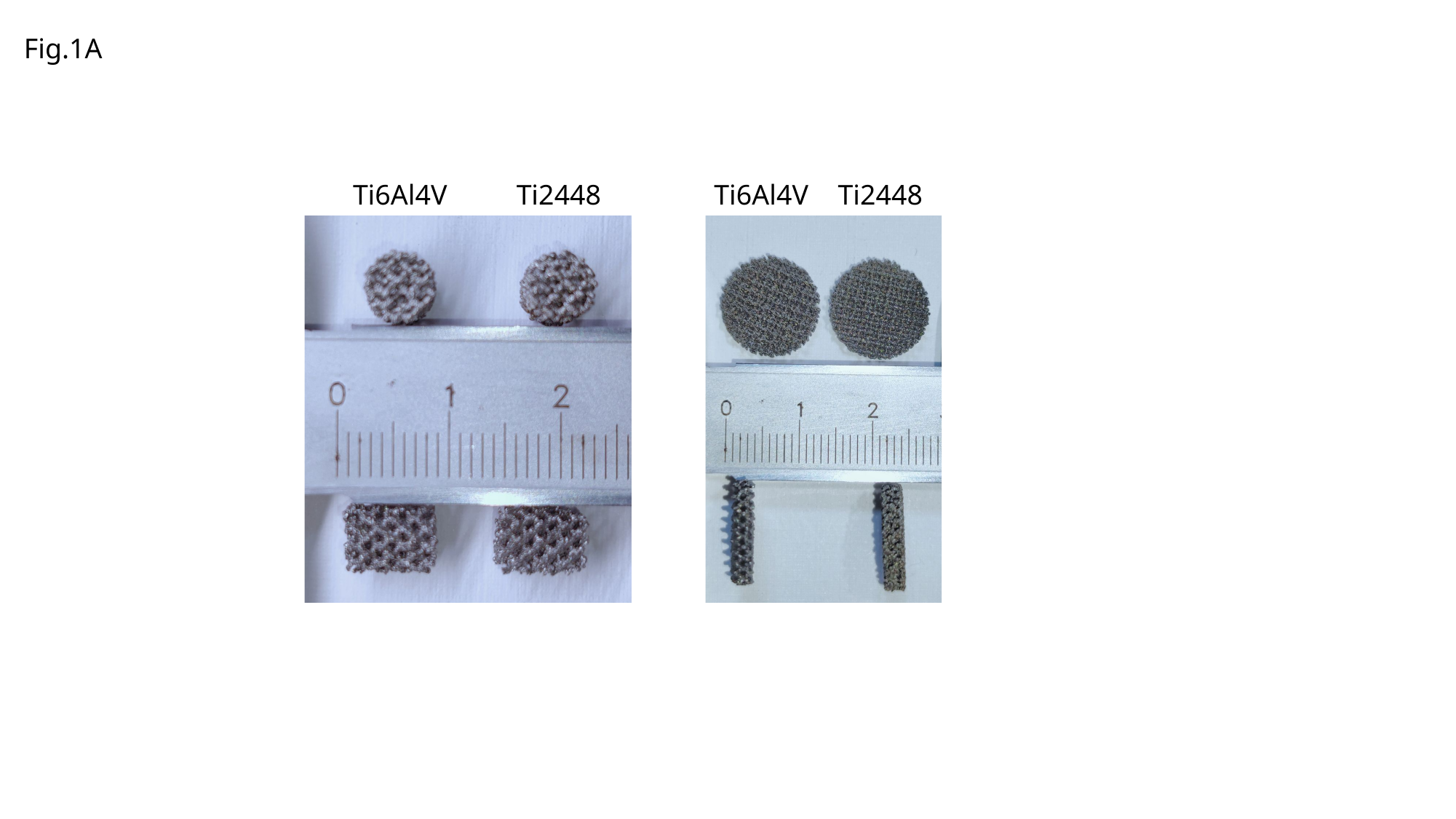

Fig.1A
Ti6Al4V
Ti2448
Ti6Al4V
Ti2448

## Slide 2
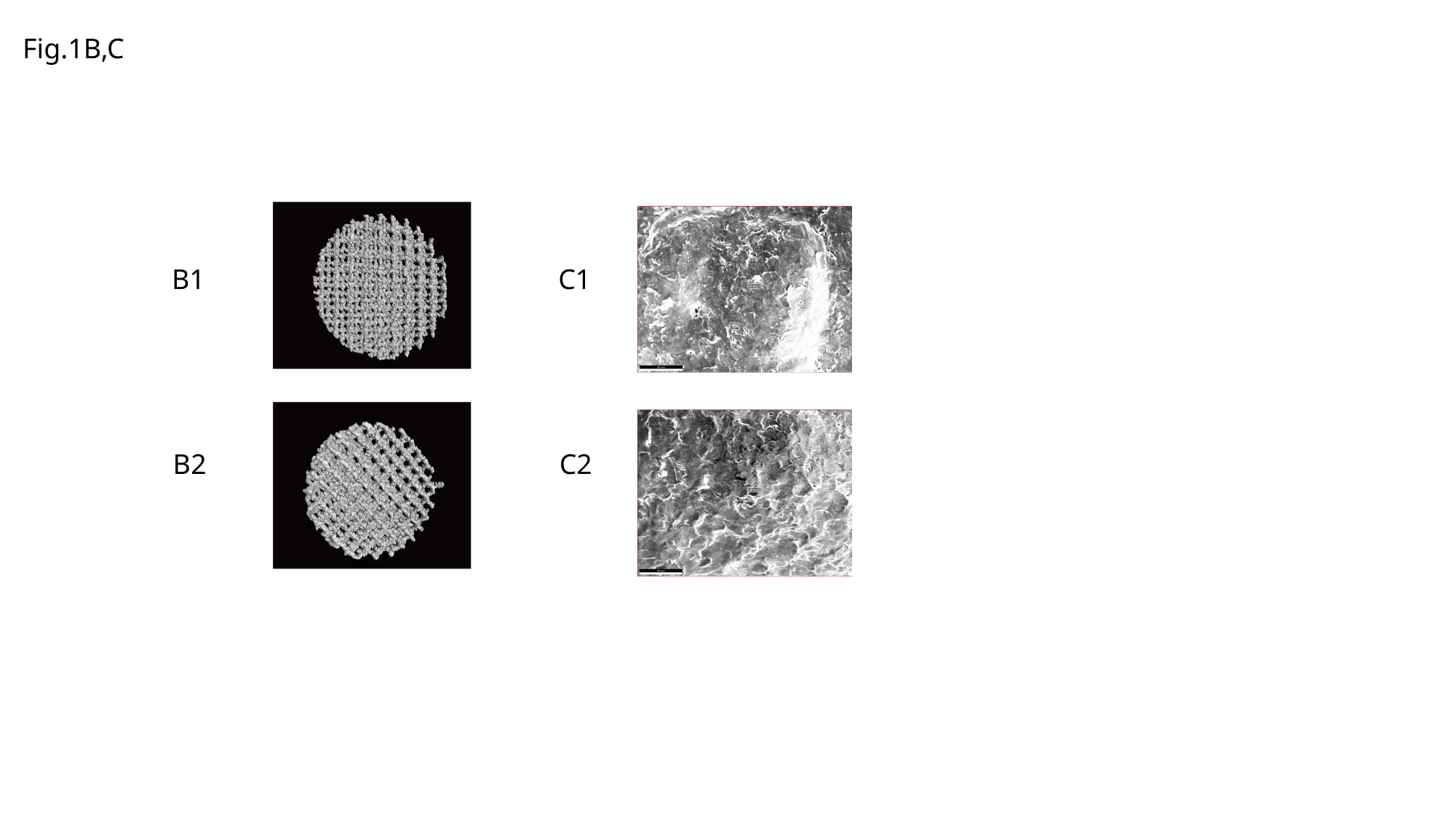

Fig.1B,C
B1
B1
C1
B2
B2
C2

## Slide 3
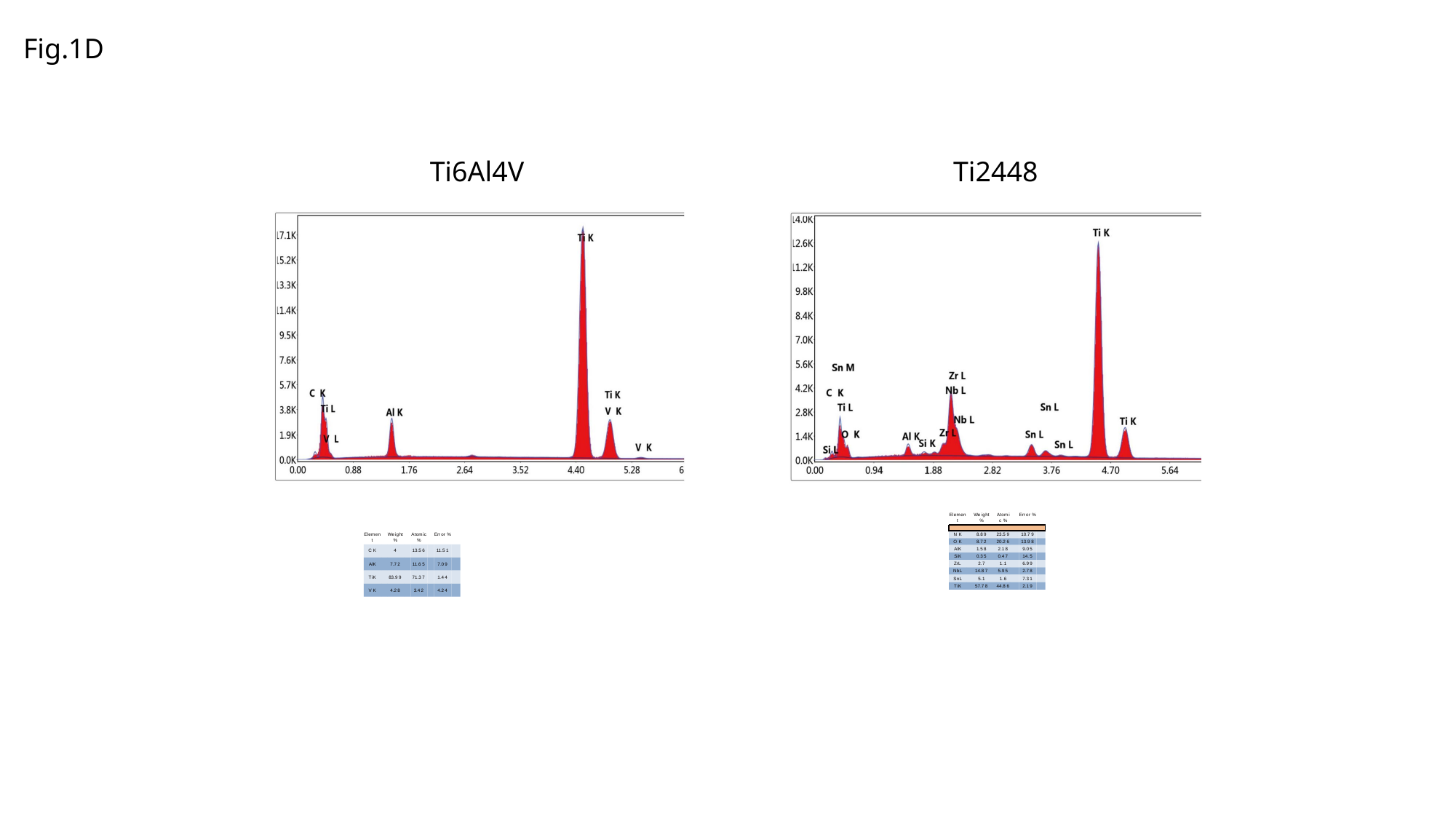

Fig.1D
Ti2448
Ti6Al4V
E
l
e
m
e
n
We
i
g
h
t
A
t
o
m
i
Err
o
r
%
t
%
c
%
23.5
9
10.7
9
N
 K
8.8
9
20.2
6
13.9
8
O
 K
8.7
2
2.1
8
AlK
1.5
8
9.0
5
SiK
0.3
5
0.4
7
14.
5
Z
rL
2.
7
1.
1
6.9
9
N
b
L
14.8
7
5.9
5
2.7
8
S
n
L
5.
1
1.
6
7.3
1
T
iK
57.7
8
44.8
6
2.1
9
E
l
e
m
e
n
We
i
g
h
t
A
t
o
m
i
c
Err
o
r
%
t
%
%
13.5
6
11.5
1
C
 K
4
11.6
5
AlK
7.7
2
7.0
9
T
iK
83.9
9
71.3
7
1.4
4
V K
4.2
8
3.4
2
4.2
4

## Slide 4
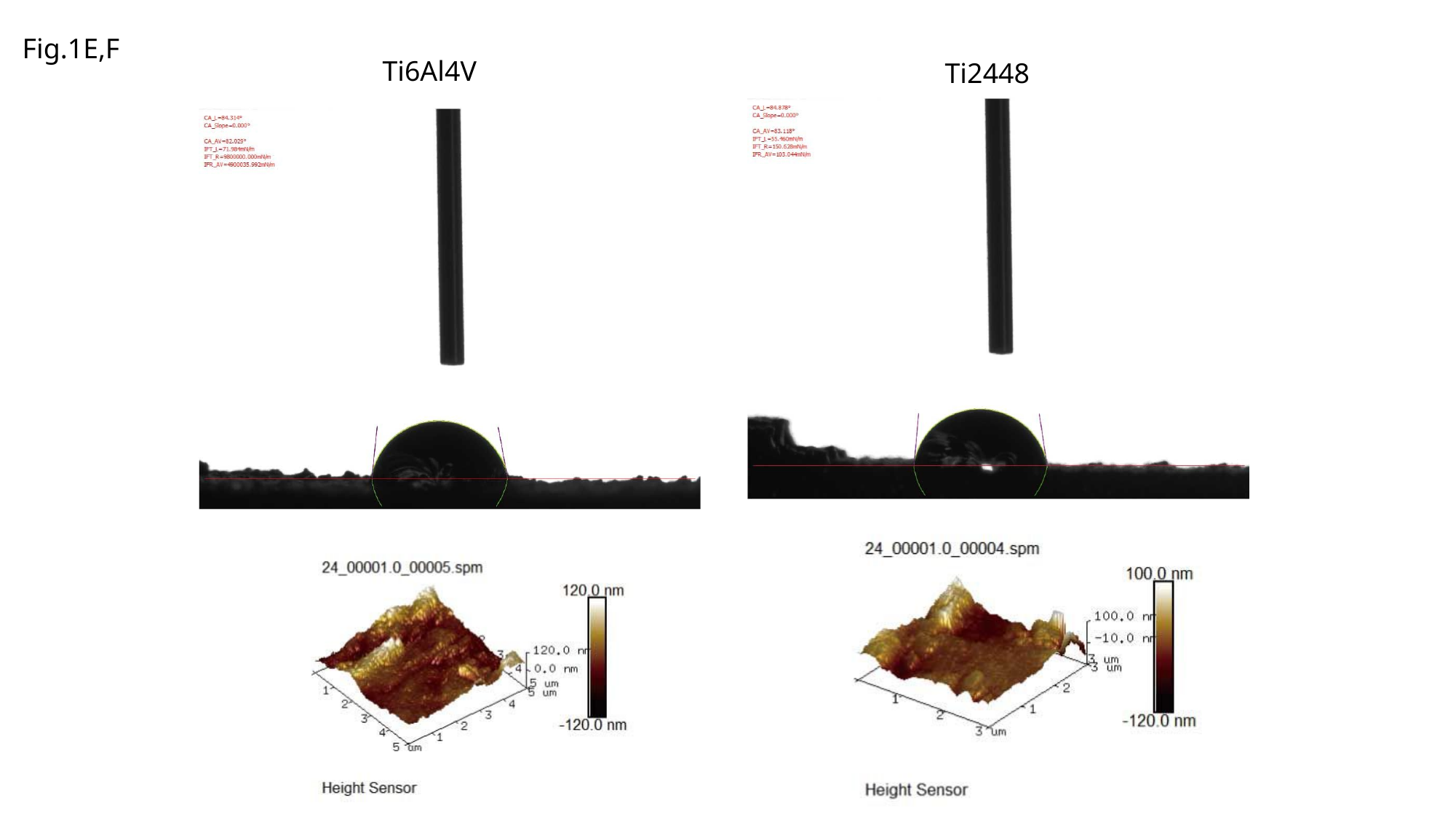

Fig.1E,F
Ti6Al4V
Ti2448
