# Supplementary material for: Three-Dimensionally Printed Ti2448 With Low Stiffness Enhanced Angiogenesis and Osteogenesis by Regulating Macrophage Polarization via Piezo1/YAP Signaling Axis
Source: Front Cell Dev Biol. 2021 Nov 15;9:750948. doi: 10.3389/fcell.2021.750948 (PMC8634253; doi:10.3389/fcell.2021.750948)
Supplement: Supplementary file 10 [file DataSheet6.zip › Raw data of immunofluorescence in vitro/Raw data of immunofluorescence in vitro.pptx]

## Slide 1
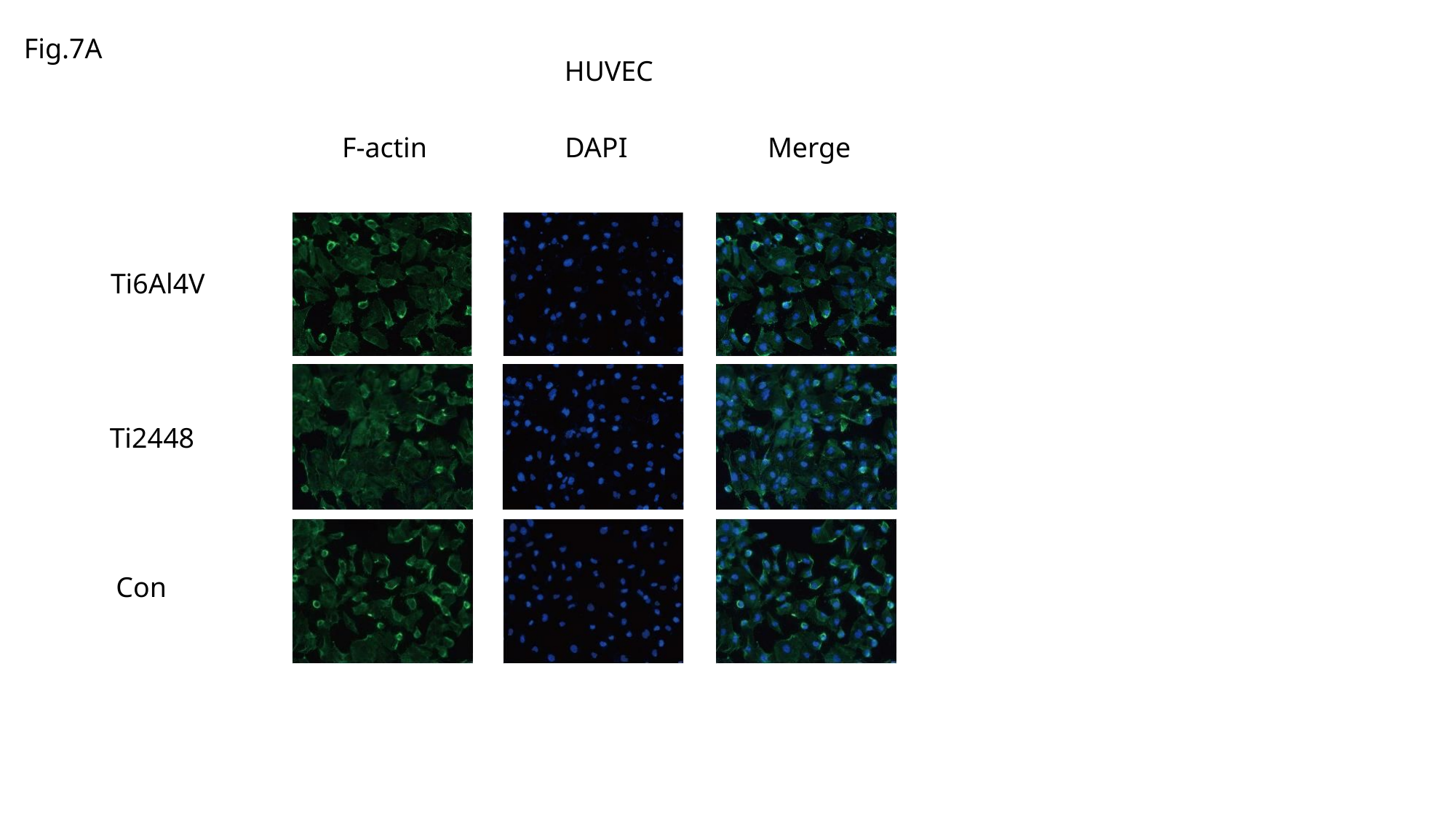

Fig.7A
HUVEC
F-actin
DAPI
Merge
Ti6Al4V
Ti2448
Con

## Slide 2
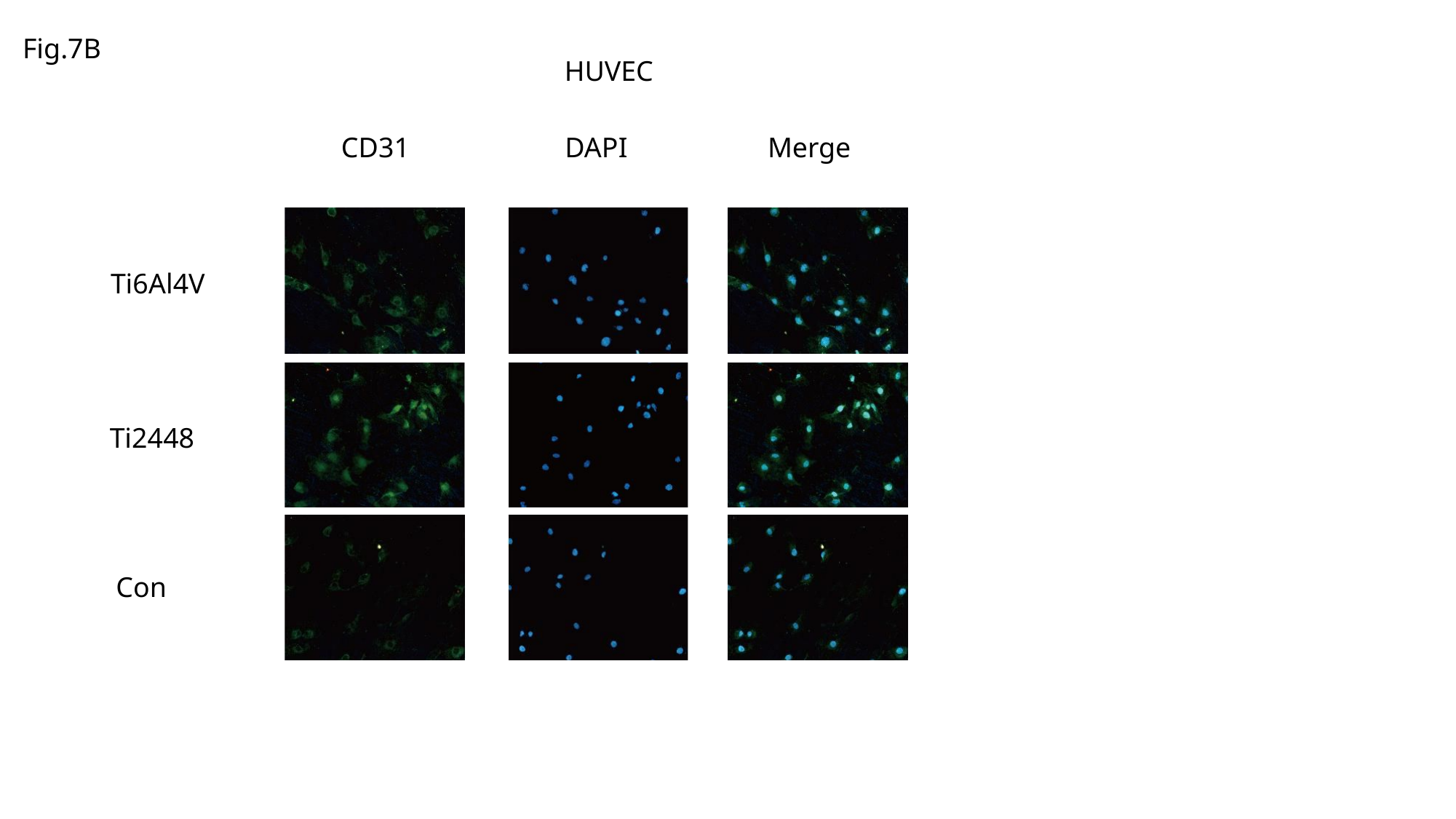

Fig.7B
HUVEC
CD31
DAPI
Merge
Ti6Al4V
Ti2448
Con

## Slide 3
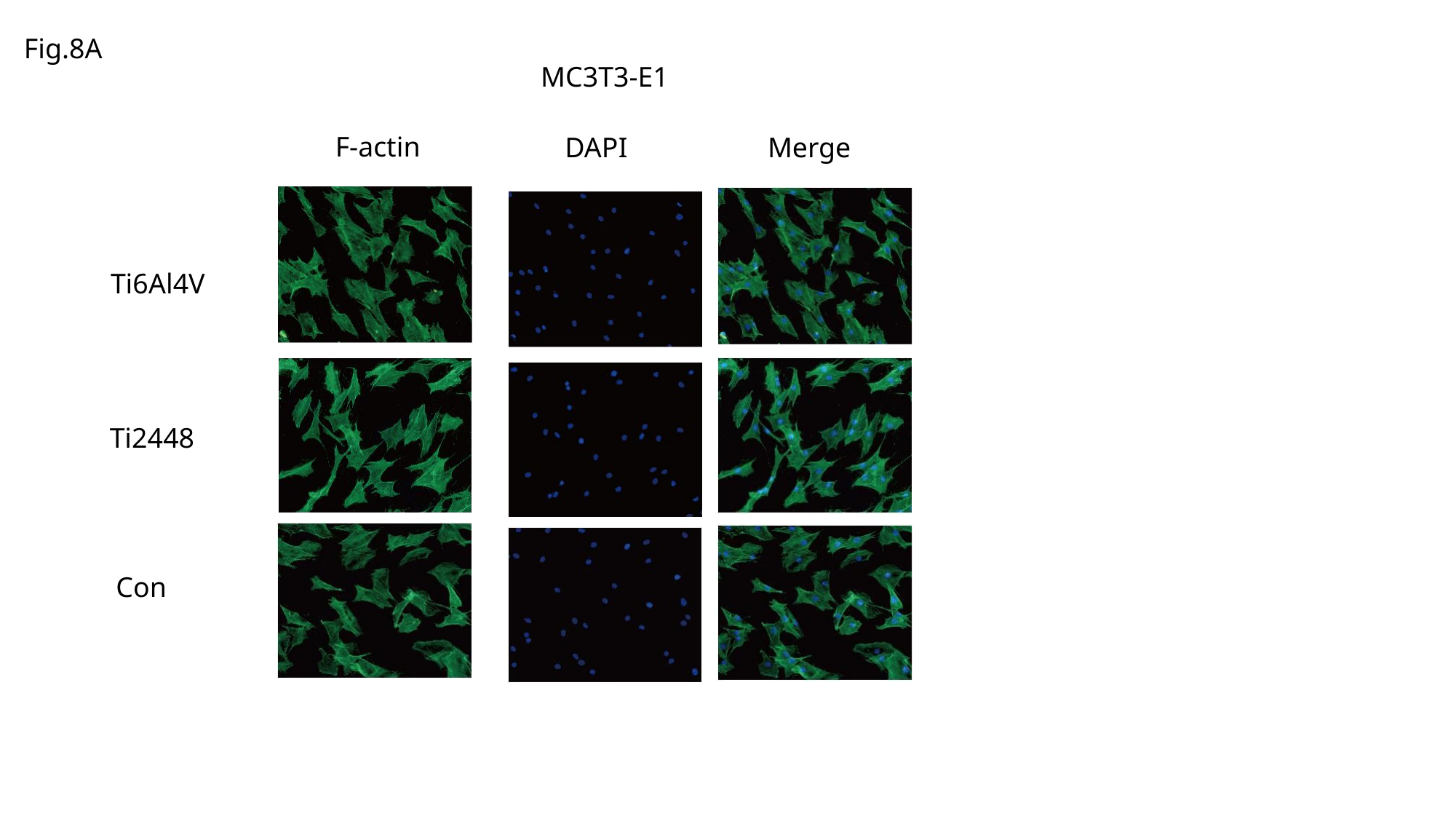

Fig.8A
MC3T3-E1
F-actin
DAPI
Merge
Ti6Al4V
Ti2448
Con

## Slide 4
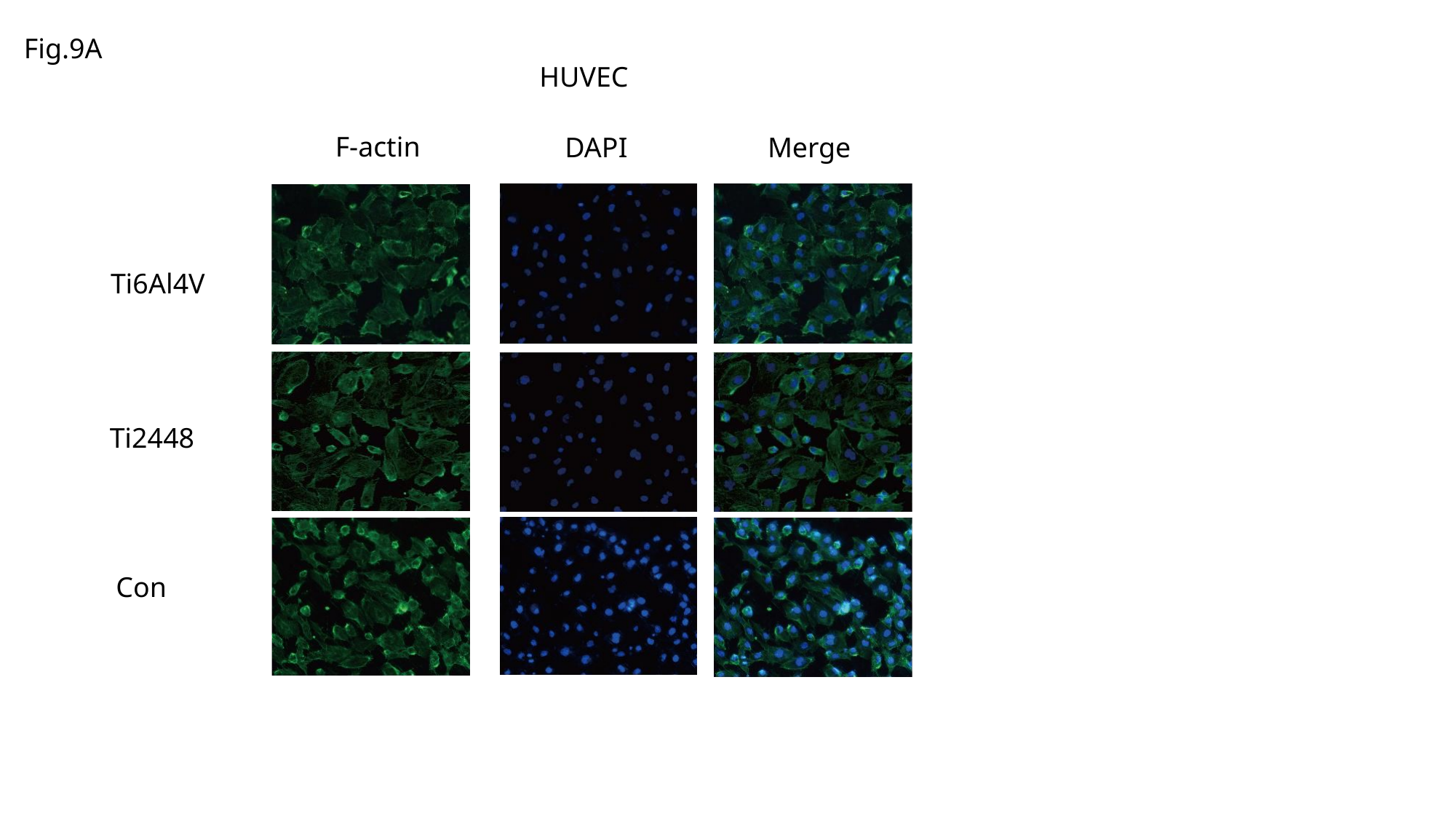

Fig.9A
HUVEC
F-actin
DAPI
Merge
Ti6Al4V
Ti2448
Con

## Slide 5
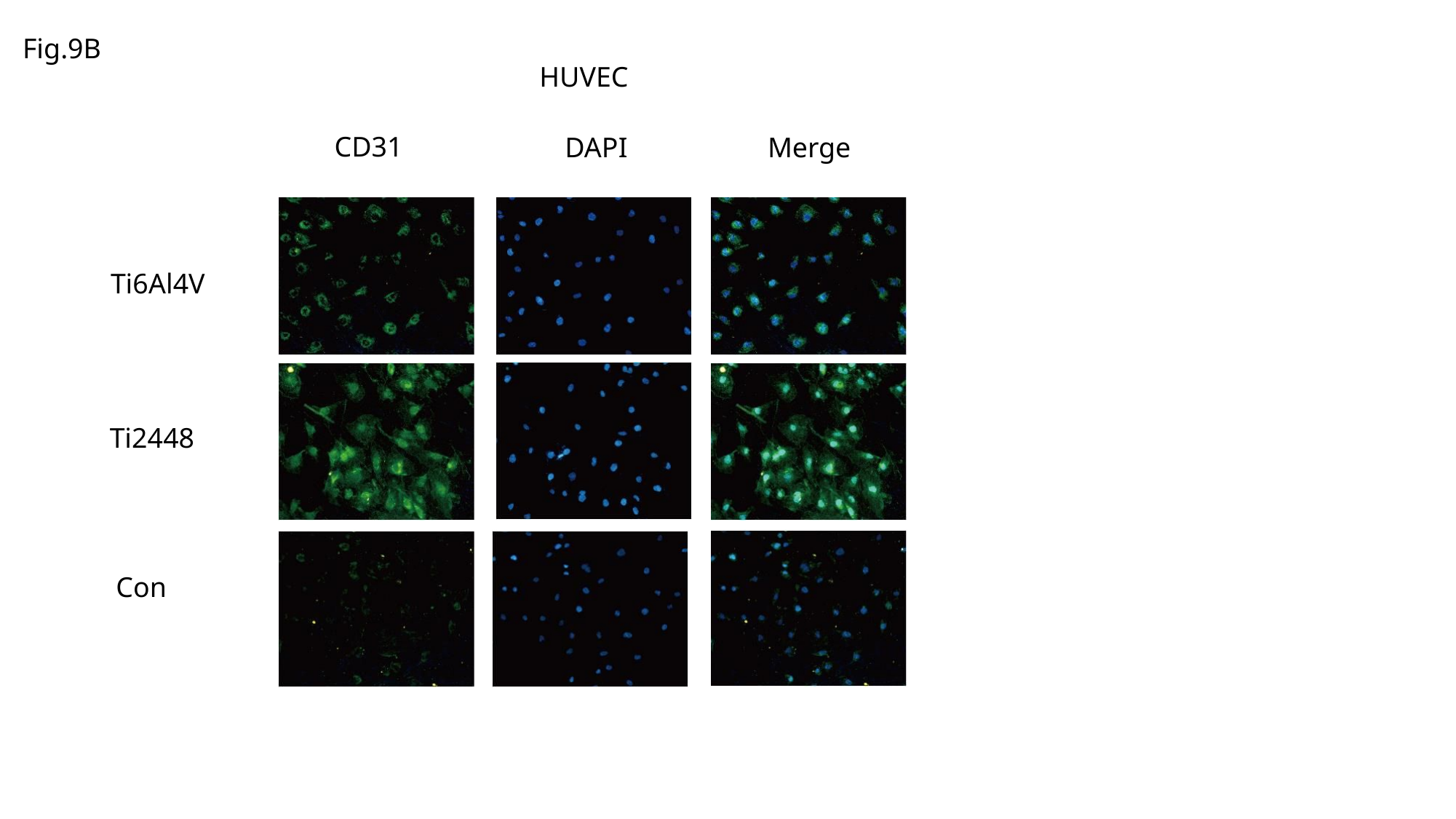

Fig.9B
HUVEC
CD31
DAPI
Merge
Ti6Al4V
Ti2448
Con

## Slide 6
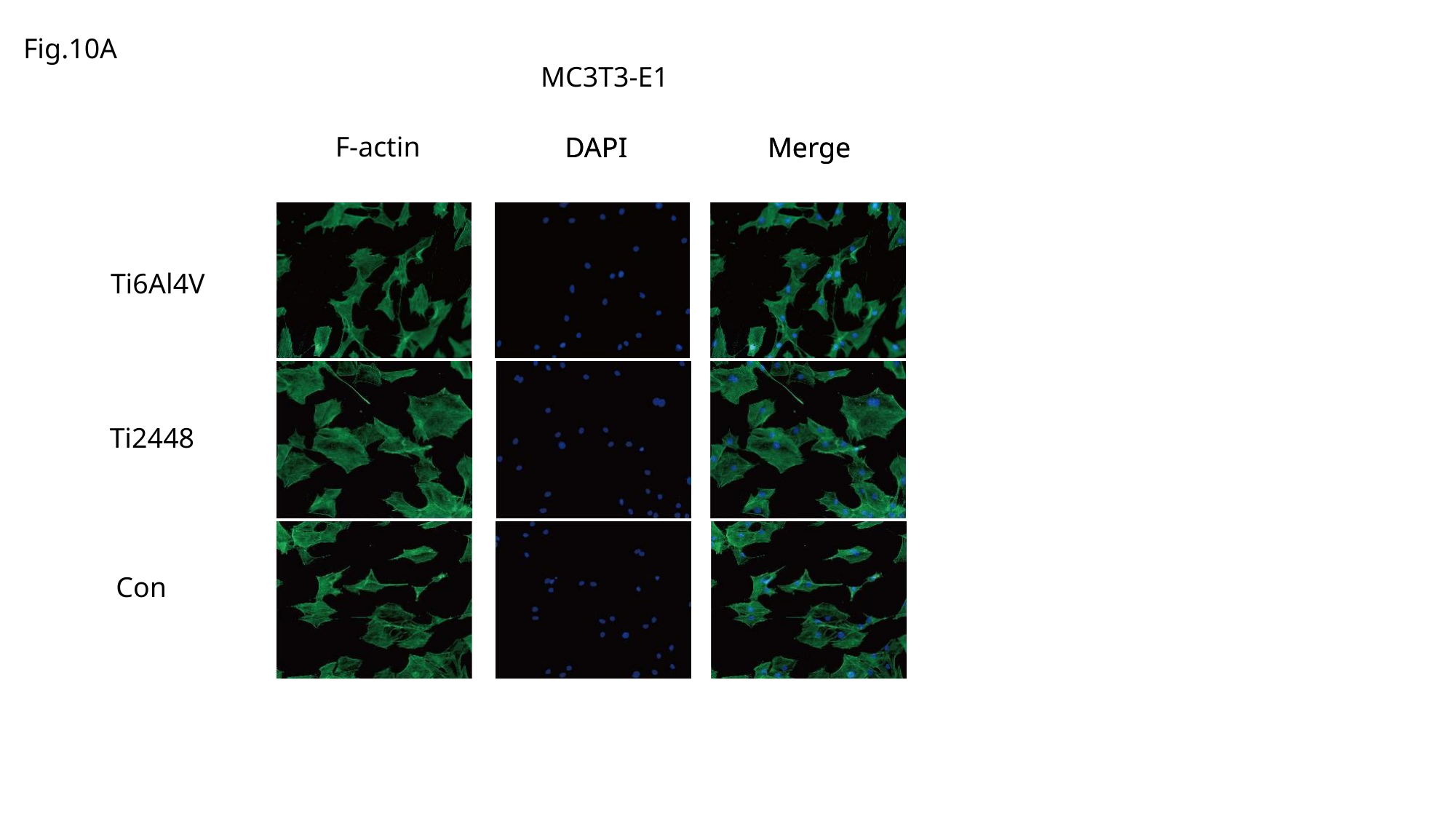

Fig.10A
MC3T3-E1
F-actin
DAPI
DAPI
Merge
Merge
Ti6Al4V
Ti2448
Con

## Slide 7
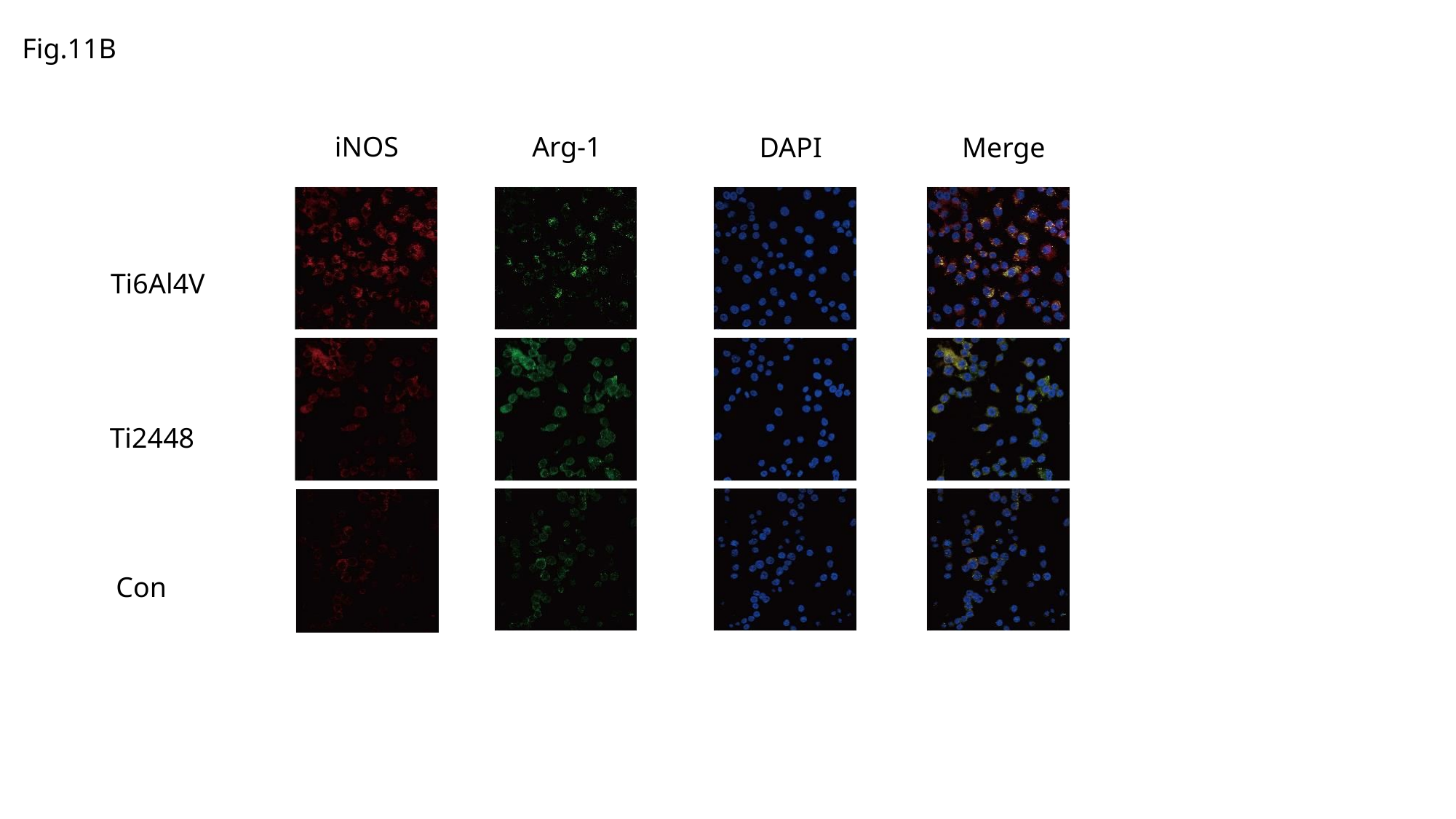

Fig.11B
iNOS
Arg-1
DAPI
Merge
Ti6Al4V
Ti2448
Con

## Slide 8
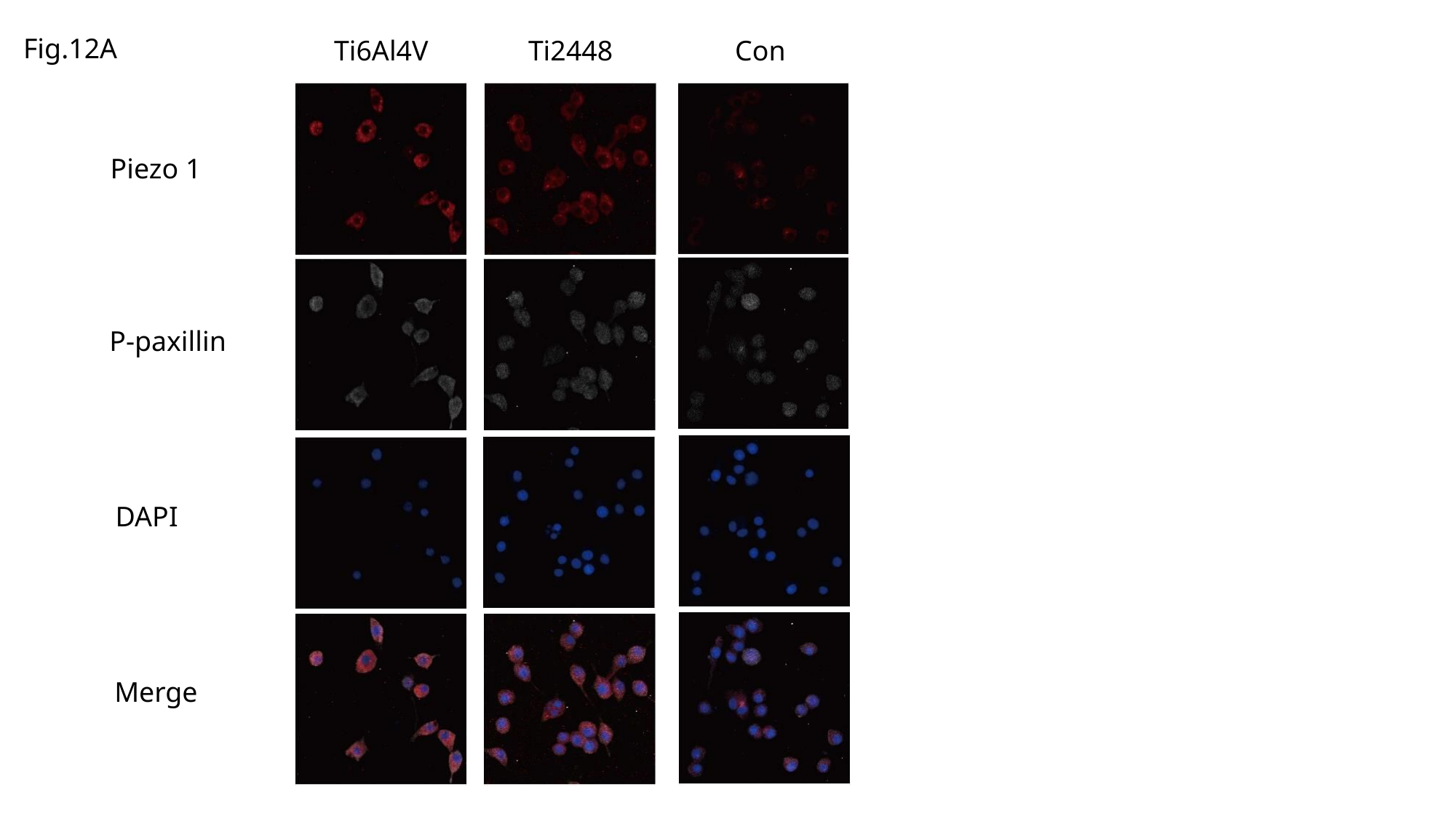

Fig.12A
Ti6Al4V
Ti2448
Con
Piezo 1
P-paxillin
DAPI
Merge

## Slide 9
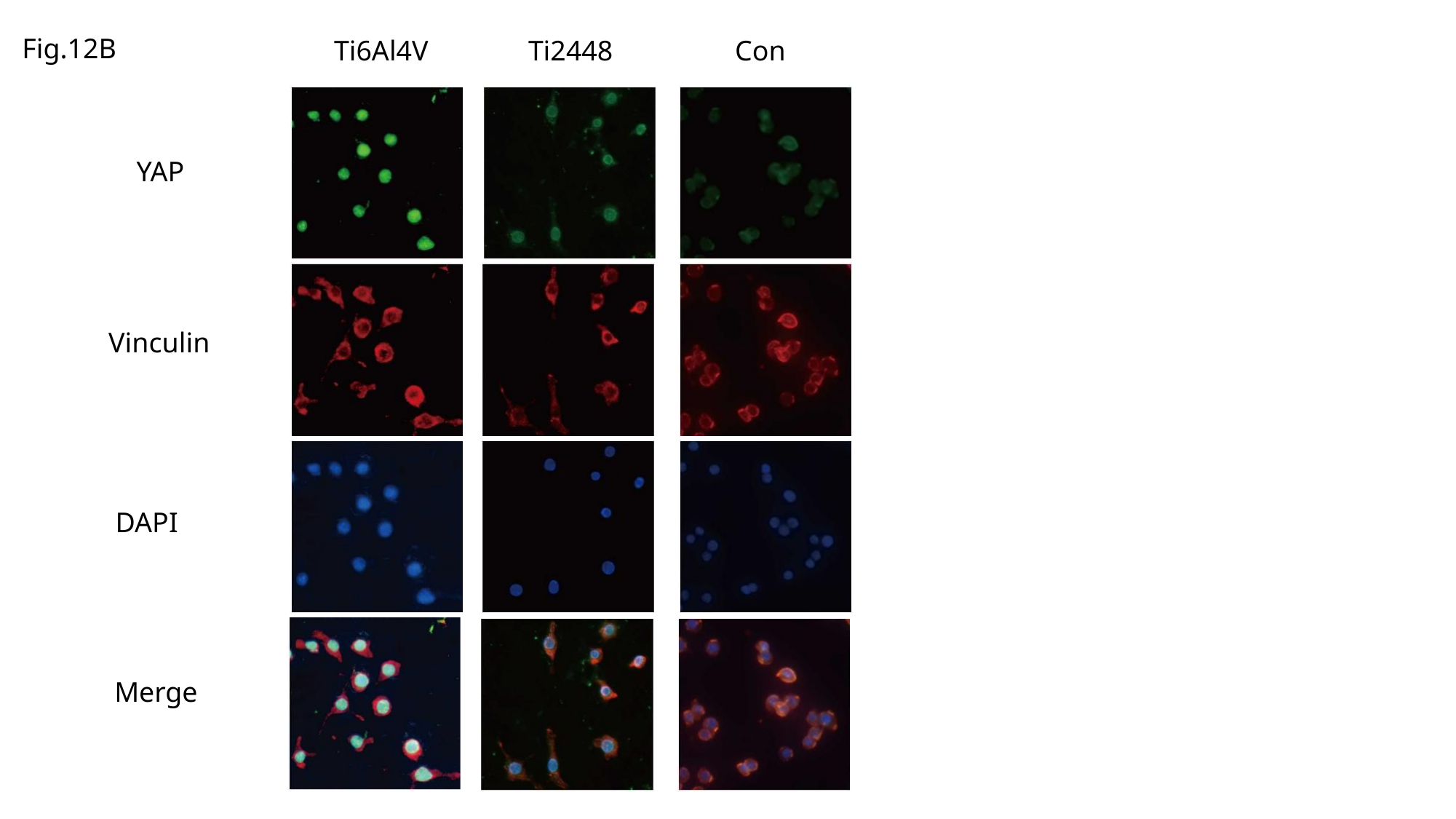

Fig.12B
Ti6Al4V
Ti2448
Con
YAP
Vinculin
DAPI
Merge

## Slide 10
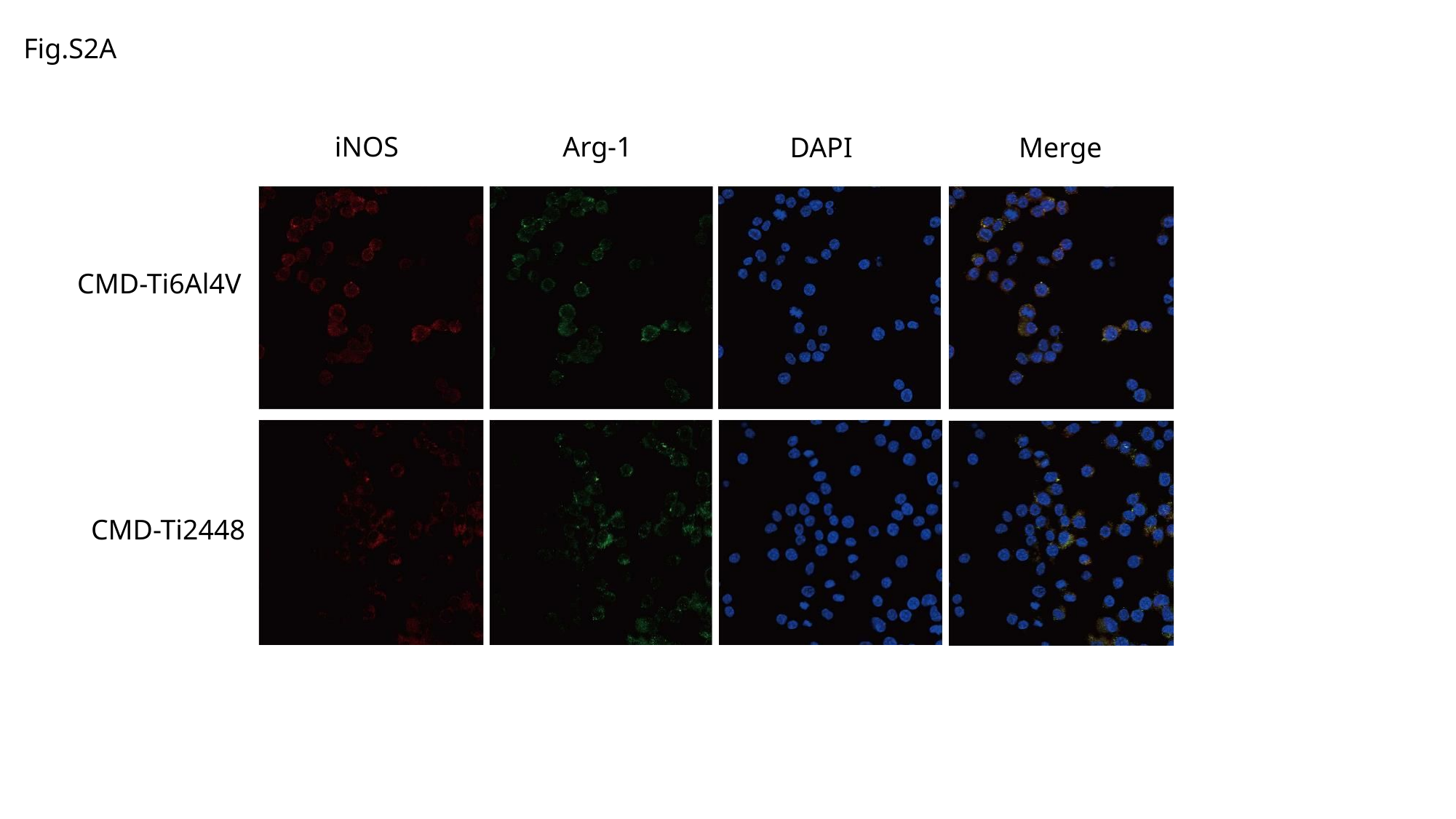

Fig.S2A
iNOS
Arg-1
DAPI
Merge
CMD-Ti6Al4V
CMD-Ti2448
