# Supplementary material for: Three-Dimensionally Printed Ti2448 With Low Stiffness Enhanced Angiogenesis and Osteogenesis by Regulating Macrophage Polarization via Piezo1/YAP Signaling Axis
Source: Front Cell Dev Biol. 2021 Nov 15;9:750948. doi: 10.3389/fcell.2021.750948 (PMC8634253; doi:10.3389/fcell.2021.750948)
Supplement: Supplementary file 13 [file DataSheet7.zip › Raw data of immunofluorescence in vivo/Raw data of immunofluorescence in vivo.pptx]

## Slide 1
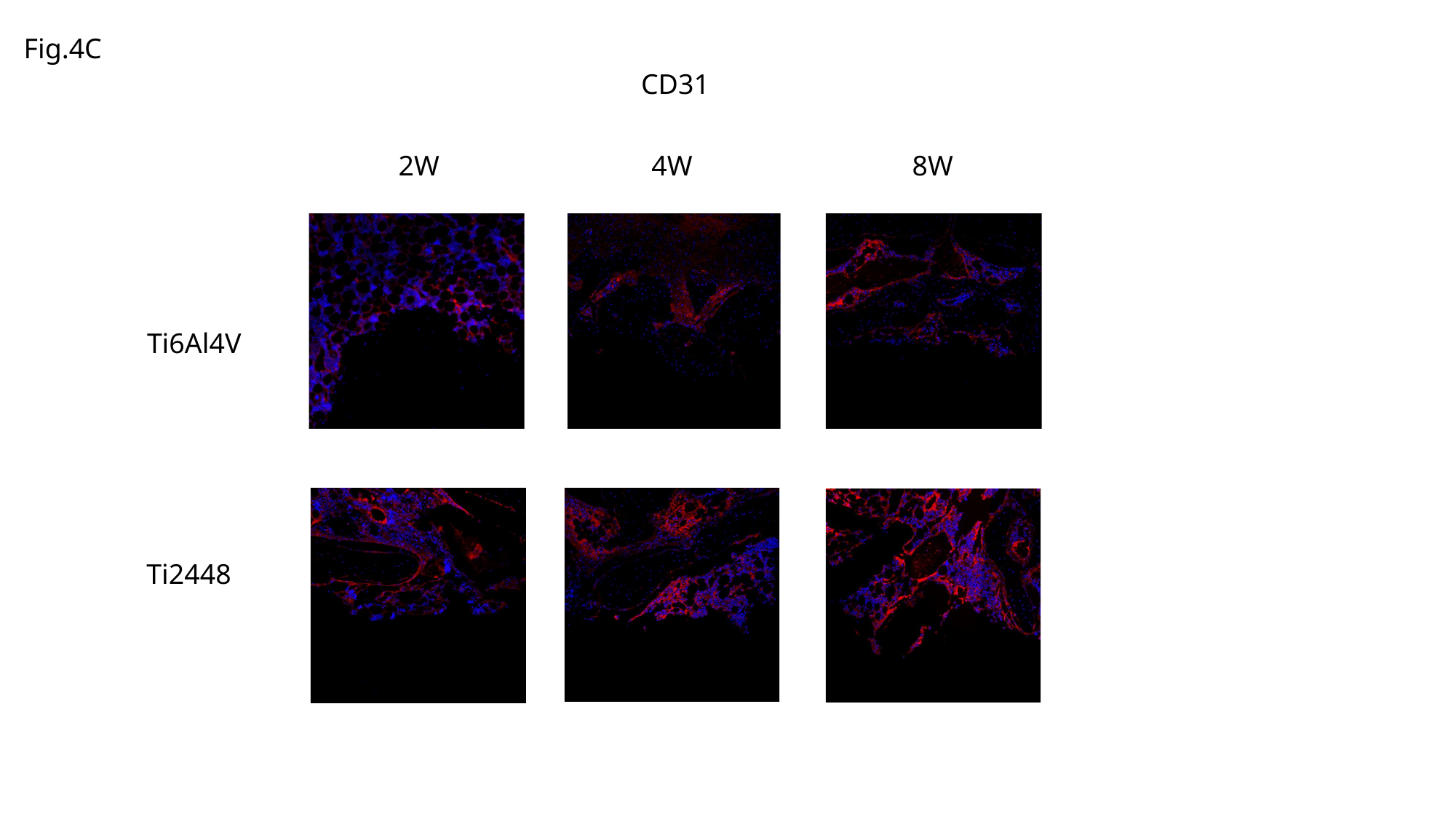

Fig.4C
CD31
2W
4W
8W
Ti6Al4V
Ti2448

## Slide 2
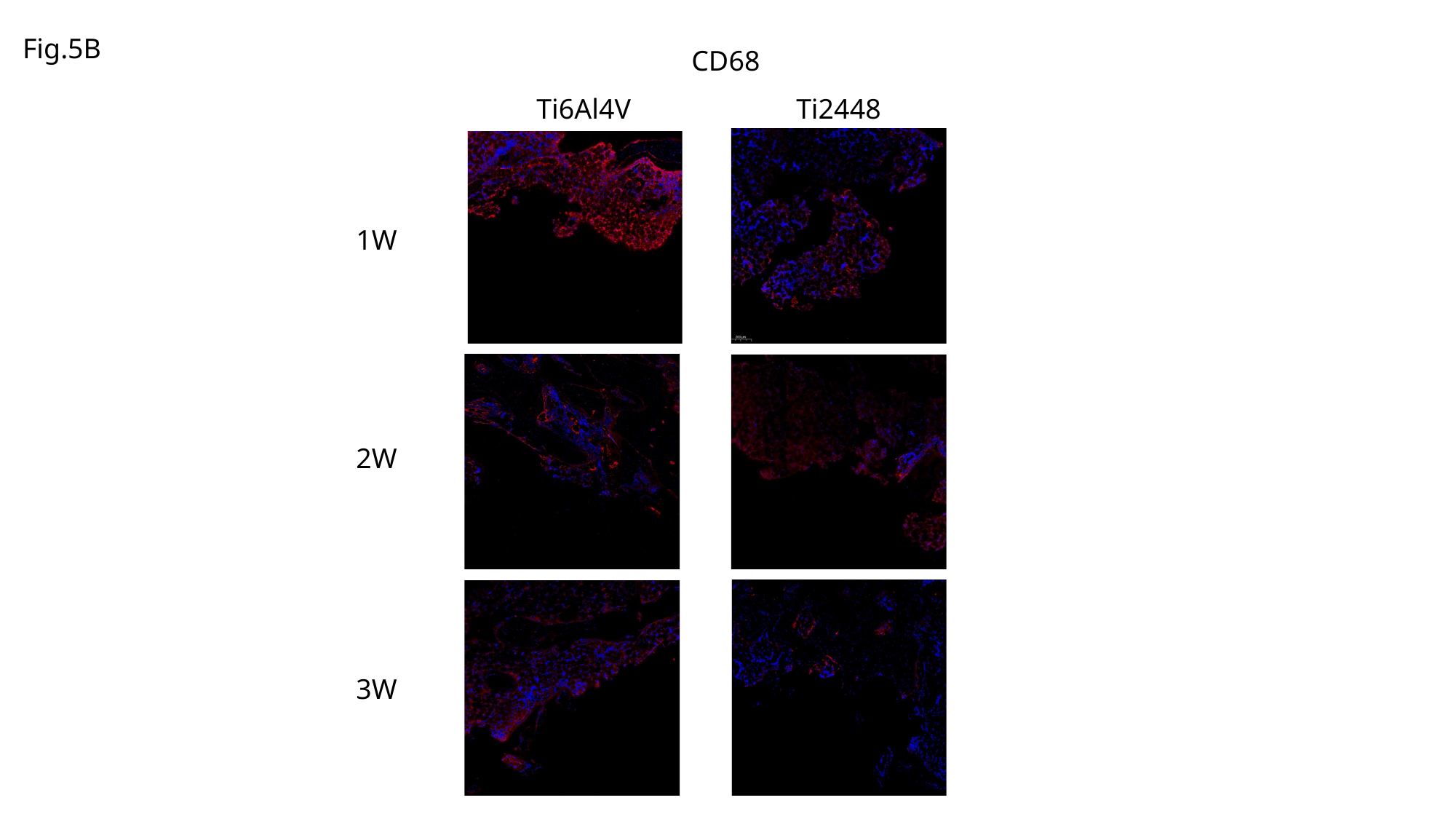

Fig.5B
CD68
Ti6Al4V
Ti2448
1W
2W
3W

## Slide 3
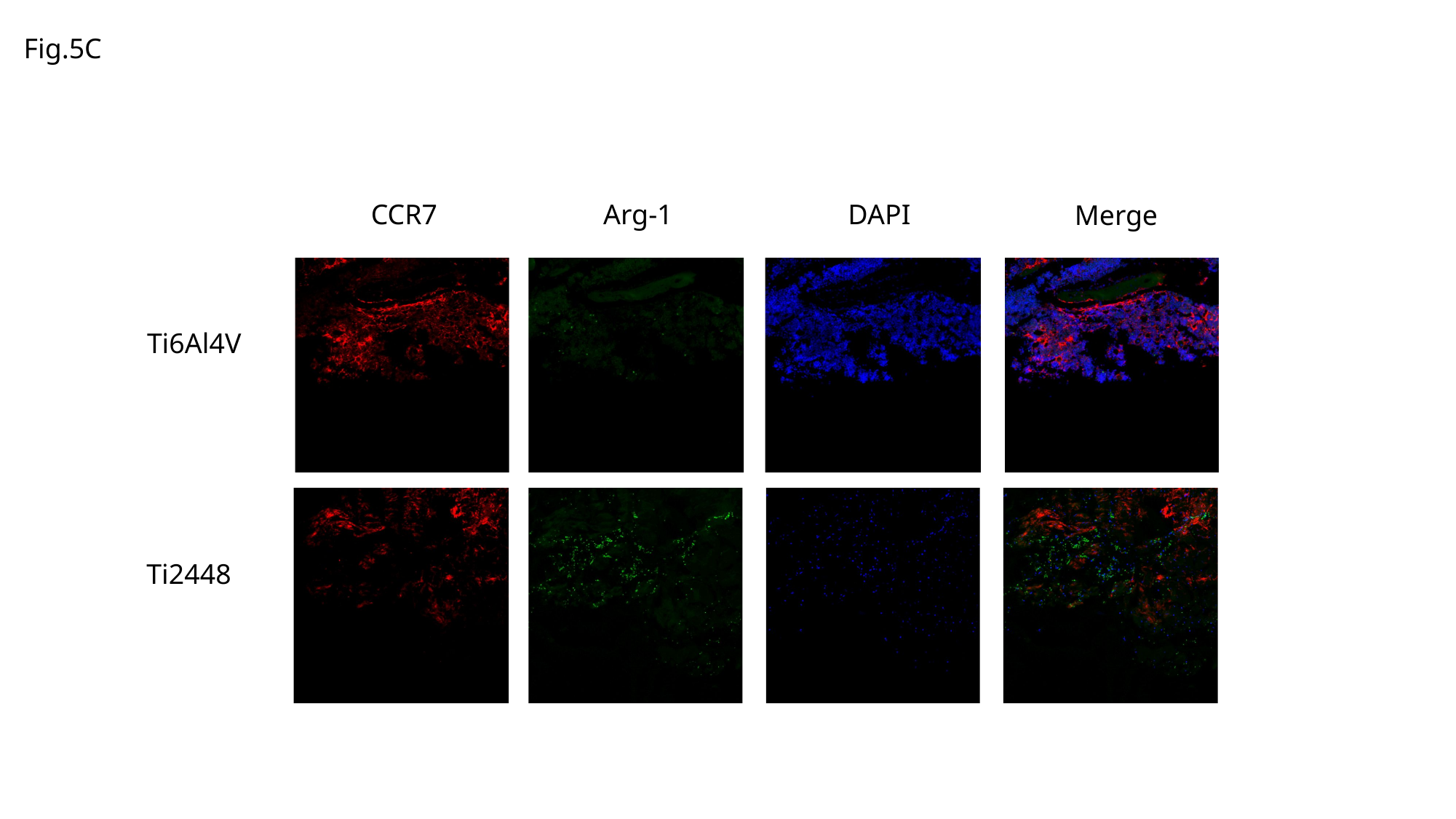

Fig.5C
CCR7
Arg-1
DAPI
Merge
Ti6Al4V
Ti2448

## Slide 4
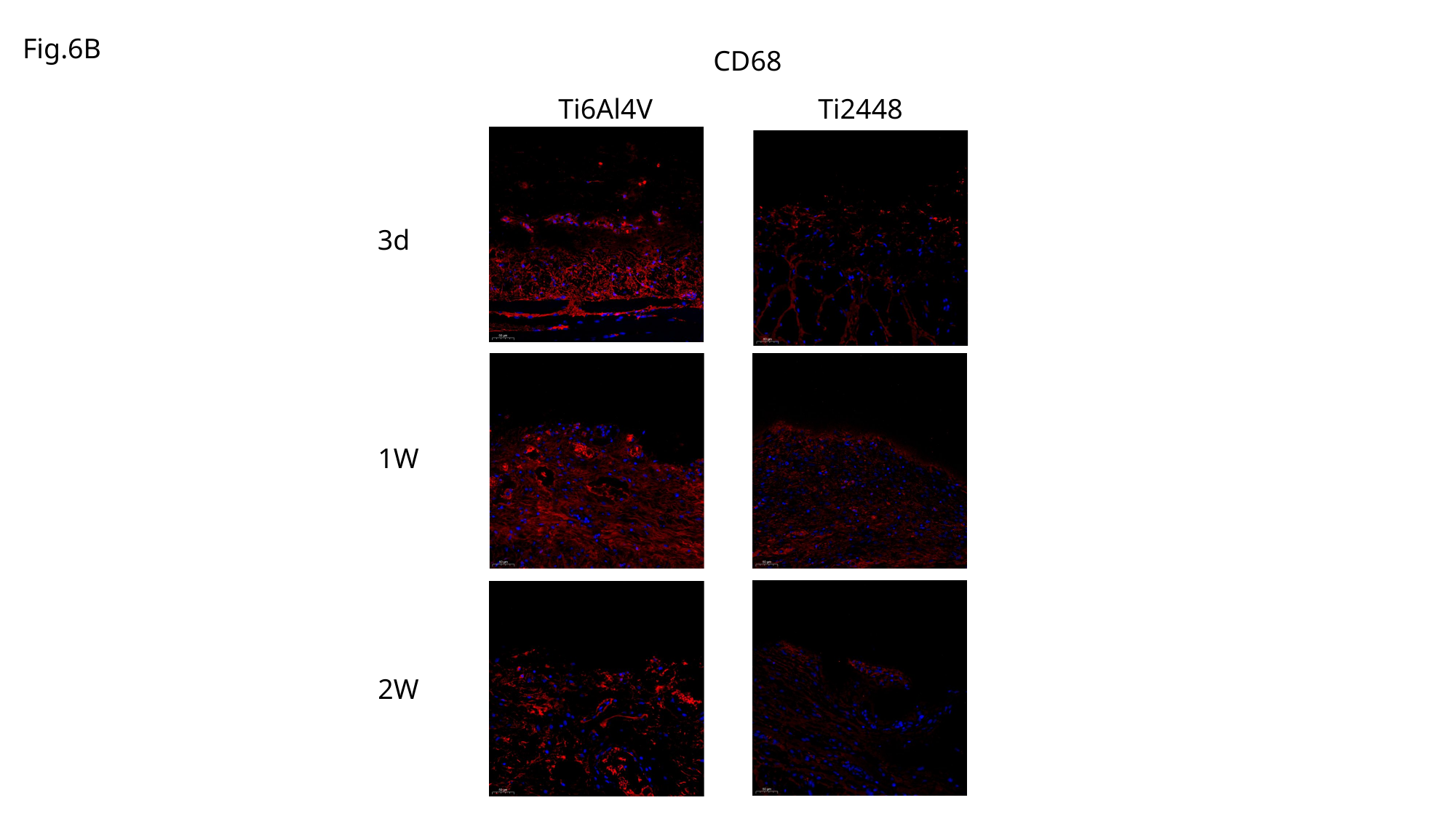

Fig.6B
CD68
Ti6Al4V
Ti2448
3d
1W
2W

## Slide 5
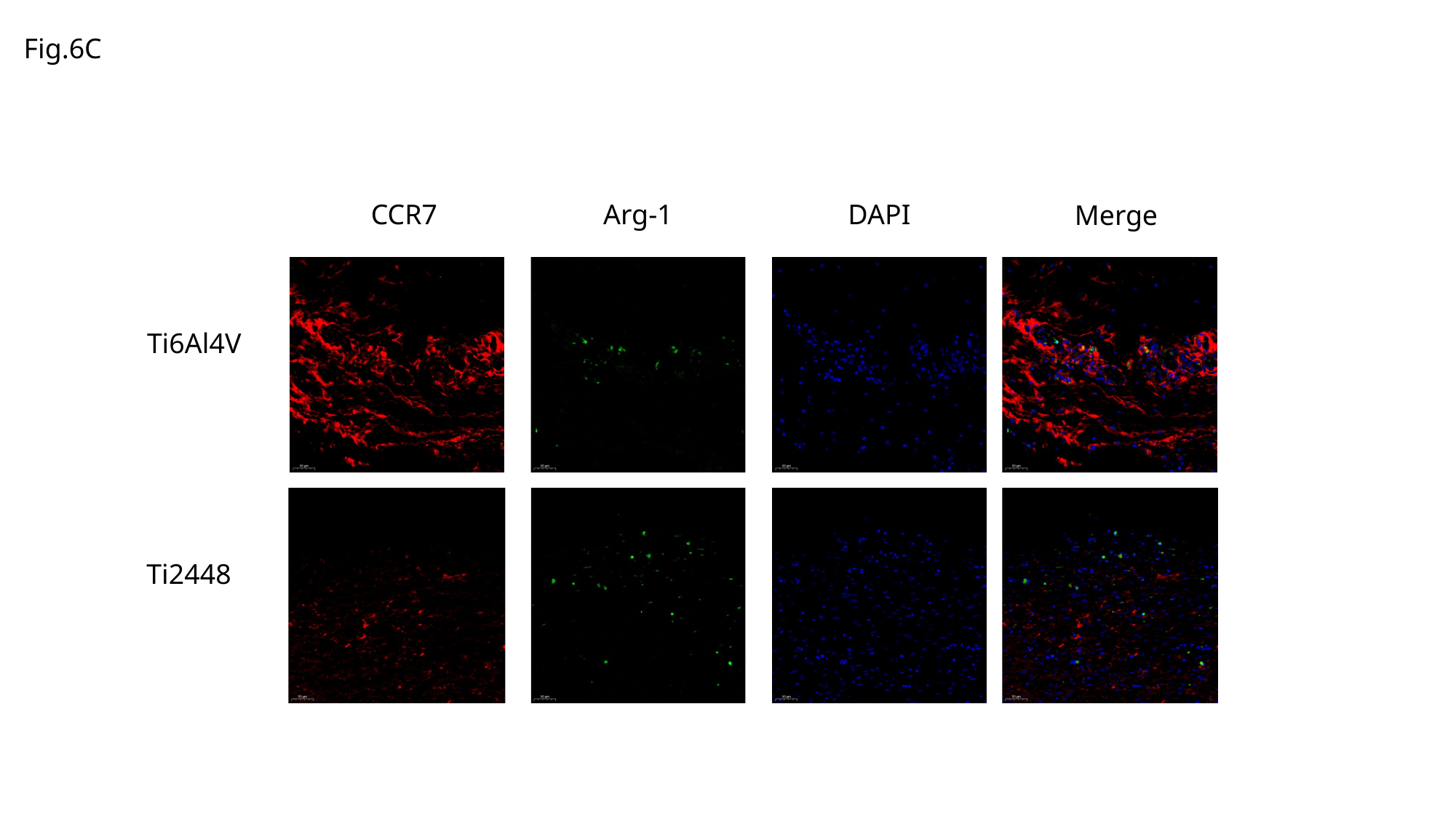

Fig.6C
CCR7
Arg-1
DAPI
Merge
Ti6Al4V
Ti2448
